# Integrated Multi-omics Data Analysis and *In Vitro* Validation Reveal the Crucial Role of Glycogen Metabolism in Gastric Cancer

**DOI:** 10.7150/jca.104424

**Published:** 2025-01-13

**Authors:** Xin Zhou, Jing Wu, Yaoyao Liu, Xiaping Wang, Xuan Gao, Xuefeng Xia, Jing Xu, Jing He, Tongshan Wang, Yongqian Shu

**Affiliations:** 1 Department of Oncology, First Affiliated Hospital of Nanjing Medical University, Nanjing 210029, China.; 2 Department of Oncology, The Affiliated Suqian First People's Hospital of Nanjing Medical University, Suqian 223812, China.; 3 Beijing GenePlus Genomics Institute, Beijing, 102205, China.; 4 Department of Pathology, The Second Affiliated Hospital of Nanjing Medical University, Nanjing 210000, China.; 5 State Key Laboratory of Microbial Resources, Institute of Microbiology, Chinese Academy of Sciences, Beijing, 100101, China.; 6 Shenzhen GenePlus Clinical Laboratory, ShenZhen, 518122, China.; 7 Jiangsu Key Lab of Cancer Biomarkers, Prevention and Treatment, Collaborative Innovation Center for Cancer Personalized Medicine, Nanjing Medical University, Nanjing 210029, China.

**Keywords:** glycogen metabolism, gastric cancer, single-cell sequencing, prognosis, immunotherapy

## Abstract

**Background:** This study aimed to investigate glycogen metabolism in gastric cancer (GC) and develop a glycogen-based riskScore model for predicting GC prognosis.

**Methods:** Patients' expression profiles for 33 tumor types were retrieved from TCGA. Four GC bulk and one single-cell sequencing datasets were obtained from GEO database. This study also enrolled a bladder urothelial carcinoma immunotherapeutic IMvigor210 cohort. The ssGSEA method was conducted to assess glycogen biosynthesis and degradation level. Consensus clustering analysis was conducted to identify different clusters. A glycogen riskScore signature was developed to evaluate prognostic value across different cohorts. Besides, *in vitro* experiments were conducted to further evaluate the role of glycogen metabolism related genes in GC.

**Results:** Both glycogen biosynthesis and degradation were significantly associated with worse overall survival and were also related with malignant phenotype in GC at both bulk and single-cell levels. Differential outcomes and immune functions were verified in the three identified clusters. The constructed glycogen riskScore model accurately classified GC patients with different outcomes, genomic and immune landscape, and performed well in predicting prognosis through external validation, immunotherapy and pan-cancer cohorts. Furthermore, the riskScore could predict response to chemotherapy and immunotherapy. Functional analyses revealed the signature's connection to pro-tumor and immunosuppression related pathways across pan-cancer. Additionally, glycogen metabolism related genes were found to regulate the malignant phenotypes of GC cells.

**Conclusion:** This study revealed important roles of glycogen metabolism in promoting progression of GC and presented a glycogen riskScore model as a novel tool for predicting prognosis and treatment response.

## Introduction

Gastric cancer (GC) is a fatal malignancy that ranks second in cancer-associated mortality worldwide 1. Many GC patients are diagnosed with advanced stages due to lack of apparent symptoms, resulting in a low 5-year survival rate 2. Nowadays, immunotherapy has emerged as a recent breakthrough for cancer treatment and has been found to be an effective strategy. However, it has shown limited effectiveness in treating GC patients 3. Hence, identifying reliable prognostic biomarkers is crucial for risk stratification and improving clinical outcomes in GC patients.

Recently, many studies have investigated the role of glucose metabolism in cancer development, notably the Warburg effect which drives glycolysis for tumor cell energy 4-6. As a large and branched polymer of glucose, glycogen acts as a glucose store within the cell. Although in the 1980s, it was observed that glycogen was abundant in several cancer cell lines 7, few studies have explored the impact of glycogen metabolism in cancer. However, recent studies have highlighted glycogen's significance in aiding cell survival in nutrient-deprived and hypoxic tumor microenvironments (TME) 8-12. In GC, increased glycogen degradation could promote survival and inhibit apoptosis 13, while mobilization aid metastasis 14. These findings underscore the crucial role of glycogen metabolism and its potential as a therapeutic target in cancer, although deeper insights into its interactions during carcinogenesis are still needed.

Using diverse data sources (genomics, transcriptomics, single-cell sequencing, and *in vitro* experiments), this study unveiled the crucial role of glycogen metabolism in pan-cancer, with a particular focus on GC. A glycogen riskScore model was built via consensus clustering and least absolute shrinkage and selection operator (LASSO) Cox regression. This model could predict chemotherapy and immunotherapy responses, and serve as an independent indicator for GC prognosis. Functional analyses additionally highlighted the close interactions of the model with TME as well as immune reaction. Moreover, the credibility and functional roles of the glycogen riskScore signature were further confirmed in pan-cancer. Besides, *in vitro* experiments revealed the crucial roles of glycogen metabolism related genes in the progression of GC.

## Methods

### Data collection

Transcriptome profiles with clinical data were downloaded from the The Cancer Genome Atlas (TCGA; https://portal.gdc.cancer.gov/) and GEO (http://www.ncbi.nlm.nih.gov/geo/). For GC, three mRNA microarray datasets (GSE57303, GSE62254 and GSE84437) and TCGA- stomach adenocarcinoma (STAD) data were merged into a meta-cohort. An independent validation cohort (GES15459) was sourced for assessing the model. The batch effects of TCGA and GEO datasets were removed by the 'combat' function of the R package 'sva'. The immunotherapeutic IMvigor210 cohort data were downloaded using the “IMvigor210CoreBiology” R package (http://research-pub.gene.com/IMvigor210CoreBiologies/) 15. Additionally, single-cell transcriptome data (GSE163558) from GC patients' samples (three primary tumor (PT), one adjacent non-tumor (NT), and six metastatic samples (M)) were obtained from GEO 16.

### Single-cell RNA sequencing data and analysis

The Seurat package (version 4.1.3) was applied to perform single-cell RNA sequencing (scRNA-seq) analysis 17. Transcriptomes were filtered with parameters as min. cells = 3 and min. features = 250, the proportion of mitochondrial genes < 35% of counts, and the number of cell genes ranged from 500 to 6,000. After normalization and centralization of the data, we identified hypervariable genes and applied principal component analysis (PCA) followed by t-distributed stochastic neighbor embedding (tSNE) dimensionality reduction for unsupervised clustering and unbiasedly visualizing cell populations on a two-dimensional map. Cell subsets were reannotated by SingleR package via verification of the expression of established markers specific to diverse cell clusters. The marker genes for each cell subset clusters were conducted by "FindAllMarkers" function to identify differentially expressed genes (DEGs) of each cell type. “FeaturePlot” and “VlnPlot” were applied to visualize the levels of glycogen metabolism or gene expression in different subsets of cells.

### Functional enrichment analysis

Single-sample gene set enrichment analysis (ssGSEA) algorithms were applied to quantify the enrichment score of each sample or single-cell in glycogen biosynthesis and degradation pathways (gene sets from Rosario's study), fourteen programmed cell death (PCD) patterns (intrinsic apoptosis, extrinsic apoptosis, necroptosis, pyroptosis, ferroptosis, cuproptosis, entotic cell death, netotic cell death, parthanatos, lysosome-dependent cell death, autophagy-dependent cell death, alkaliptosis, oxeiptosis, and immunogenic cell death (ICD)), and immune cell infiltration 18-21. Gene set variation analysis (GSVA) was performed to find the most significant pathways between different groups, according to the gene sets “c2.cp.kegg.v2022.1.Hs.symbols.gmt” and “h.all.v2022.1.Hs.symbols.gmt” downloaded from the MsigDB database (https://www.gsea-msigdb.org/gsea/msigdb/) 22. GSEA was conducted with with R package “org.Hs.eg.db” “clusterProfiler” “enrichplot” and “DOSE/limma” to investigate the enrichment of Gene Ontology (GO) and Kyoto Encyclopedia of Gene and Genome (KEGG) pathways between high and low-risk group. Gene sets with p < 0.05 were regarded as significant enrichment. GO and KEGG functional enrichment analyses of the list genes were performed with “clusterProfiler” and “org.Hs.eg.db” packages and visualized with “enrichplot” and “ggplot2” packages.

### Consensus clustering and principal component analysis

R package “ConsensusClusterPlus” was adopted for consensus clustering analysis to classify GC patients from meta-cohort into distinct subgroups. The k-means algorithm was applied to choose the optimal number of groups. PCA was carried out with “ggplot2” and “Rtsne” packages to evaluate the distribution of patients in different clusters.

### Construction of glycogen riskScore model

The DEGs between each two of the three clusters (identified from the above consensus clustering analysis) were obtained using the Wilcoxon rank-sum test by setting a threshold of adjusted P < 1E-8. The intersection genes of the three comparisons were identified for further analyses. Univariate Cox regression was performed with “survival” package to acquire significant genes correlated with OS in the meta-cohort from the intersection genes. LASSO Cox regression analysis was additionally conducted to avoid overfitting between signatures with “glmnet” package. With the expression levels of the identified genes and regression coefficients, the risk score for each patient could be calculated by the formula: riskScore*_i_*=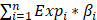, where n was the number of the identified genes, *Exp_i_* was the expression level of the gene, and *β_i_* was the corresponding regression coefficient. According to the median risk score, GC patients in meta-cohort were separate into low- or high-risk groups.

### Prognosis analysis

Kaplan-Meier (K-M) curves with log-rank test and the forest plots assessed by the univariate or multivariate Cox proportional hazards were performed to compare the significance of differences in survival time between GC patients with different features, using 'survival' and 'survminer' packages. The package “timeROC” was applied to generate time-dependent receiver operating characteristic (ROC) curves and calculate area under the curve (AUC) values to evaluate the specificity and sensitivity of the risk score. The correlations of the risk score and different clinical parameters including age, gender and stage were explored by the chi-square test or Fisher's exact test. And all the heatmaps were presented by “pheatmap” package. A predictive nomogram was additionally constructed by integrating different clinical characteristics with the risk score using the “rms” package. The association between different glycogen metabolism levels, glycogen clusters, risk score, stage and OS status was evaluated and visualized by the “GGalluvial” and “ggplot2” packages.

### Comparison of prognostic value with other reported signatures

To further estimate the prognostic value of the glycogen riskScore model, ten other GC signatures derived from the 10 latest published studies were identified, including Liu's glycolysis signature 23, Jiang's early-stage signature 24, Han's cuprotosis signature 25, Sang's TME signature 26, Zhou's oxidative stress related gene (OSRG) signature 27, Xu's apoptosis signature 28, Zhang's chromobox (CBX) signature 29, He's chemokine and chemokine receptor (CCR) signature 30, Mak's cancer-associated fibroblast (CAF) signature 31, and Li's necroptosis-related gene (NRG) signature 32. To ensure consistency and comparability, the risk score for each patient was calculated using the same Cox regression analysis method as employed for the glycogen riskScore model. This approach allowed us to directly compare the predictive capabilities of these various models on a uniform scale. The prognostic value of each model was firstly evaluated using K-M curves complemented by log-rank tests. This step was designed to compare survival outcomes between low- and high-risk groups defined by each signature, providing insights into the prognostic capability of each model. Further, we employed time-dependent ROC analysis, using median risk score as cutoff values, to assess the predictive accuracy of each signature model over time. This analysis is crucial for understanding how well each model distinguishes between different risk groups at various time points. Additionally, the concordance index (C-index) for each model was calculated using the R package “survcomp”. The C-index could provide a quantitative assessment of each model's prediction accuracy and reliability, measuring the concordance between predicted and observed survival outcomes. Restricted mean survival (RMS) time curve was analyzed and plotted with “survival” and “ggplot2” packages. RMS could offer an estimate of the average survival time within a specified time frame, aiding in understanding the OS benefit associated with each signature.

### Gene correlation analysis and protein-protein interaction network

Pearson's correlation analysis was performed to explore the associations among the genes included in the glycogen riskScore model. The correlation network was visualized among genes with |r| > 0.4 by using “reshape2” and “igraph” packages. A protein-protein interaction (PPI) network of the genes in the glycogen riskScore model was built with a confidence level of 0.15 via the STRING database (version 11.5, https://string-db.org/) 33. Cytoscape software (version 3.6.1) was used to visualize the network 34.

### Prediction of chemotherapy and immunotherapy response

To explore the predictive value of the glycogen riskScore model for chemotherapy in GC patients, we performed a ridge regression based on the gene expression profiles of meta-cohort and Genomics of Drug Sensitivity in Cancer cell lines. The half-maximal inhibitory concentration (IC50) of eight common chemotherapy related agents including axitinib, cisplatin, docetaxel, doxorubicin, gemcitabine, metformin, paclitaxel, rapamycin, and sorafenib were calculated with “pRRophetic” package 35. Using the CellMiner database, the relationship between the glycogen riskScore and sensitivity of 216 antitumor drugs was also explored in the National Cancer Institute (NCI) 60 cancer cell lines from nine different malignancies 36.

The immunophenoscore (IPS), scored as z scores by integration of four immunogenicity-related cell types (effector cells, immunosuppressive cells, MHC molecules, and immunomodulators), is significantly related with response to immunotherapy regimens 37. Tumor Immune Dysfunction and Exclusion (TIDE, http://tide.dfci.harvard.edu) is an advanced bioinformatics method to predict immune checkpoint inhibitor (ICI) responses based on transcriptome profile 38. IPS and TIDE were utilized to evaluate the association of glycogen riskScore model and immunotherapy responses. And the results were presented by “ggpubr” package.

### Assessment of tumor mutation burden, TME, and immune checkpoint correlation

The mutation landscape of glycogen metabolism genes and the top altered genes in low- and high-risk groups was displayed by waterfall plots. Difference of mutation rate of the top altered genes in low- and high-risk groups were analyzed using Wilcoxon test. TMB (tumor mutation burden) score, refers to mutations (Mut) per one million coding bases (Mb), was calculated for each sample from TCGA cohort with “maftools” package 39. Cytolytic activity (CYT) score and predicted neoantigen data for GC patients were retrieved from Rooney's study to evaluate intergroup difference 40. The immune infiltration of GC patients was estimated with XCELL, TIMER, QUANTISEQ, MCPCOUNTER, EPIC, CIBERSORT and CIBERSORT-ABS algorithms. These diverse algorithms allowed for a comprehensive assessment of the immune landscape in the TME. R package “estimate” was applied to calculate the stromal and immune score of each patient, providing a measure of the immune and stromal components of the tumor. The stemness indexes of DNA methylation-based stemness scores (DNAss) and mRNA expression-based stemness scores (RNAss) were obtained from the previous study, which helped in understanding the stem-like characteristics of the tumor 41. The correlation of above factors and glycogen riskScore model was performed with spearman's correlation test and visualized with “ggplot2” package. In addition, the relationship of common immune checkpoint genes, m6A regulators and cuproptosis genes with glycogen riskScore was also explored with spearman's correlation test.

### Validation of riskScore model in pan-cancer

The risk score of each patient with 33 types of cancers from TCGA datasets was calculated with the same method as that in GC. The K-M method with log-rank test was applied to generate and compare the difference of survival curves for patients with each type of cancer in low- and high-risk groups with median as cutoff value. R packages “survival” and “forestplot” were used to present the results of univariate analysis of riskScore in pan-cancer using the Cox proportional hazards regression model. Microsatellite instability (MSI) status and neoantigen data of pan-cancer were retrieved from The Cancer Immunome Atlas (TCIA, https://tcia.at/home). The correlation of riskScore with TMB, MSI and neoantigen was analyzed with spearman's correlation test and displayed as radar map with “fmsb” package. The relationship of riskScore with common checkpoint genes was also evaluated by pearson's correlation analysis and showed with “Reshape2” and “RColorBrewer” packages.

### Cell culture and siRNA transfection

The human GC cell line HGC-27 used in our study was obtained from the National Institute of Cells (Shanghai, China). The cancer cells were cultivated in RPMI‑1640 medium with 10% fetal calf serum (Gibco; USA) and maintained at 37˚C in 5% carbon dioxide. The siRNAs targeting CDS1, LARP6 and TUBB6 with si-NC were synthesized by Generay (Shanghai, China). The sequences were: si-CDS1#1: 5′-AAU AUC UGU UUC UUU GUC GCU-3′, si-CDS1#2: 5′-AUA GAU GAU CAG GAA AAA CAA-3′; si-LARP6#1: 5′-UCC AAC UCG UCC ACG UCC U-3′, si-LARP6#2: 5′-ACA AGC UGG GAU AUG UGA GCG UUA A-3′; si-TUBB6#1: 5′-UCU AUU CCG ACU AUC CAU CGA-3′, si-TUBB6#2: 5′-UGA CUA AUU ACA UGA CUU GGC-3′; si-NC: 5'-UUC UCC GAA CGU GUC ACG UTT-3'. Lipofectamine 2000 (Thermo Fisher Scientific, USA) was used to transfect the siRNAs and si-NC into HGC-27 cells.

### Quantitative Real-time Polymerase Chain Reaction (qRT-PCR)

Total RNA of HGC-27 cells was extracted with Trizol (Invitrogen, Carlsbad, CA, USA) in accordance with the manufacturer's protocol and further assessed using the ultraviolet spectrophotometer. Reverse transcription and qRT-PCR reactions were conducted with PrimeScript™ RT reagent Kit (Takara, Kyoto, Japan) and SYBR® Premix Ex Taq II (Tli RNaseH Plus) (Takara, Kyoto, Japan) according to the instructions, respectively. Amplification and detection of mRNAs were performed on LightCycler® 480 Real-Time PCR System (Roche Diagnostics, Mannheim, Germany). The 2^-ΔΔCT^ method with GAPDH as an internal control was applied to evaluate the quantification of mRNAs. The gene primer sequences were synthesized by Generay (Shanghai, China): CDS1: 5′-AAA CCG AGA GCA CCA GCG ACA A-3′ (forward) and 5′-GGG TTC TAT CTG AGG ATG GTG G-3′ (reverse); LARP6: 5′-TGA AAA CCT GGA GAA GGA CGC C-3′ (forward) and 5′-ATG TGC TGT GGT TCT CCA GTC C-3′ (reverse); TUBB6: 5′-TGG ACT TAG AGC CAG GCA CCA T-3′ (forward) and 5′-TTT CGC CCA GTT GTT CCC TGC A-3′ (reverse); GAPDH: 5′-GTC TCC TCT GAC TTC AAC AGC G-3′ (forward) and 5′-ACC ACC CTG TTG CTG TAG CCA A-3′ (reverse).

### Cell viability assay

Proliferation rate of HGC-27 cells after transfection was assessed using CCK-8 assay kit (Abcam, Shanghai, China). A density of 2×10^3^ cells/well were grown in 96-well plates. After 1, 2, 3, 4, and 5 days of culture, 10 μl CCK-8 reagent was added per well. After incubation for 2 h at 37°C, absorbance value of each well at 450 nm was evaluated on a microplate spectrophotometer (Thermo Fisher, USA).

### Cell apoptosis assay

Apoptosis status of HGC-27 cells after transfection was measured using Annexin V-FITC/PI Apoptosis Detection Kit (Keygen Biotech, Nanjing, China) on a CytoFLEX Flow Cytometer (Becton Dickinson) according to the manufacturer's instructions. All results were then analyzed by Flowjo software (version 10.8.1).

### Transwell migration and invasion assay

Migration and invasion ability of HGC-27 cells after transfection were evaluated with a transwell chamber system (12µm pore size, Millipore, Bedford, MA, USA). For migration assay, twenty-four hours after transfection of siRNAs or si-NC, 1×10^5^ cells were seeded onto the upper chambers. For invasion assay, 1×10^5^ cells were plated pre-coated with 250 µg/mL Matrigel (BD, Bioscience, Pharmingen) on the upper layer of the chambers. In both assays, the cells were grown in culture medium supplemented with 2% serum and the chambers were fixed into 24-well plates with a complete medium. After incubation for 48h, invaded cells on the lower chamber were fixed with 4% methanol and stained with 0.1% crystalline violet. Three random fields for stained cells were captured and analyzed under a microscope.

### Statistical analysis

R platform v4.1.0 was used for statistics analyses. Wilcoxon test was conducted to compare factors in different groups. Chi square test or Fisher's exact test was applied to assess the difference of clinical phenotypes in different groups. Pearson's or spearman's correlation test was performed to evaluate the relationship of glycogen riskScore with different features. K-M curves with log-rank test were used to compare the survival differences. Univariate and multivariate Cox regression analyses were adopted to identify the independent prognostic factors for patients. All tests were two-tailed and a P < 0.05 or specifically indicated was considered as statistically significant.

## Results

### Glycogen metabolism: potential prognostic biomarker and immune regulator in GC

**Figure 1:**
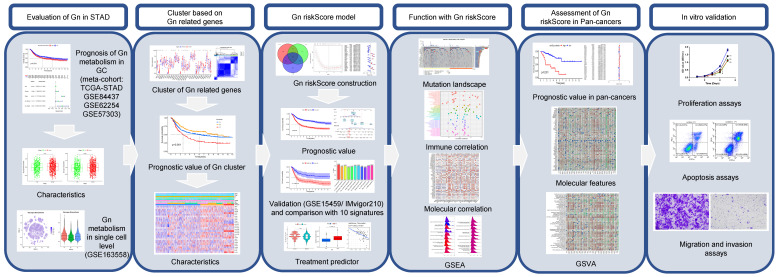
The flowchart of our study.

**Figure 2:**
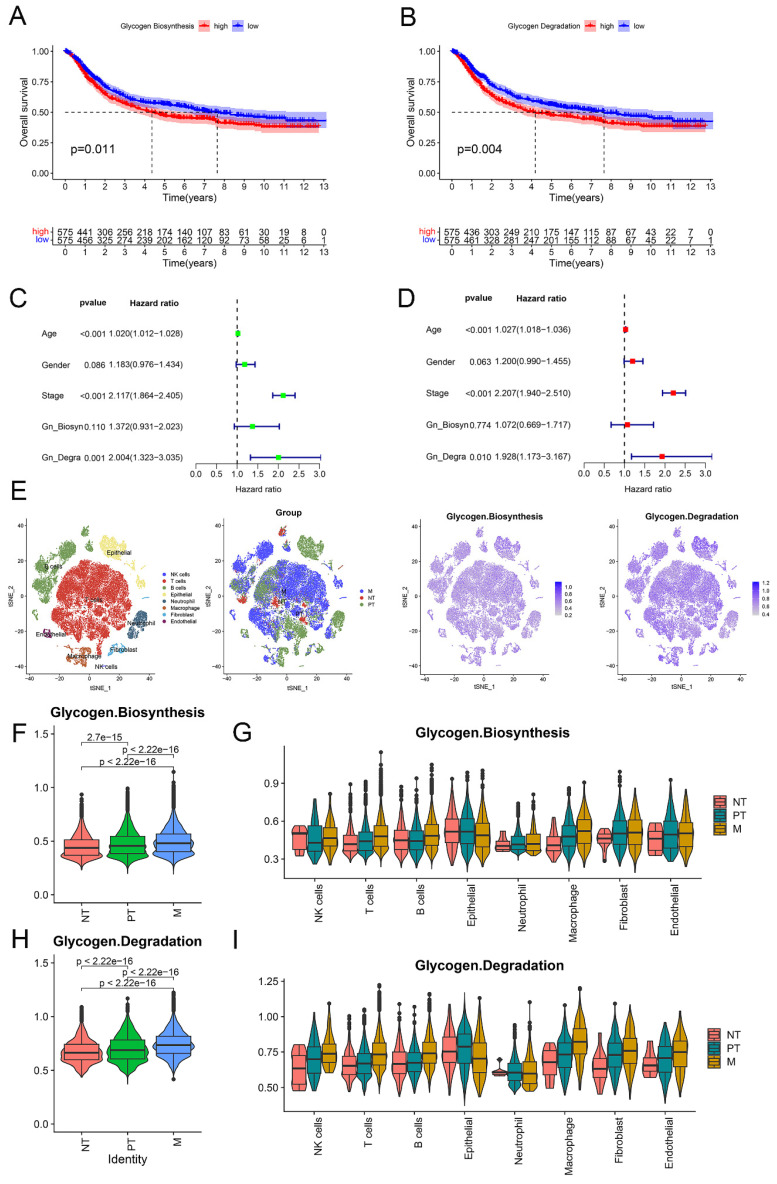
Assessment of glycogen metabolism in gastric cancer (GC). Kaplan-Meier (K-M) curve for overall survival (OS) of GC patients with low and high level of glycogen biosynthesis (A) and glycogen degradation (B) in meta-cohort. Univariate (C) and multivariate (D) Cox regression analyses evaluated the prognostic value of glycogen biosynthesis and degradation levels. E: Single-cell RNA (scRNA) analyses showing levels of glycogen biosynthesis and degradation in different cell types of tumor microenvironment (TME) with t-distributed stochastic neighbor embedding (tSNE) maps. F-I: Violin plots revealed distribution of glycogen biosynthesis (F & G) and degradation (H & I) in cells of TME. NT: adjacent non-tumor tissue; PT: primary tumor; M: metastatic samples.

As the flowchart (Figure 1) shows, we firstly evaluated the role of glycogen metabolism including glycogen biosynthesis and degradation in GC meta-cohort. K-M curves showed that higher levels of both glycogen biosynthesis (P = 0.011) and degradation (P = 0.004) were significantly related with worse OS in GC patients (Figure 2A & B). Univariate Cox regression analyses showed that higher level of glycogen degradation (P = 0.001) was significantly associated with poorer OS, while glycogen biosynthesis (P = 0.11) with borderline significance. Additional multivariate Cox regression revealed that glycogen degradation (P = 0.01) could act as an independent indicator for predicting OS in GC (Figure 2C & D). Glycogen metabolism's connection to clinical traits among GC patients revealed no distinctions in normal vs. tumor tissue or by age, gender, or TNM stage, except for glycogen degradation, which was higher in stage III-IV patients (P = 0.027, Figure S1).

To establish glycogen metabolism's role in GC's TME cells, we analyzed scRNA-seq data from 10 samples of 6 GC patients, including 3 primary tumor, 1 adjacent non-tumoral, and 6 metastatic samples. tSNE maps showed widespread enrichment of glycogen biosynthesis and degradation across diverse cell types (Figure 2E). Violin plots distinctly showed the lowest levels of glycogen metabolism in non-tumoral samples, while the highest levels were observed in metastatic samples, suggesting an association between increased glycogen metabolism and the progression of GC. Immune cell type specific analysis confirmed consistent glycogen patterns across various immune cell subsets (Figure 2F-I), highlighting its potential role in modulating immune responses in the context of GC.

### Dysregulation and prognostic roles of glycogen metabolism genes in GC

Expression levels of 31 glycogen metabolism genes (7 in biosynthesis, 13 in degradation, and 11 in both) were evaluated in normal and tumor tissues from the TCGA cohort. Around 58% (18 of 31) of genes showed significant upregulation, while 4 (GCK, PPP1R3C, G6PC, and PYGM) were downregulated in tumors (Figure S2A). Copy number variation (CNV) frequency analysis indicated PPP1CA had the highest increase, and PPP1R3C had the highest deletion frequency, aligning with their expression trends (Figure S2B). The locations of CNV alterations on the chromosome are shown in Figure S2C. Genetic mutations of glycogen metabolism genes were found in about a quarter of GC samples (24.94%). PPP1R3A had the highest mutation rate (6%), mainly as missense mutations (Figure S2D).

**Figure 3:**
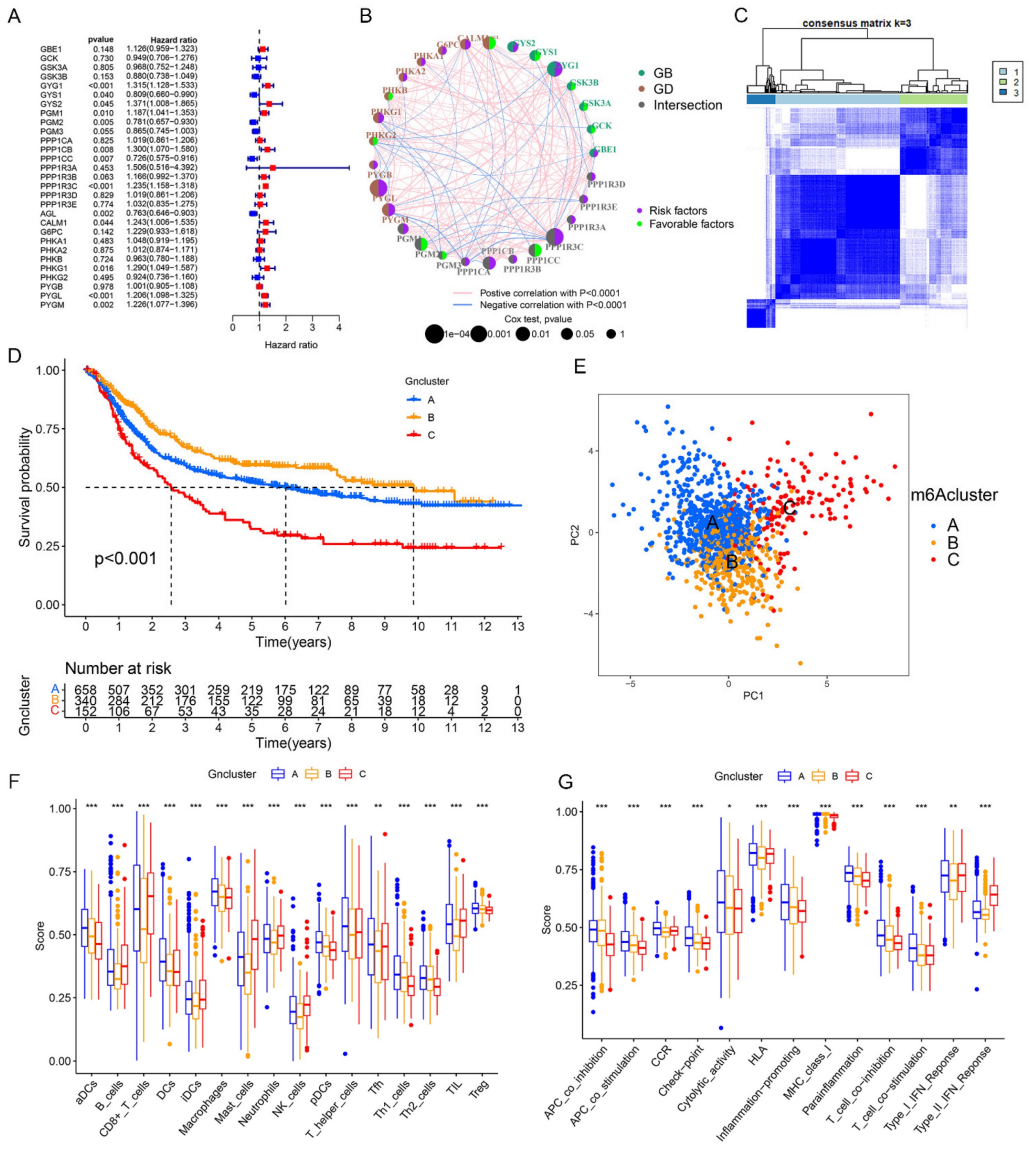
Consensus clustering of gastric cancer (GC) patients based on glycogen metabolism genes. A: Univariate Cox regression analysis of glycogen metabolism genes in GC patients of meta-cohort. B: Prognostic network of glycogen metabolism genes. C: Consensus clustering matrix of GC patients in meta-cohort with best cluster number of three. D: Kaplan-Meier (K-M) curve for overall survival (OS) of GC patients in the three clusters. E: Principal component analysis (PCA) analysis of GC patients in the three clusters. Differences of immune cell infiltration (F) and function (G) in the three clusters. (*P < 0.05; **P < 0.01; ***P < 0.001).

Among 29 identified glycogen metabolism genes in the meta-cohort, higher expression levels of CALM1, GYG1, GBE1, PYGM, PYGL, PYGB, PHKG1, PPP1R3C, PPP1R3B, PPP1R3A, PPP1CB, PPP1CA, and PGM1 were correlated with worse overall survival OS in STAD patients. Conversely, AGL, GYS1, GSK3B, PHKG2, PPP1CC, PGM3, and PGM2 were linked to favorable OS (P < 0.05, Figure S3). Univariate Cox regression analysis indicated that elevated GYG1, GYS2, PGM1, PPP1CB, PPP1R3C, CALM1, PHKG1, PYGM, and PYGL were unfavorable OS predictors, while higher GYS1, PGM2, PPP1CC, and AGL levels were associated with better OS (P < 0.05, Figure 3A). Prognostic network maps displayed prevalent positive relationships among glycogen metabolism genes, suggesting their synergistic effect in GC (Figure 3B).

### Generation and characteristics of GC subgroups based on consensus clustering of glycogen metabolism genes

To better understand the potential roles of glycogen metabolism in carcinogenesis and progression of GC, we performed consensus clustering analysis on 1150 GC patients from meta-cohort using 29 glycogen metabolism genes (excluding CALM2 and CALM3, which were not identified in the meta-cohort). Based on the expression patterns and patients' distribution, we determined that k = 3 clusters yielded the greatest differentiation (Cluster A: 658 patients; Cluster B: 340 patients; Cluster C: 152 patients; Figure 3C). K-M curves illustrated that Cluster C exhibited the poorest OS, while Cluster B showed the most favorable outcome (Figure 3D). Principal component analysis (PCA) confirmed distinct patient distribution among the clusters (Figure 3E). Exploration of immune cell infiltration and immune functions across the three groups revealed significant differences. Cluster C exhibited the highest levels, while Cluster B displayed the lowest, in immune cells (B cells, CD8 T cells, mast cells, neutrophils, natural killer cells, tumor infiltrating lymphocytes) and type II interferon (IFN) response (Figure 3F & G). Furthermore, GSVA enrichment analysis unveiled distinct mechanisms among the clusters. Cluster C was associated with immune-inhibitory or cancer-promoting pathways like TGF beta signaling, adherens junction, mTOR signaling, gap junction, and WNT signaling pathway. In contrast, Cluster B showed connections with gene repair pathways such as nucleotide excision repair, mismatch repair, and base excision repair (Figure S4 & Table S1).

### Construction, validation and comparison of glycogen riskScore model in GC

**Figure 4:**
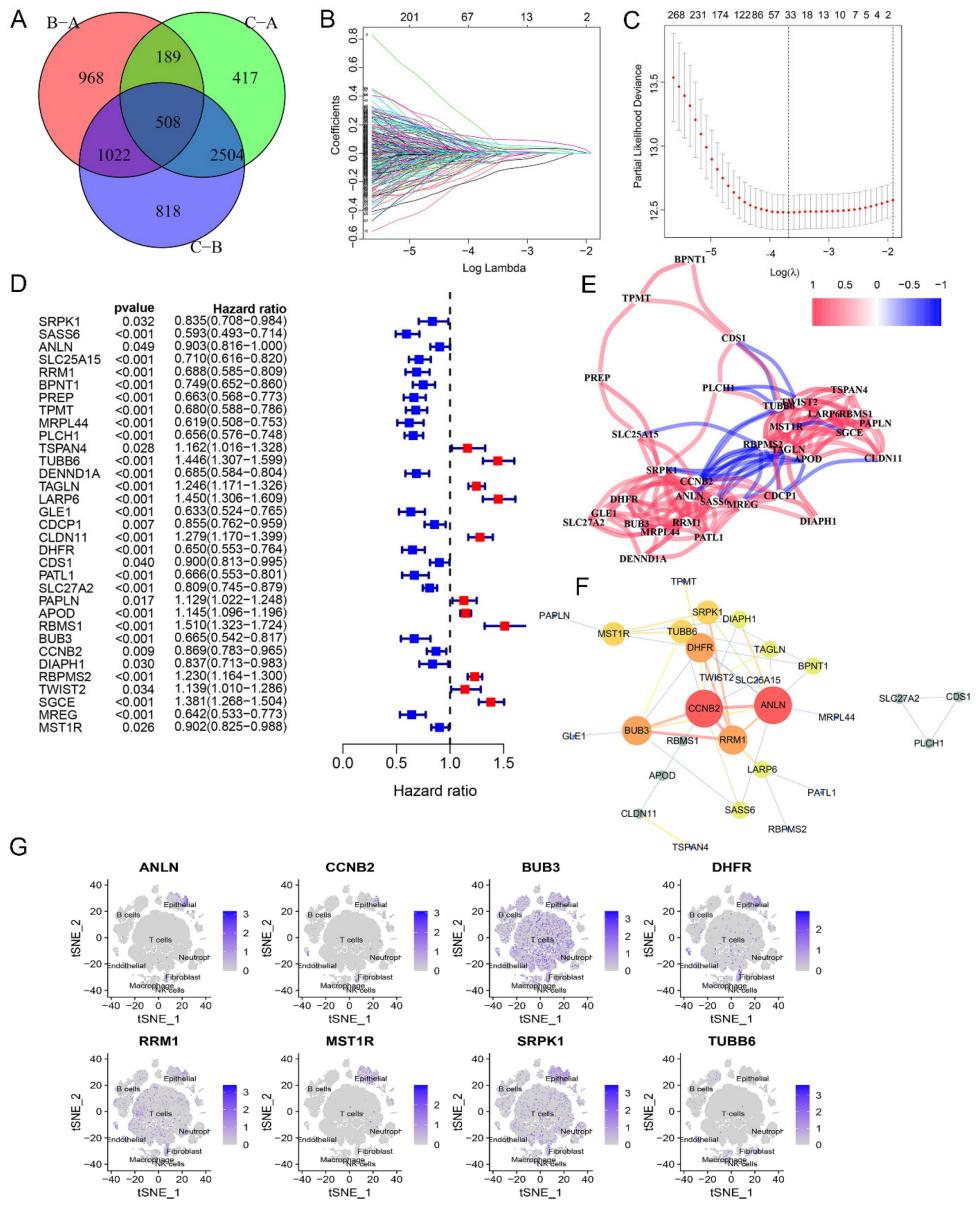
Generation of glycogen riskScore model. A: Venn diagram of the differently expressed genes among the three clusters. B & C: Least absolute shrinkage and selection operator (LASSO) regression analysis with minimum lambda value to build the prognostic model. D: Univariate Cox regression analysis of 33 genes enrolled in the glycogen riskScore model. E: Correlation networks of the 33 genes included in the glycogen riskScore model. F: Protein-protein interaction (PPI) networks of the 33 genes in the glycogen riskScore model. G: Distribution of the eight hub genes among the 33 genes in tumor microenvironment (TME) cells with t-distributed stochastic neighbor embedding (tSNE) feature plots.

Next, a differential gene expression analysis was performed to identify differentially expressed glycogen metabolism related genes among three different clusters. A total of 508 genes showed significant changes in all three comparisons (Figure 4A). KEGG and GO enrichment analysis linked these genes to cancer related pathways like cell cycle, p53 signaling, DNA replication, and mismatch repair (Figure S5). With univariate Cox regression followed by LASSO analysis in meta-cohort, we identified 33 genes for constructing the glycogen riskScore model (Figure 4B-C). Among them, there were 11 risk factors and 22 protective molecules (Figure 4D). The correlation network of these 33 genes revealed mainly positive relationships (Figure 4E). Notably, risk factors tended to have negative relationships with protective indicators. A protein-protein interaction (PPI) network identified 8 hub genes (ANLN, CCNB2, BUB3, DHFR, RRM1, MST1R, SRPK1, TUBB6) with the highest confidence interaction scores (Figure 4E & F). The majority of the genes (except for TUBB6) were expressed in epithelial. BUB3, DHFR, RRM1, and SRPK1 were also expressed in immune cells of GC's tumor microenvironment (Figure 4G).

**Figure 5:**
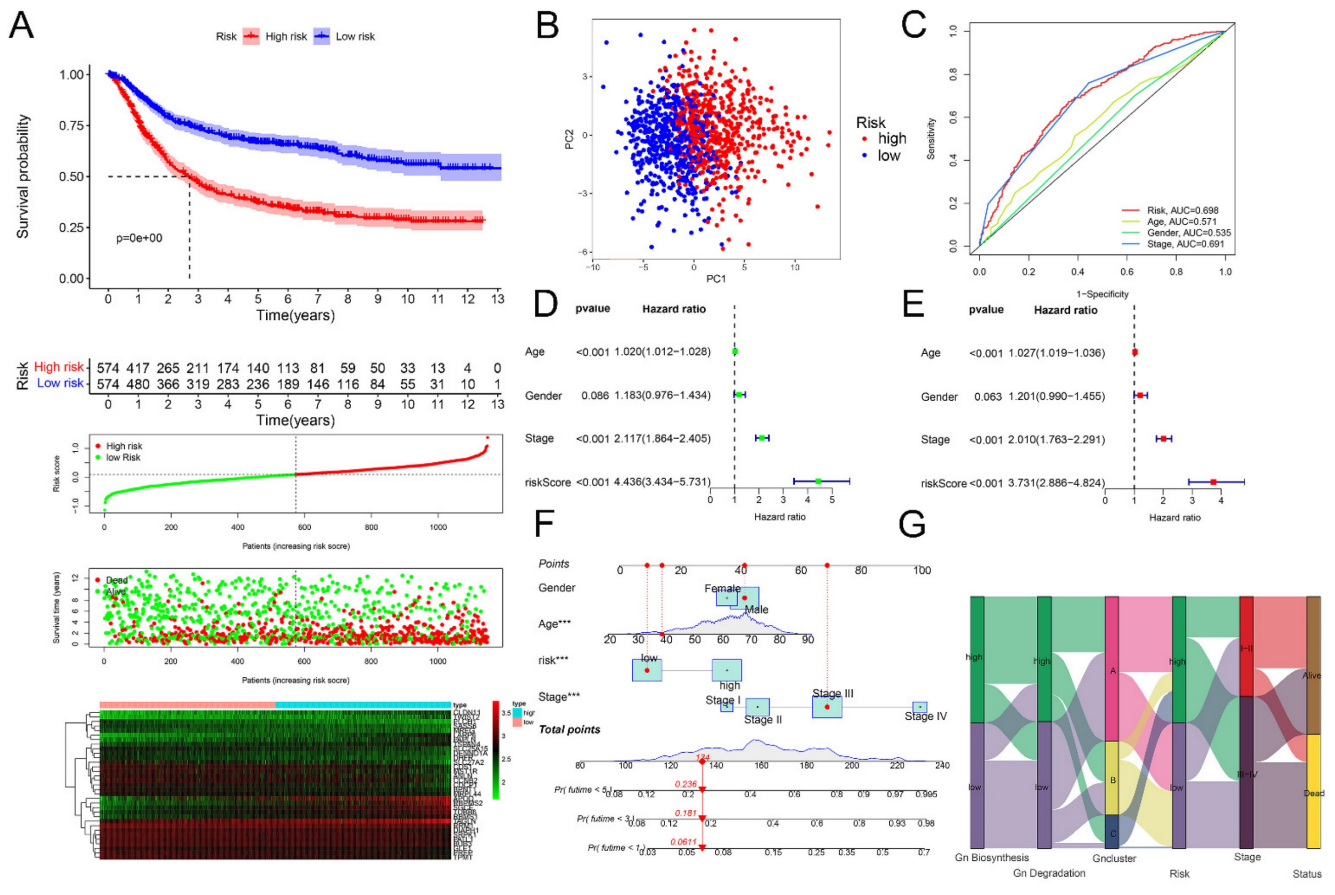
Evaluation of glycogen riskScore model in prognosis of gastric cancer (GC). A: Kaplan-Meier (K-M) curve (top 1) with distribution of risk score (top 2), overall survival (OS) status (top 3) and heatmap (bottom) of the 33 genes according to median of glycogen riskScore in meta-cohort. B: Principal component analysis (PCA) analysis of GC patients in low- and high-risk group. C: Receiver operating characteristic (ROC) curve for glycogen riskScore and clinical features at 5 years. Univariate (D) and multivariate (E) Cox regression analyses of glycogen riskScore and clinical characteristics. F: Nomogram combining glycogen riskScore and other clinical parameters. G: Sankey plot for distribution of GC patients with different glycogen metabolism levels, glycogen clusters, glycogen riskScore, stage and surviving status.

Then, we calculated the glycogen riskScore for each of the 1150 GC patients, categorizing them into low- and high-risk groups based on median riskScore. High-risk patients showed significantly worse OS (P < 0.001), visually indicated by K-M curves, ranked dot map and scatter plot. The expression levels of the 33 glycogen metabolism-related genes were also displayed as a heatmap (Figure 5A). PCA also confirmed the distribution of patients with different risks (Figure 5B). The glycogen riskScore model outperformed individual clinical factors, predicting 5-year OS with an AUC of 0.698 (Figure 5C). The glycogen riskScore was associated with clinical characteristics. Higher scores were observed in patients with younger age, advanced T, N, M and later stage (Figure S6). Univariate and multivariate Cox regression affirmed its independent prognostic value (Figure 5D & E). A nomogram integrating clinical factors and riskScore predicted 1-, 3-, and 5-year OS better than single factors (Figure 5F). Visualizing GC patients across glycogen metabolism levels, clusters, riskScore, stage, and survival status showed high metabolism linked to cluster C, high riskScore, advanced stage, and elevated death risk (Figure 5G).

**Figure 6:**
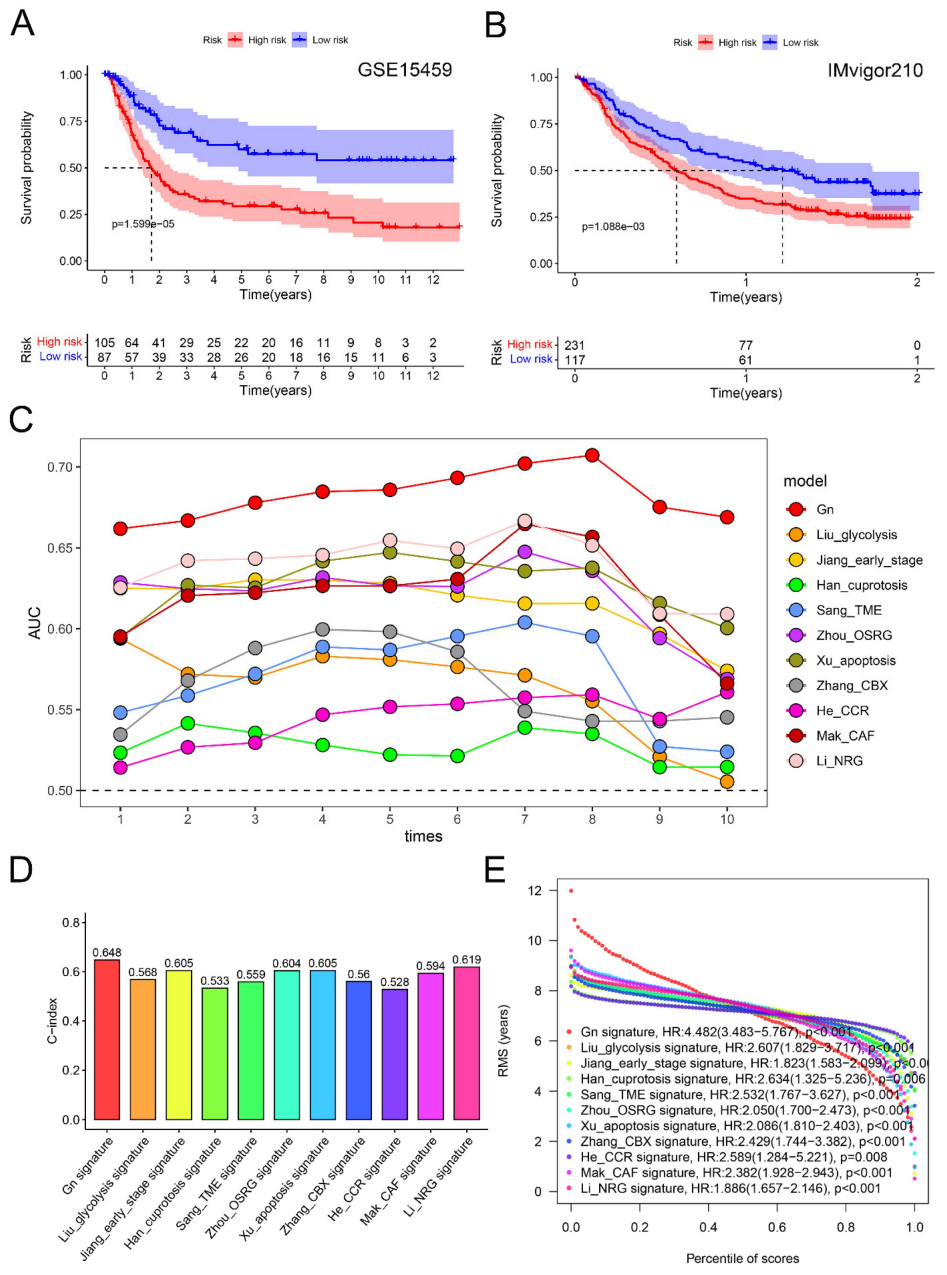
Verification and comparison of glycogen riskScore signature. A: Kaplan-Meier (K-M) curve for overall survival (OS) of gastric cancer (GC) patients in GSE15459 cohort. B: K-M curve for OS of bladder urothelial carcinoma (BLCA) patients received atezolizumab therapy in IMvigor210 cohort. C: Comparison of area under the curve (AUC) for assessing predictive accuracy of glycogen riskScore signature and the other ten latest published signatures for OS from 1 to 10 years. D: Comparison of C-index of glycogen riskScore signature with the other ten latest published signatures for OS. E: Restricted mean survival (RMS) time curve of glycogen riskScore and the other ten latest published signatures. Gn: glycogen riskScore; TME: tumor microenvironment; OSRG: oxidative stress related gene; CBX: chromobox; CCR: chemokine and chemokine receptor; CAF: cancer-associated fibroblast; NRG: necroptosis-related gene.

To validate the reliability of the glycogen riskScore model for GC prognosis, an external cohort (GSE15459) with 192 GC patients was enrolled. Consistent with the results of meta-cohort, the high-risk group exhibited significantly worse OS in the external cohort (P < 0.001, Figure 6A). Furthermore, the prognostic value of the riskScore model was examined in the immunotherapeutic IMvigor210 cohort. Similarly, higher riskScore predicted worse OS in BLCA patients undergoing anti-PD-L1 antibody (atezolizumab) therapy (P < 0.001, Figure 6B).

Further, we compared the glycogen riskScore signature with ten latest published gene signatures for predictive efficacy of prognosis in GC 23-32. Using the same method, we calculated risk scores for each signature in the meta-cohort. Notably, all ten signatures demonstrated a significant correlation with poorer OS, except for He's CCR signature, which showed borderline significance (P = 0.052; Figure S7). Time-dependent ROC curves provided a temporal assessment of each signature's predictive accuracy. Impressively, our glycogen riskScore signature consistently outperformed the others, achieving the highest AUC values at any year (Figure 6C; ROC curves at 1, 3, and 5 years were depicted in Figure S7). This superiority in AUCs at multiple time points underscores the robustness of our signature in predicting patient outcomes over a range of time horizons. When assessing the C-index, our glycogen riskScore model excelled with the highest value of 0.648 (Figure 6D). This highest C-index value reinforces the reliability of our model in correctly predicting patient survival, setting it apart from the other assessed signatures. Furthermore, the analysis of the RMS time curve offered insights into the long-term prediction efficacy of our model. Notably, the glycogen riskScore signature showed superior predictive power beyond 8 years (Figure 6E). This extended prediction efficacy indicates the potential long-term clinical utility of our model in managing GC patients.

### Indicative role of glycogen riskScore model for immunotherapy and chemotherapy response

**Figure 7:**
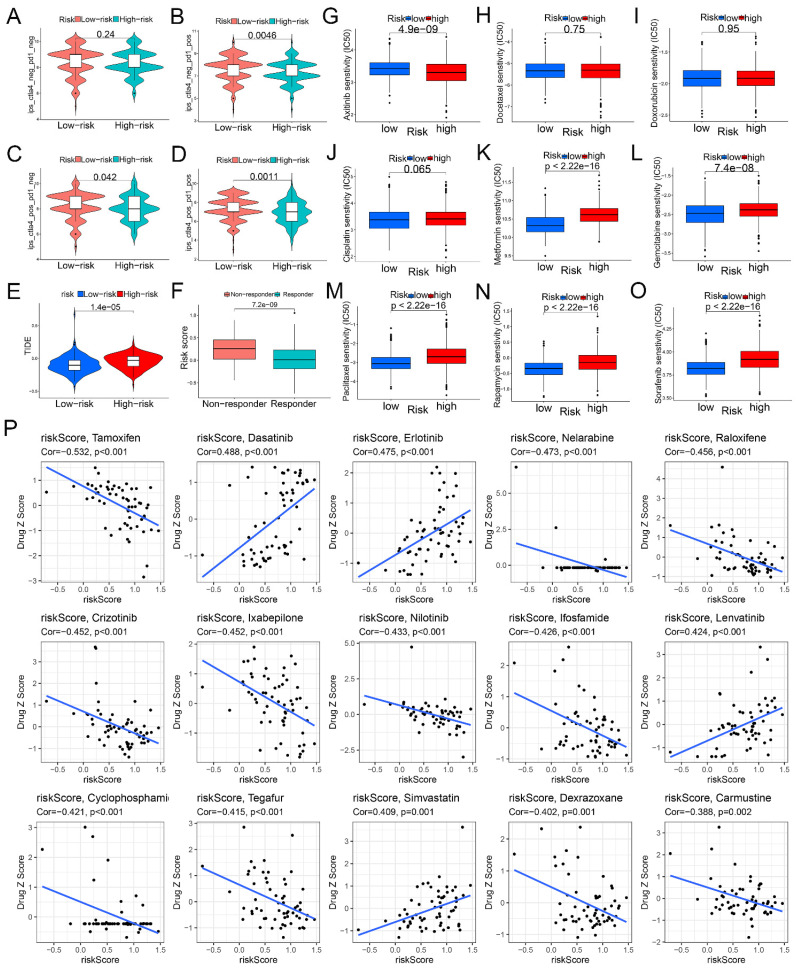
Correlation of glycogen riskScore with treatment response. A-D: Different immunophenoscore (IPS) score in gastric cancer (GC) patients with low- and high-risk groups. E: Difference of Tumor Immune Dysfunction and Exclusion (TIDE) score between low- and high-risk groups. F: Comparison of glycogen riskScore in immunotherapy responders and non-responders. G-O: Distribution of half-maximal inhibitory concentration (IC50) value of axitinib (G), docetaxel (H), doxorubicin (I), cisplatin (J), metformin (K), gemcitabine (L), paclitaxel (M), rapamycin (N), and sorafenib (O). P: Correlation of glycogen riskScore and sensitivity of the top 15 anti-tumor drugs in the Cell Miner database.

We then evaluated the potential role of the glycogen riskScore model in predicting treatment responses, focusing on immunotherapy, chemotherapy, and targeted therapy. Low-risk patients showed higher sensitivity to immune checkpoint inhibitor (ICI) immunotherapy, indicated by significantly higher immunophenoscores (IPS) for ctla4_neg_pd1_pos, ctla4_pos_pd1_neg, and ctla4_pos_pd1_pos signatures (P < 0.05, Figure 7A-D). Similarly, Tumor Immune Dysfunction and Exclusion (TIDE) analysis revealed higher scores in the high-risk group, suggesting increased immune escape and resistance to immunotherapy (P < 0.001, Figure 7E). And responders to immunotherapy exhibited lower glycogen riskScore (P < 0.001, Figure 7F).

Next, we analyzed chemotherapy response by predicting the half-maximal inhibitory concentration (IC50) values for 9 common chemotherapeutic and targeted agents. The results showed that no difference of IC50 values between the two groups for docetaxel, doxorubicin and cisplatin. High-risk patients were more sensitive to axitinib (lower IC50), while low-risk patients were sensitive to metformin, gemcitabine, paclitaxel, rapamycin, and sorafenib (Figure 7G-O). We also evaluated the glycogen riskScore signature's relationship with anti-tumor drug sensitivity in the National Cancer Institute (NCI) 60 cell lines. Among 56 significant drugs (Table S2), the top 15 (Figure 7P) revealed tamoxifen, nelarabine, raloxifene, crizotinib, ixabepilone, nilotinib, ifosfamide, cyclophosphamide, tegafur, dexrazoxane, and carmustine negatively correlated with the riskScore, while dasatinib, erlotinib, lenvatinib, and simvastatin showed a positive correlation.

### Characterizing mutation, immune and functional features in patients stratified by glycogen riskScore

**Figure 8:**
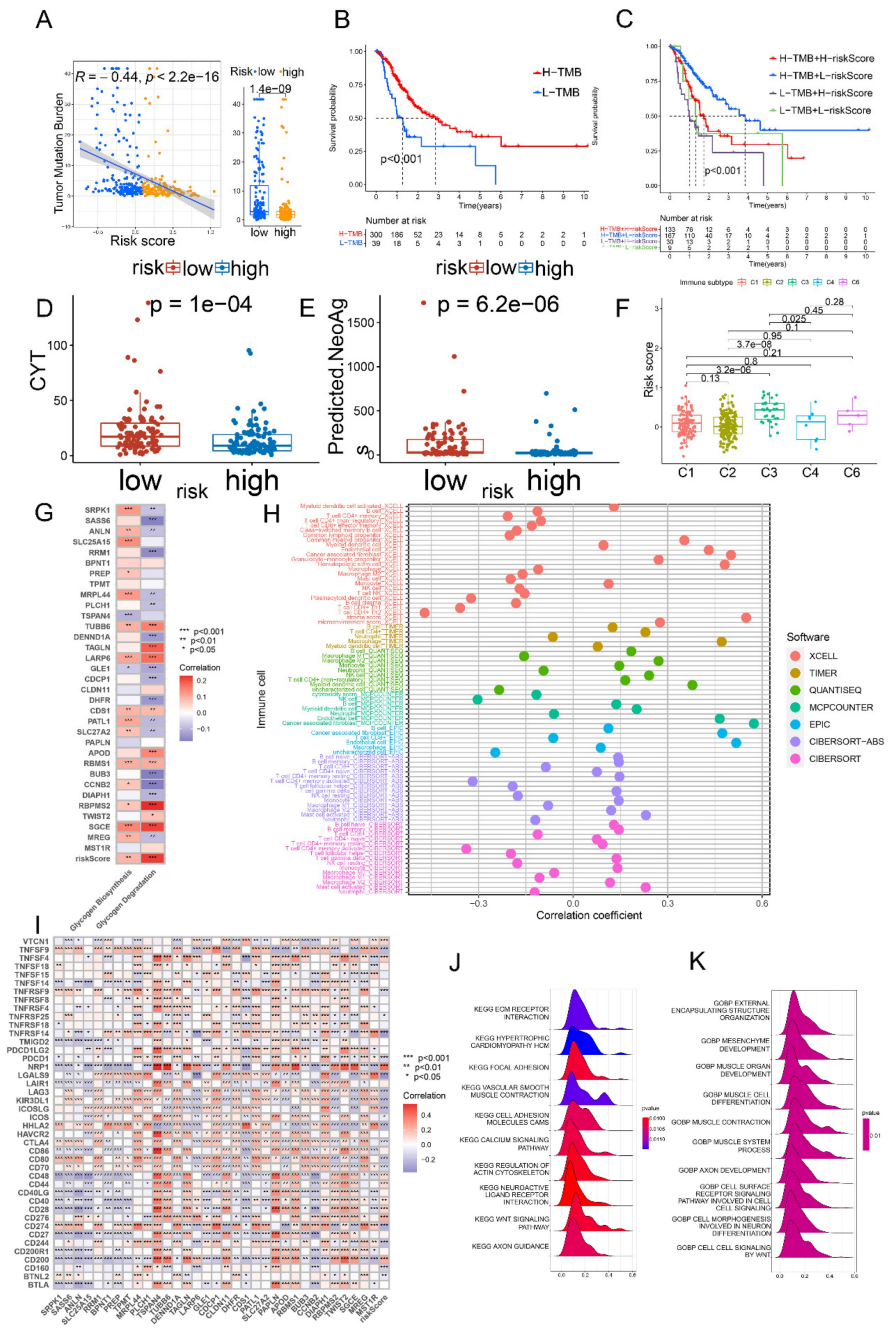
Genetic and immune correlation of glycogen riskScore in gastric cancer (GC) patients. A: Correlation of tumor mutation burden (TMB) score and glycogen riskScore. B: Kaplan-Meier (K-M) curve of GC patients with low and high TMB score in The Cancer Genome Atlas (TCGA) cohort. C: K-M curve of GC patients based on combination of TMB score and glycogen riskScore. D: Different levels of cytolytic activity (CYT) in GC patients with low and high glycogen riskScore. E: Different levels of predicted neoantigens in GC patients with low and high glycogen riskScore. F: Distribution of glycogen riskScore in GC patients with different immune subtypes from TCGA cohort. G: Relationship of glycogen riskScore model and levels of glycogen biosynthesis and glycogen degradation. H: Correlation between glycogen riskScore and immune cells calculated with 7 algorithms. I: Correlation of glycogen riskScore with common immune checkpoint genes. J-K: Gene set enrichment analysis (GSEA) concerning Kyoto Encyclopedia of Gene and Genome (KEGG; J) and Gene Ontology (GO; K) enrichment analyses based on glycogen riskScore. (*P < 0.05; **P < 0.01; ***P < 0.001).

To delve into the genomic distinctions between the low- and high-risk groups classified by glycogen riskScore, the study explored somatic mutation landscapes in each group. Utilizing comprehensive genomic data from the TCGA cohort, we generated the waterfall plot to illustrate the mutation frequencies and patterns in each group. The plot revealed a striking difference in the mutation rates between the low and high-risk groups, with the low-risk GC patients exhibiting a higher rate of genetic mutations. Notable differences were observed in genes like TTN, MUC16, LRP1B, ARID1A, SYNE1, FLG, FAT4, and so on (Figure S8A). Furthermore, our analysis revealed a significant negative correlation between the glycogen riskScore and TMB score among the GC patients in the TCGA cohort. Specifically, patients in the low-risk group displayed higher TMB levels (P < 0.001, Figure 8A). Higher TMB was associated with longer OS. Thus, by combining TMB and glycogen riskScore, patients with high TMB and low riskScore demonstrated the most favorable outcome (Figure 8B & C).

The study further examined immune microenvironment differences between low- and high-risk groups. Intergroup different analysis revealed that low-risk GC patients had higher cytolytic activity (CYT) scores and more predicted neoantigens (Figure 8D & E). For programmed cell death (PCD) patterns, 11 of 14 levels were significantly higher in the low-risk group (Figure S8B). Considering immune subtypes, the C3 (inflammatory) subtype had the highest glycogen riskScore (Figure 8F). Using ESTIMATE algorithms, glycogen riskScore positively correlated with immune and stromal scores (Figure S8C & D). Correlation analysis of glycogen riskScore and immune cells based on 7 algorithms showed that cancer-associated fibroblast (CAF), M2 macrophage, myeloid dendritic cell, activated mast cell, monocyte, and resting memory CD4^+^ T cell positively correlated with the riskScore. Conversely, M1 macrophage, activated memory CD4^+^ T cell, and CD8^+^ T cell exhibited negative correlations (Figure 8H). Tumor stemness, measured by DNA methylation-based (DNAss) and mRNA expression-based (RNAss) scores, negatively correlated with glycogen riskScore (Figure S8E & F).

We then examined the relationship of the glycogen riskScore model and glycogen metabolism, finding positive correlations with both glycogen biosynthesis and degradation levels. Among the 33 genes for model construction, TUBB6, LARP6, CDS1, RBMS1, RBPMS2, and SGCE were simultaneously positively related with both glycogen biosynthesis and degradation, while GLE1 exhibited negative relationship (Figure 8G). The associations of glycogen riskScore with common immune checkpoint genes, m6A regulators and cuproptosis genes were further evaluated. Negative relationships were found between the riskScore and immunostimulators CD70, CD80, ICOS, ICOSLG, TNFRSF14, TNFRSF25, TNFRSF9 and TNFSF9, while positive correlation with immunoinhibitor VTCN1 (Figure 8I). Meanwhile, glycogen riskScore was also significantly related with m6A regulators and cuproptosis genes (Figure S8G & H). Functional analysis using GSEA demonstrated close relationship of the riskScore with cancer and immune system, such as KEGG terms of WNT signaling pathway and TGF beta signaling pathway, GO terms of cell signaling by WNT and surface receptor signaling pathway (Figure 8J & K; Table S3).

### Pan-cancer analysis of glycogen riskScore model

**Figure 9:**
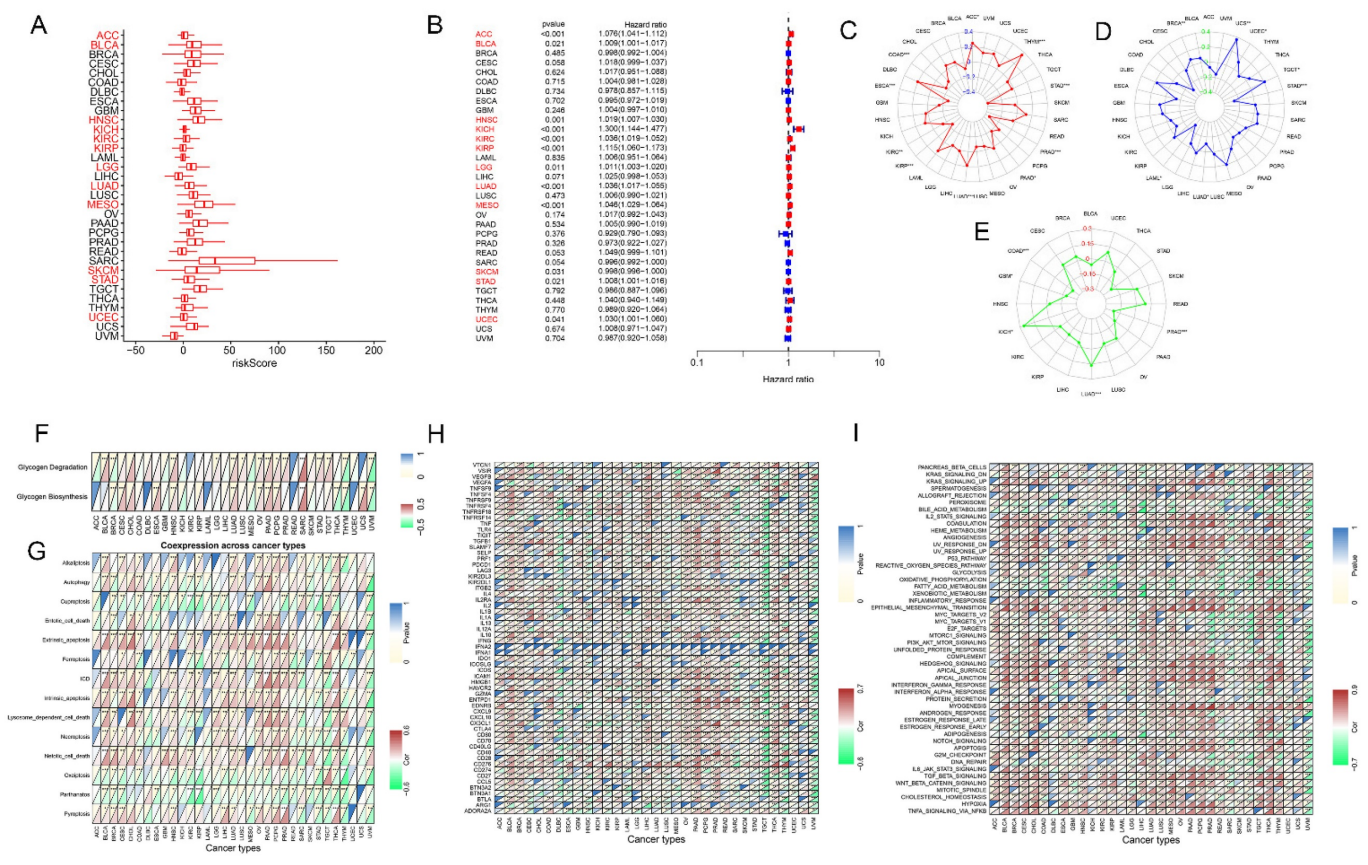
Confirmation of glycogen riskScore model in The Cancer Genome Atlas (TCGA) pan-cancer cohort. A-B: Distribution (A) and univariate Cox regression analysis (B) of riskScore in pan-cancer (red indicates statistical significance in Cox regression analysis). C-E: Radar maps for association of glycogen riskScore and tumor mutation burden (TMB; C), microsatellite instability (MSI; D) and neoantigen (E). F: Correlation of glycogen riskScore and levels of glycogen biosynthesis and glycogen degradation in pan-cancer. G: Association of glycogen riskScore with 14 programmed cell death (PCD) patterns in pan-cancer. H: Relationship of glycogen riskScore with common immune checkpoint genes in pan-cancer. I: Association of glycogen riskScore with hallmark pathways in pan-cancer. (*P < 0.05; **P < 0.01; ***P < 0.001).

To assess the applicability of glycogen riskScore in diverse types of tumors, TCGA pan-cancer cohort was analyzed. The riskScore of patients in each type of cancer was calculated with the same method as that in GC (Figure 9A). K-M curves revealed that high riskScore could predict worse OS in 20 types of cancers including adrenocortical carcinoma (ACC), bladder urothelial carcinoma (BLCA), cervical squamous cell carcinoma and endocervical adenocarcinoma (CESC), glioblastoma multiforme (GBM), head and neck squamous cell carcinoma (HNSC), kidney chromophobe (KICH), kidney renal clear cell carcinoma (KIRC), kidney renal papillary cell carcinoma (KIRP), brain lower grade glioma (LGG), liver hepatocellular carcinoma (LIHC), lung adenocarcinoma (LUAD), lung squamous cell carcinoma (LUSC), mesothelioma (MESO), ovarian serous cystadenocarcinoma (OV), pancreatic adenocarcinoma (PAAD), rectum adenocarcinoma (READ), STAD, thyroid carcinoma (THCA), uterine corpus endometrial carcinoma (UCEC) and uterine carcinosarcoma (UCS), while favorable outcome in breast invasive carcinoma (BRCA), cholangiocarcinoma (CHOL), sarcoma (SARC) and skin cutaneous melanoma (SKCM) (P < 0.05, Figure S9). Univariate Cox regression analyses showed that high riskScore associated with poorer OS in ACC, BLCA, HNSC, KICH, KIRC, KIRP, LGG, LUAD, MESO, STAD, and UCEC; and correlated with better outcomes in SKCM (P < 0.05, Figure 9B). Distinct glycogen riskScore distributions were found in relation to age (BLCA, BRCA, esophageal carcinoma (ESCA), KIRP, LIHC, LUAD, PAAD, and STAD), gender (ESCA, KIRC, LIHC, LUSC, SARC, and thymoma (THYM)), and stage (ACC, BLCA, colon adenocarcinoma (COAD), KICH, KIRC, KIRP, LIHC, LUAD, testicular germ cell tumors (TGCT), and THCA). Besides, compared with patients with complete response (CR) + partial response (PR) after first course of treatment, higher glycogen riskScore was assessed in patients with stable disease (SD) + progressive disease (PD) in ACC, HNSC, KICH, KIRC, LUAD and UCEC (Figure S10).

Then, the association of glycogen riskScore with TMB, MSI and neoantigen were examined across pan-cancer. The results showed that the glycogen signature was negatively correlated with TMB score in STAD, PRAD, ESCA, KIRP, PAAD, COAD and KIRC, while positive relationships were showed in LUAD, ACC and THYM. For MSI, negative correlation with riskScore was shown in acute myeloid leukemia (LAML), STAD and LUAD, while positive correlation in BRCA, UCEC, TGCT and UCS. In addition, glycogen riskScore was negatively related to neoantigen in COAD, PRAD and GBM, while positively associated in LUAD and KICH (Figure 9C-E).

Subsequently, correlations of the glycogen riskScore model and glycogen metabolism levels across pan-cancer were assessed. Significantly positive correlation of glycogen riskScore with glycogen biosynthesis could be found in 9 types of tumors, with glycogen degradation in 12 types of cancers (Figure 9F). To further explore the effect of the glycogen riskScore on TME of pan-cancer, associations of glycogen riskScore with PCD patterns, common checkpoint genes and hallmark pathways in pan-cancer were explored. Negative correlation of the glycogen riskScore with most PCD patterns was identified in SKCM, STAD, THYM, and uveal melanoma (UVM). Three PCD patterns (cuproptosis, oxeiptosis, and parthanatos) were almost negatively associated with the riskScore in pan-cancer (Figure 9G). And the glycogen riskScore was significantly positively related with immunoinhibitors TGFB1, ICAM1 and CD276, and showed significantly negative relationship with immunostimulators TNFSF14 across pan-cancer (Figure 9H). Moreover, the glycogen riskScore signature was significantly positively related with IL2-STAT5 signaling, coagulation, angiogenesis, inflammatory response, epithelial mesenchymal transition, myogenesis, NOTCH signaling, TGF beta signaling, WNT beta catenin signaling and hypoxia across pan-cancer, which are important pathways that occur in TME to promote development and progression of cancer. Meanwhile, peroxisome and oxidative phosphorylation pathways exhibited negative relationship with glycogen riskScore in pan-cancer (Figure 9I).

### Glycogen metabolism related genes promote progression of GC

**Figure 10:**
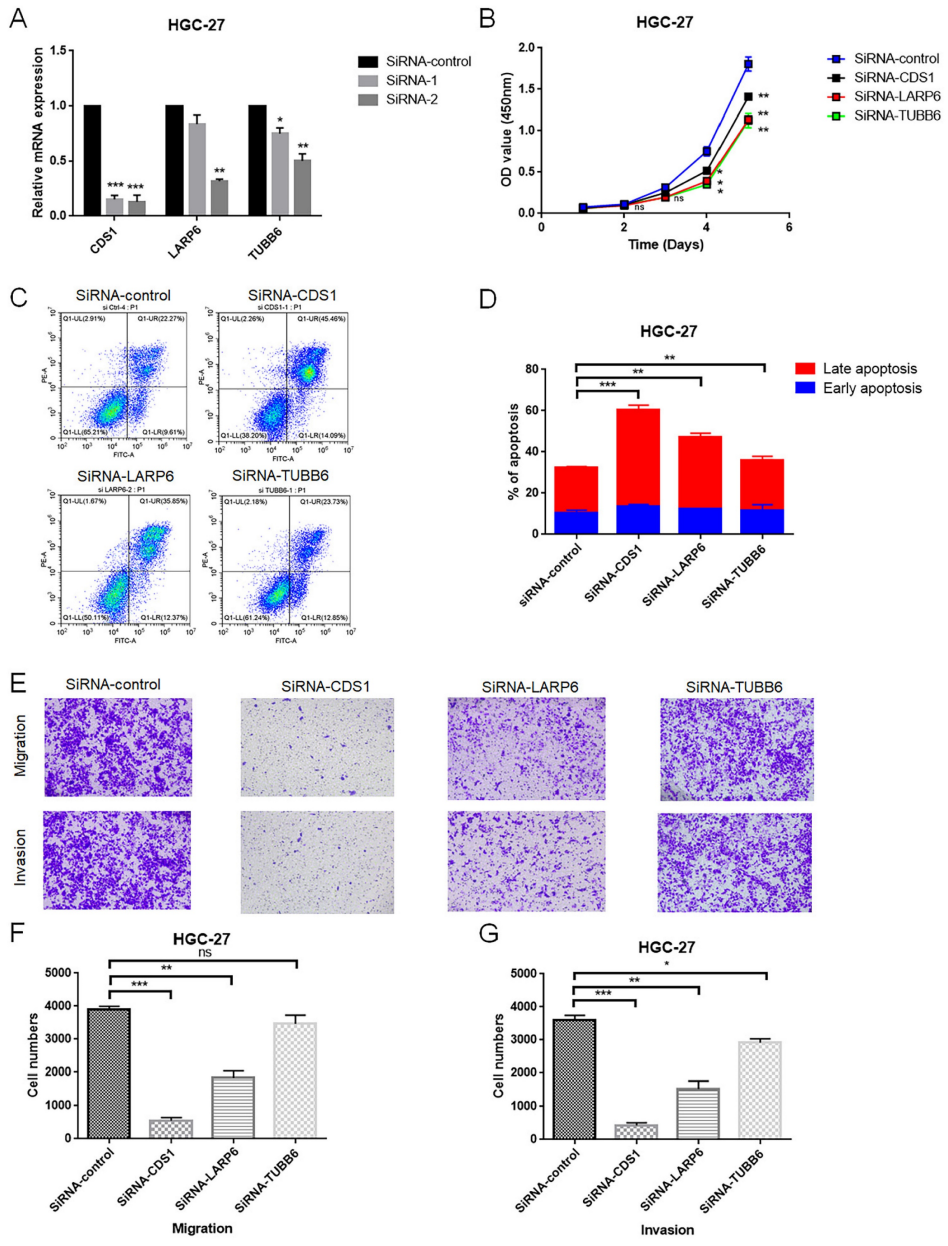
*In vitro* validation of the role of LARP6, CDS1 and TUBB6 in HGC-27 cell line. A: The efficiency of siRNAs for LARP6, CDS1 and TUBB6 was assessed utilizing quantitative real-time polymerase chain reaction (qRT-PCR). B: The viability of gastric cancer (GC) cells was evaluated by CCK-8 assay after transfection of siRNA or normal control (NC). C-D: Cell apoptosis after transfection of siRNA or NC was tested using flow cytometry. E-G: Transwell assays were applied to detect the invasion and migration ability of GC cells after transfection of siRNA or NC. All experiments were repeated three times. (ns: non significance; *P < 0.05; **P < 0.01; ***P < 0.001).

To further assess the function of the glycogen metabolism related genes included in the riskScore model, we performed *in vitro* experiments. Glycogen metabolism related genes actively involving in glycogen metabolism were selected for further experimental verification, specifically those showed consistently positive relation patterns with both glycogen biosynthesis and degradation (Figure 8G). Among them, LARP6, CDS1 and TUBB6 were randomly selected to evaluate their effects on proliferation, apoptosis, invasion and metastasis of GC cells. Two siRNAs were designed for knock down the expression of the three target genes, respectively. As indicated by qRT-PCR, the siRNA-2 for each gene showed higher silencing efficiency than siRNA-1, and was selected for further experiment (Figure 10A). In HGC-27 cells, knockdown of LARP6, CDS1, and TUBB6 inhibited proliferation (Figure 10B). Apoptosis analysis revealed increased late apoptosis upon silencing these genes (Figure 10C & D). Moreover, CDS1 and LARP6 knockdown significantly reduced migration and invasion, while TUBB6 knockdown showed a weaker effect on invasion and no significant impact on migration in HGC-27 cells (Figure 10E-G).

## Discussion

Oncogenic metabolic reprogramming is a hallmark and central feature of cancer. Up-regulated glycogen metabolism levels along with the initiation and development of cancer could enable survival of tumor cells under adverse conditions and promote metastasis 14, 42-44. It has been suggested that glycogen metabolism might be a major energy source within TME and responsible for treatment effect of cancer patients. However, it is so far uninvestigated about glycogen metabolism in cancer including GC. In this study, we assessed glycogen metabolism level in GC using bulk and single-cell sequencing data, and developed a glycogen riskScore signature which could predict treatment response and clinical outcomes in GC patients. Pan-cancer analysis demonstrated its applicability across various tumor types. *In vitro* experiments further validated the significance of glycogen metabolism related genes in the progression of GC.

Initially, we utilized gene sets from Rosario's study 18 to calculate ssGSEA scores for assessing glycogen biosynthesis and degradation levels. K-M curves and Cox regression analyses revealed close relationship of glycogen metabolism, especially glycogen degradation, and clinical outcomes of GC patients. These findings demonstrated the importance of glycogen degradation, providing energy and metabolites, in driving malignant phenotype of GC compared to glycogen biosynthesis. This aligns with Terashima's study, which showed glycogen breakdown's role in GC cell survival 13. Exploration of glycogen metabolism at single-cell level revealed its prevalence across various cell types, with higher levels in tumor and metastatic cells, particularly in immune and stromal cells like T cells, macrophages, and endothelial cells. This emphasizes the significance of glycogen metabolism in TME of GC. K-M curves and univariate Cox regression analysis also revealed close link of glycogen metabolism genes with prognosis of GC patients. Based on consensus clustering of glycogen metabolism genes, GC patients in meta-cohort were classified into three clusters. Distinct clinical outcomes, biological pathways, TME characteristics, and infiltrating immune cells were observed among the three clusters.

Furthermore, univariate Cox regression and LASSO analysis of intersection genes from the three groups of comparisons across the three clusters were used to identify glycogen metabolism related genes to construct a glycogen metabolism riskScore model. Higher glycogen riskScore could act as an independent indicator for poorer OS, and outperformed other clinical characteristics, as evidenced by the highest AUC. An additional GC GSE15459 cohort and a BLCA immunotherapeutic cohort of IMvigor210 also confirmed the reliability of the riskScore signature. In addition, comparative analysis with the ten recently published gene signatures also revealed the superior prognostic performance of our glycogen riskScore model, making it a robust and effective tool for predicting GC prognosis.

Meanwhile, analyses with IPS and TIDE data demonstrated that lower glycogen riskScore corresponded to higher immunotherapy sensitivity, in line with IMvigor210 cohort findings. High TMB predicts better outcomes with ICI treatment across various cancers 45. In this study, high TMB was linked to improved survival in GC patients. Notably, glycogen riskScore showed a negative correlation with TMB score. Somatic mutation landscape indicated that low-risk GC patients had higher genetic mutation rates. Previous studies demonstrated that MUC16 and TTN mutations could predict high TMB and were related with better prognosis in pan-cancer including GC 46, 47. These results were consistent with our findings that MUC16 and TTN displayed higher mutation levels in the low-risk group. Interestingly, low glycogen riskScore was tied to higher tumor neoantigens, cytolytic activity, and most types of PCD patterns, likely contributing to enhanced anti-tumor immunity. Immune correlation analysis revealed its positive correlation with immune and stromal scores, predicting components within the tumor microenvironment. Pro-tumor immune cells, such as M2 macrophage and myeloid dendritic cell, correlated positively with riskScore, while anti-tumor immune cells like M1 macrophage showed a negative correlation 48. Correlation with immune checkpoint genes further supported the glycogen riskScore as a potential immunotherapy biomarker 49. On the other hand, the predictive value of the model could extend to chemotherapy and targeted therapy responses. Patients with low-risk scores might benefit from metformin, paclitaxel, rapamycin, and sorafenib, whereas high-risk patients could prioritize axitinib. Drug sensitivity analysis of NCI-60 cell lines also demonstrated specific drugs for the patients in the two groups. These results revealed the glycogen riskScore signature as an effective tool for guiding precise treatment strategies for GC.

Our study also conducted a comprehensive analysis of the glycogen riskScore in pan-cancer, affirming its prognostic significance. This riskScore demonstrated predictive power in 24 types of cancers, indicating poor outcomes in 20 types and favorable outcomes in 4. It was also associated with treatment response, showing higher scores in non-responders compared to patients achieving objective response in several cancer types. Furthermore, the riskScore correlated with important prognostic factors like TMB, MSI, and neoantigen levels in specific cancer categories 50, 51. It was worth mentioning that three types of PCD patterns (cuproptosis, oxeiptosis, and parthanatos) were negatively associated with the glycogen riskScore across pan-cancer, which was desirable to be further studied. The riskScore also showed close link to immune responses, favoring immunoinhibitory pathways and immunosuppressive cells while inhibiting immunostimulatory pathways and immune cells. It was deserved to be mentioned about the role of hypoxia in TME. As a central feature of TME, hypoxia has extensive effects on malignant phenotypes of cancer, including cancer stemness, angiogenesis, invasion and metastasis, metabolic reprogramming (including glycogen metabolism) and so on, which synergistically facilitate the development and progression of cancer 52. Our results demonstrating the close relationship between the glycogen riskScore and hypoxia pathway also verified the importance of the signature in predicting the status of hypoxic TME across pan-cancer. All these findings confirmed the reliable and valid value of the application of the glycogen riskScore model in pan-cancer.

Finally, we randomly selected three genes (LARP6, CDS1 and TUBB6) which showed consistently positive relationship with both glycogen biosynthesis and degradation to evaluate their effects on proliferation, apoptosis, invasion and metastasis of GC cells. Except for TUBB6 which did not significantly affect migration, all three genes demonstrated a clear role in promoting carcinogenesis in GC cells, further confirming the crucial role of glycogen metabolism in GC. Predictive value of LARP6 and TUBB6 for worse outcomes in GC patients could also be found in previous studies 53, 54. However, the exact roles and biological functions of these glycogen metabolism related genes are warranted for further assessment in future studies.

Some limitations of the study should also be listed. Firstly, though assessment and validation of glycogen metabolism and glycogen riskScore model with multi-omics data of TCGA and GEO datasets at bulk and single-cell levels through external validation of GC and BLCA immunotherapy cohorts followed by pan-cancer analyses, our study was conducted mainly based on public databases. Future prospective and multicenter clinical studies should be performed to facilitate the application of the signature with broad applicability in predicating prognosis and treatment response of cancer patients in the real world. Secondly, the glycogen metabolism genes and related genes identified in the study was proved to play vital roles in cancer and interactions with TME through in silico analysis and limited *in vitro* experiments. Thus, the exact mechanisms involving these genes need to be further explored with *in vitro* and *in vivo* experiments in the future.

## Conclusion

Due to the important roles of glycogen metabolism in GC, we constructed a glycogen riskScore model with multi-omics data, which could accurately classify the GC patients with different outcomes, genomic and immune landscape. The glycogen riskScore was proved to perform well in predicting clinical outcomes through the validation, immunotherapy and pan-cancer cohorts. In addition, the riskScore could predict response to chemotherapy and immunotherapy, thus helpful for patients to select optimal treatment strategies. Future studies confirmed our findings and explored the mechanisms of the identified genes, which are helpful for the application and understanding of this powerful biomarker in cancer patients.

## Supplementary Material

Supplementary figures and tables.

## References

[1] Russo AE, Strong VE (2019). Gastric Cancer Etiology and Management in Asia and the West. Annu Rev Med.

[2] Camargo MC, Figueiredo C, Machado JC (2019). Review: Gastric malignancies: Basic aspects. Helicobacter.

[3] Li K, Zhang A, Li X, Zhang H, Zhao L (2021). Advances in clinical immunotherapy for gastric cancer. Biochim Biophys Acta Rev Cancer.

[4] Warburg O (1956). On the origin of cancer cells. Science.

[5] Liberti MV, Locasale JW (2016). The Warburg Effect: How Does it Benefit Cancer Cells?. Trends Biochem Sci.

[6] Vander Heiden MG, Cantley LC, Thompson CB (2009). Understanding the Warburg effect: the metabolic requirements of cell proliferation. Science.

[7] Rousset M, Zweibaum A, Fogh J (1981). Presence of glycogen and growth-related variations in 58 cultured human tumor cell lines of various tissue origins. Cancer Res.

[8] Iida Y, Aoki K, Asakura T, Ueda K, Yanaihara N, Takakura S, Yamada K, Okamoto A, Tanaka T, Ohkawa K (2012). Hypoxia promotes glycogen synthesis and accumulation in human ovarian clear cell carcinoma. Int J Oncol.

[9] Maruggi M, Layng FI, Lemos R Jr, Garcia G, James BP, Sevilla M, Soldevilla F, Baaten BJ, de Jong PR, Koh MY, Powis G (2019). Absence of HIF1A Leads to Glycogen Accumulation and an Inflammatory Response That Enables Pancreatic Tumor Growth. Cancer Res.

[10] Ma R, Ji T, Zhang H, Dong W, Chen X, Xu P, Chen D, Liang X, Yin X, Liu Y, Ma J, Tang K, Zhang Y (2018). A Pck1-directed glycogen metabolic program regulates formation and maintenance of memory CD8(+) T cells. Nat Cell Biol.

[11] Adeva-Andany MM, Gonzalez-Lucan M, Donapetry-Garcia C, Fernandez-Fernandez C, Ameneiros-Rodriguez E (2016). Glycogen metabolism in humans. BBA Clin.

[12] Haase VH (2020). Got glycogen? An energy resource in HIF-mediated prevention of ischemic kidney injury. Kidney Int.

[13] Terashima M, Fujita Y, Togashi Y, Sakai K, De Velasco MA, Tomida S, Nishio K (2014). KIAA1199 interacts with glycogen phosphorylase kinase beta-subunit (PHKB) to promote glycogen breakdown and cancer cell survival. Oncotarget.

[14] Curtis M, Kenny HA, Ashcroft B, Mukherjee A, Johnson A, Zhang Y, Helou Y, Batlle R, Liu X, Gutierrez N, Gao X, Yamada SD, Lastra R (2019). Fibroblasts Mobilize Tumor Cell Glycogen to Promote Proliferation and Metastasis. Cell Metab.

[15] Necchi A, Joseph RW, Loriot Y, Hoffman-Censits J, Perez-Gracia JL, Petrylak DP, Derleth CL, Tayama D, Zhu Q, Ding B, Kaiser C, Rosenberg JE (2017). Atezolizumab in platinum-treated locally advanced or metastatic urothelial carcinoma: post-progression outcomes from the phase II IMvigor210 study. Ann Oncol.

[16] Jiang H, Yu D, Yang P, Guo R, Kong M, Gao Y, Yu X, Lu X, Fan X (2022). Revealing the transcriptional heterogeneity of organ-specific metastasis in human gastric cancer using single-cell RNA Sequencing. Clin Transl Med.

[17] Butler A, Hoffman P, Smibert P, Papalexi E, Satija R (2018). Integrating single-cell transcriptomic data across different conditions, technologies, and species. Nat Biotechnol.

[18] Rosario SR, Long MD, Affronti HC, Rowsam AM, Eng KH, Smiraglia DJ (2018). Pan-cancer analysis of transcriptional metabolic dysregulation using The Cancer Genome Atlas. Nat Commun.

[19] Coscia F, Lengyel E, Duraiswamy J, Ashcroft B, Bassani-Sternberg M, Wierer M, Johnson A, Wroblewski K, Montag A, Yamada SD, Lopez-Mendez B, Nilsson J, Mund A (2018). Multi-level Proteomics Identifies CT45 as a Chemosensitivity Mediator and Immunotherapy Target in Ovarian Cancer. Cell.

[20] Zou Y, Xie J, Zheng S, Liu W, Tang Y, Tian W, Deng X, Wu L, Zhang Y, Wong CW, Tan D, Liu Q, Xie X (2022). Leveraging diverse cell-death patterns to predict the prognosis and drug sensitivity of triple-negative breast cancer patients after surgery. Int J Surg.

[21] Fucikova J, Kepp O, Kasikova L, Petroni G, Yamazaki T, Liu P, Zhao L, Spisek R, Kroemer G, Galluzzi L (2020). Detection of immunogenic cell death and its relevance for cancer therapy. Cell Death Dis.

[22] Hanzelmann S, Castelo R, Guinney J (2013). GSVA: gene set variation analysis for microarray and RNA-seq data. BMC Bioinformatics.

[23] Liu Y, Wu M, Cao J, Zhu Y, Ma Y, Pu Y, Huo X, Wang J (2022). Identification and verification of a glycolysis-related gene signature for gastric cancer. Ann Transl Med.

[24] Jiang F, Lin H, Yan H, Sun X, Yang J, Dong M (2022). Construction of mRNA prognosis signature associated with differentially expressed genes in early stage of stomach adenocarcinomas based on TCGA and GEO datasets. Eur J Med Res.

[25] Han C, Zhang K, Mo X (2022). Construction of a Cuprotosis-Related Gene-Based Model to Improve the Prognostic Evaluation of Patients with Gastric Cancer. J Immunol Res.

[26] Sang Q, Dai W, Yu J, Chen Y, Fan Z, Liu J, Li F, Li J, Wu X, Hou J, Yu B, Feng H, Zhu ZG (2022). Identification of prognostic gene expression signatures based on the tumor microenvironment characterization of gastric cancer. Front Immunol.

[27] Zhou R, Peng N, Li W (2022). Constructing a novel gene signature derived from oxidative stress specific subtypes for predicting survival in stomach adenocarcinoma. Front Immunol.

[28] Xu C, Liu Z, Yan C, Xiao J (2022). Application of apoptosis-related genes in a multiomics-related prognostic model study of gastric cancer. Front Genet.

[29] Zhang YJ, Zhao LY, He X, Yao RF, Lu F, Lu BN, Pang ZR (2022). CBXs-related prognostic gene signature correlates with immune microenvironment in gastric cancer. Aging (Albany NY).

[30] He C, He L, Lu Q, Xiao J, Dong W (2022). The functions and prognostic values of chemokine and chemokine receptors in gastric cancer. Am J Cancer Res.

[31] Mak TK, Li X, Huang H, Wu K, Huang Z, He Y, Zhang C (2022). The cancer-associated fibroblast-related signature predicts prognosis and indicates immune microenvironment infiltration in gastric cancer. Front Immunol.

[32] Li Z, Yang W, Liu D, Ye W, Du G, Li X (2022). Construction of a novel signature and prediction of the immune landscape in gastric cancer based on necroptosis-related genes. Sci Rep.

[33] Szklarczyk D, Gable AL, Lyon D, Junge A, Wyder S, Huerta-Cepas J, Simonovic M, Doncheva NT, Morris JH, Bork P, Jensen LJ, Mering CV (2019). STRING v11: protein-protein association networks with increased coverage, supporting functional discovery in genome-wide experimental datasets. Nucleic Acids Res.

[34] Shannon P, Markiel A, Ozier O, Baliga NS, Wang JT, Ramage D, Amin N, Schwikowski B, Ideker T (2003). Cytoscape: a software environment for integrated models of biomolecular interaction networks. Genome Res.

[35] Geeleher P, Cox NJ, Huang RS (2014). Clinical drug response can be predicted using baseline gene expression levels and in vitro drug sensitivity in cell lines. Genome Biol.

[36] Reinhold WC, Sunshine M, Liu H, Varma S, Kohn KW, Morris J, Doroshow J, Pommier Y (2012). CellMiner: a web-based suite of genomic and pharmacologic tools to explore transcript and drug patterns in the NCI-60 cell line set. Cancer Res.

[37] Charoentong P, Finotello F, Angelova M, Mayer C, Efremova M, Rieder D, Hackl H, Trajanoski Z (2017). Pan-cancer Immunogenomic Analyses Reveal Genotype-Immunophenotype Relationships and Predictors of Response to Checkpoint Blockade. Cell Rep.

[38] Jiang P, Gu S, Pan D, Fu J, Sahu A, Hu X, Li Z, Traugh N, Bu X, Li B, Liu J, Freeman GJ, Brown MA (2018). Signatures of T cell dysfunction and exclusion predict cancer immunotherapy response. Nat Med.

[39] Schumacher TN, Kesmir C, van Buuren MM (2015). Biomarkers in cancer immunotherapy. Cancer Cell.

[40] Rooney MS, Shukla SA, Wu CJ, Getz G, Hacohen N (2015). Molecular and genetic properties of tumors associated with local immune cytolytic activity. Cell.

[41] Malta TM, Sokolov A, Gentles AJ, Burzykowski T, Poisson L, Weinstein JN, Kaminska B, Huelsken J, Omberg L, Gevaert O, Colaprico A, Czerwinska P, Mazurek S (2018). Machine Learning Identifies Stemness Features Associated with Oncogenic Dedifferentiation. Cell.

[42] Cheng KW, Agarwal R, Mitra S, Lee JS, Carey M, Gray JW, Mills GB (2012). Rab25 increases cellular ATP and glycogen stores protecting cancer cells from bioenergetic stress. EMBO Mol Med.

[43] Sato A, Kawasaki T, Kashiwaba M, Ishida K, Nagashima Y, Moritani S, Ichihara S, Sugai T (2015). Glycogen-rich clear cell carcinoma of the breast showing carcinomatous lymphangiosis and extremely aggressive clinical behavior. Pathol Int.

[44] Liu Q, Li J, Zhang W, Xiao C, Zhang S, Nian C, Li J, Su D, Chen L, Zhao Q, Shao H, Zhao H, Chen Q (2021). Glycogen accumulation and phase separation drives liver tumor initiation. Cell.

[45] Samstein RM, Lee CH, Shoushtari AN, Hellmann MD, Shen R, Janjigian YY, Barron DA, Zehir A, Jordan EJ, Omuro A, Kaley TJ, Kendall SM, Motzer RJ (2019). Tumor mutational load predicts survival after immunotherapy across multiple cancer types. Nat Genet.

[46] Li X, Pasche B, Zhang W, Chen K (2018). Association of MUC16 Mutation With Tumor Mutation Load and Outcomes in Patients With Gastric Cancer. JAMA Oncol.

[47] Yang Y, Zhang J, Chen Y, Xu R, Zhao Q, Guo W (2020). MUC4, MUC16, and TTN genes mutation correlated with prognosis, and predicted tumor mutation burden and immunotherapy efficacy in gastric cancer and pan-cancer. Clin Transl Med.

[48] Mao X, Xu J, Wang W, Liang C, Hua J, Liu J, Zhang B, Meng Q, Yu X, Shi S (2021). Crosstalk between cancer-associated fibroblasts and immune cells in the tumor microenvironment: new findings and future perspectives. Mol Cancer.

[49] Zhang Y, Zheng J (2020). Functions of Immune Checkpoint Molecules Beyond Immune Evasion. Adv Exp Med Biol.

[50] Baretti M, Le DT (2018). DNA mismatch repair in cancer. Pharmacol Ther.

[51] Xu P, Luo H, Kong Y, Lai WF, Cui L, Zhu X (2020). Cancer neoantigen: Boosting immunotherapy. Biomed Pharmacother.

[52] Ivan M, Fishel ML, Tudoran OM, Pollok KE, Wu X, Smith PJ (2022). Hypoxia signaling: Challenges and opportunities for cancer therapy. Semin Cancer Biol.

[53] Dai S, Huang Y, Liu T, Xu ZH, Liu T, Chen L, Wang ZW, Luo F (2021). Development and validation of RNA binding protein-applied prediction model for gastric cancer. Aging (Albany NY).

[54] Bai Y, Wei C, Zhong Y, Zhang Y, Long J, Huang S, Xie F, Tian Y, Wang X, Zhao H (2020). Development and Validation of a Prognostic Nomogram for Gastric Cancer Based on DNA Methylation-Driven Differentially Expressed Genes. Int J Biol Sci.

