# Supplementary material for: Integrated Multi-omics Data Analysis and In Vitro Validation Reveal the Crucial Role of Glycogen Metabolism in Gastric Cancer
Source: J Cancer. 2025 Jan 13;16(4):1243–63. doi: 10.7150/jca.104424 (PMC11786036; doi:10.7150/jca.104424)
Supplement: Supplementary file 1 — Supplementary figures and tables. [file jcav16p1243s1.pdf]

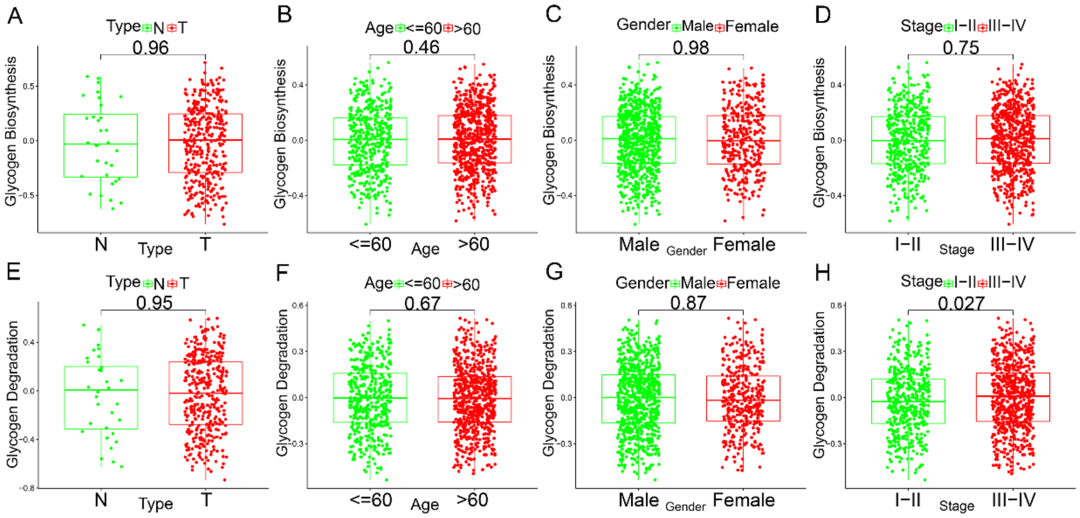

Figure S1: The association of glycogen biosynthesis (A-D) and degradation (E-H) levels and clinical characteristics (A&E: normal vs. tumor; B&F: age; C&G: gender; D&H: stage) of GC patients in meta-cohort. N: normal; T: tumor.

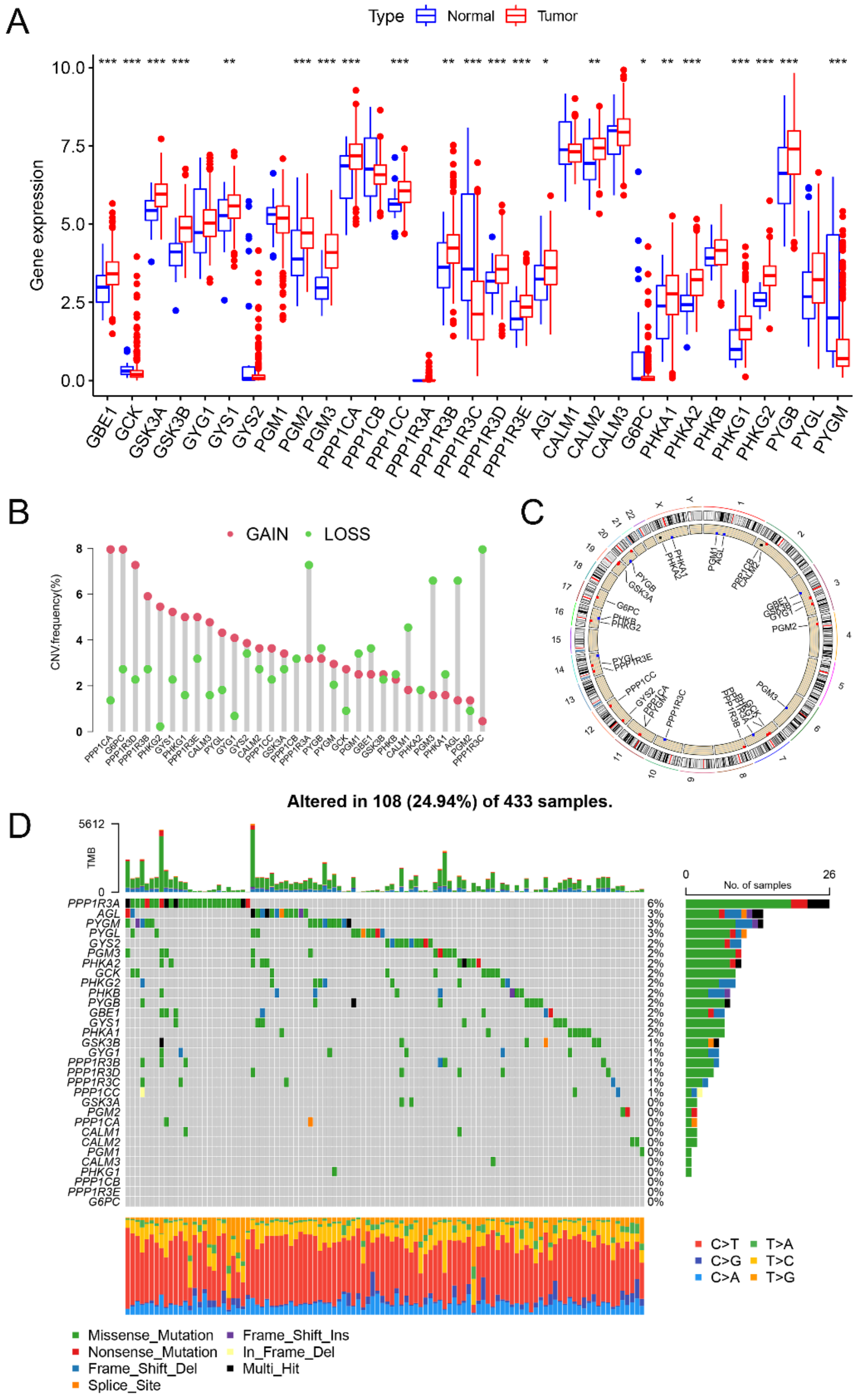

Figure S2: The Landscape of 31 glycogen metabolism genes in gastric cancer (GC). A: Differential expression of glycogen metabolism genes in normal and tumor of GC patients in The Cancer Genome Atlas (TCGA) cohort. B: Barplots showing Copy number variation (CNV) amplification (red dots) and CNV deletion (green dots) of the glycogen metabolism genes in TCGA cohort. C: The positions of CNV alteration of glycogen metabolism genes on chromosomes; Red dots: CNV gained; Green dots: CNV lost. D: Mutation status of glycogen metabolism genes of GC patients in TCGA cohort. (*P < 0.05; **P < 0.01; ***P < 0.001).

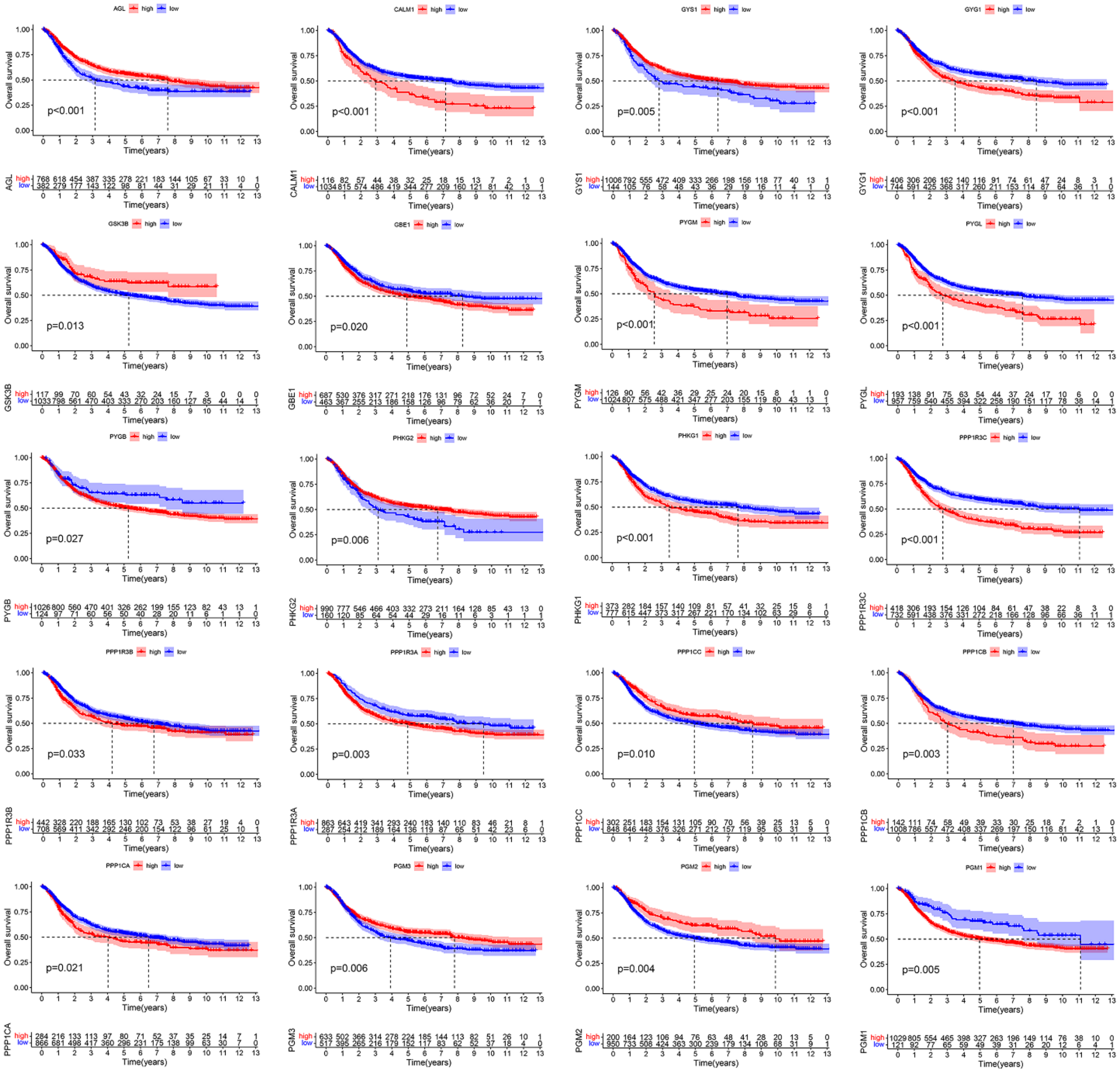

Figure S3: K-M curves demonstrated 20 glycogen metabolism genes (AGL, CALM1, GYG1, GBE1, GYS1, GSK3B, PYGM, PYGL, PYGB, PHKG1, PPP1R3C, PPP1R3B, PPP1R3A, PPP1CB, PPP1CA, PGM1, PHKG2, PPP1CC, PGM3 and PGM2) were significantly related with overall survival (OS) for gastric cancer (GC) patients in meta-cohort.

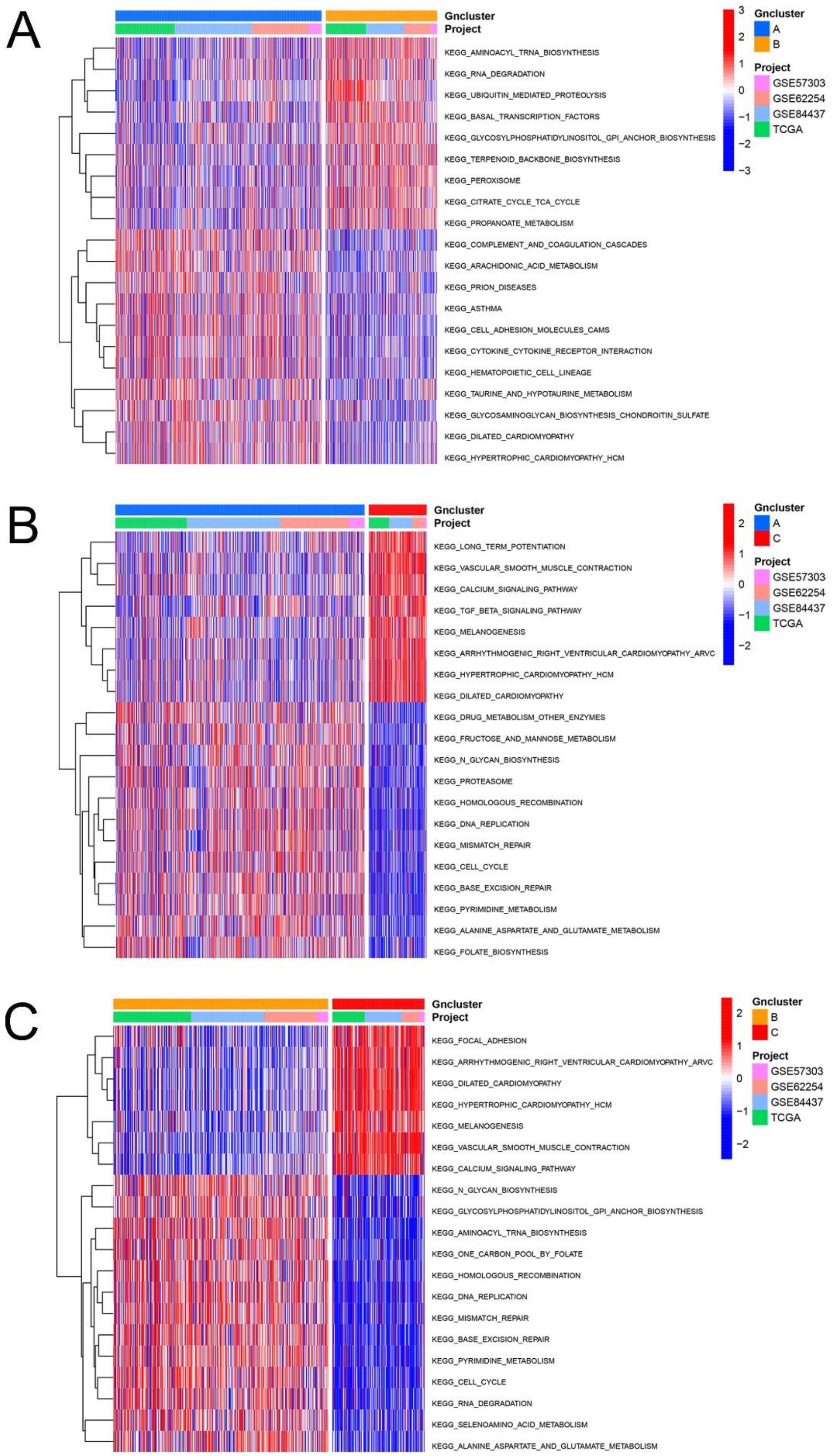

Figure S4: Gene set variation analysis (GSVA) Heatmap showing differences of Kyoto Encyclopedia of Genes and Genomes (KEGG) pathways in the three clusters. A: Cluster A vs. Cluster B. B: Cluster A vs. Cluster C. C: Cluster B vs. Cluster C.

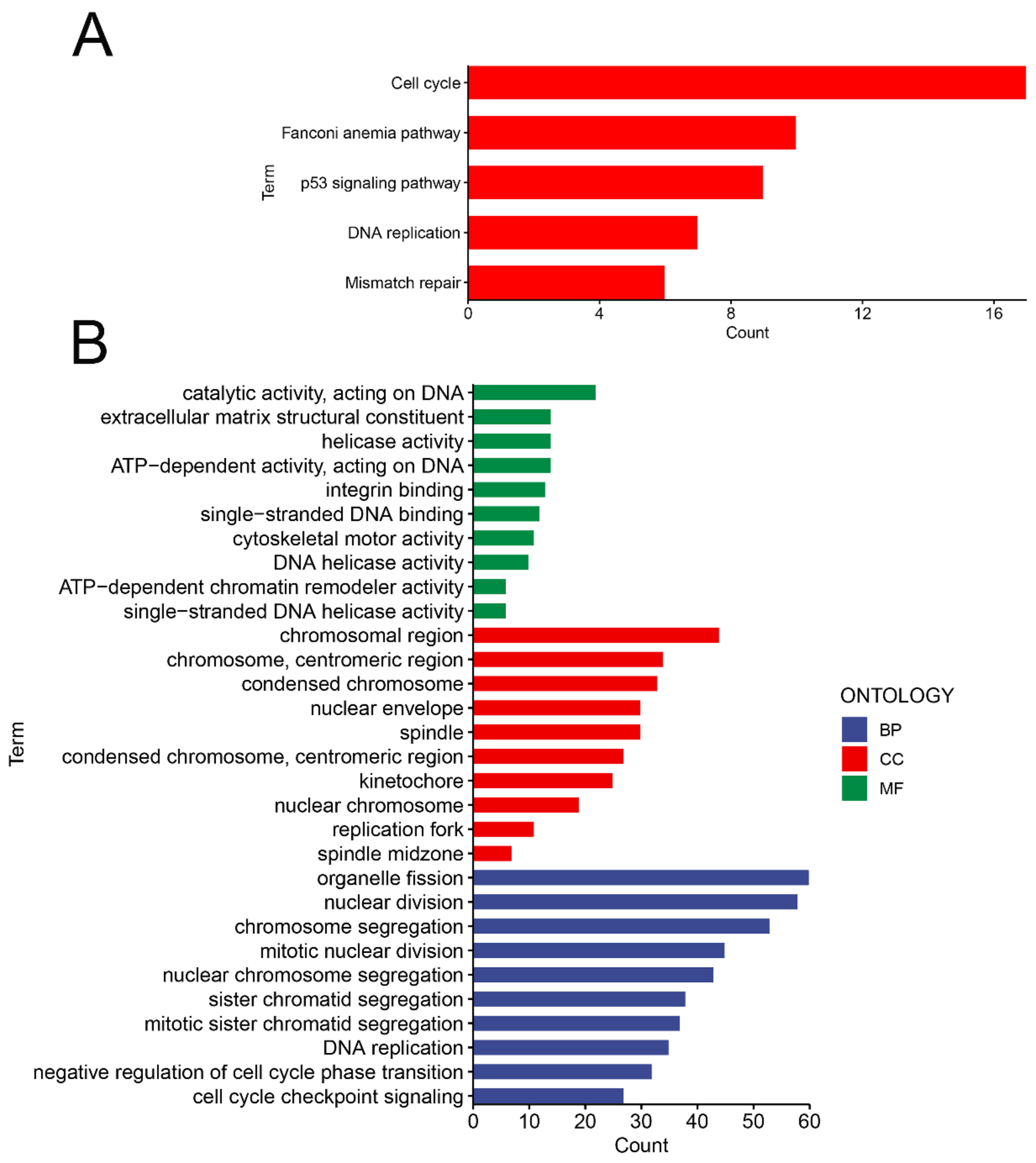

Figure S5: Kyoto Encyclopedia of Genes and Genomes (KEGG; A) and Gene Ontology (GO; B) enrichment analysis of the 508 intersected genes from the comparisons of the three clusters. BP: biological process; CC: cell component; MF: molecular function.

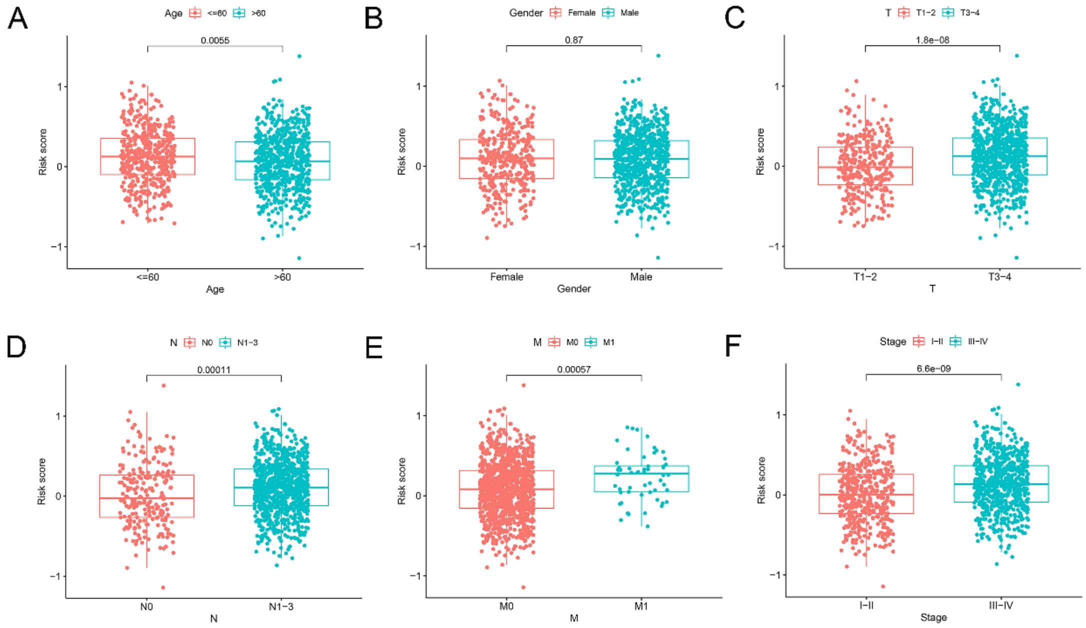

Figure S6: Difference of glycogen riskScore in gastric cancer (GC) patients with different age range (A), gender (B), T (C), N (D), M (E) and stage (F).

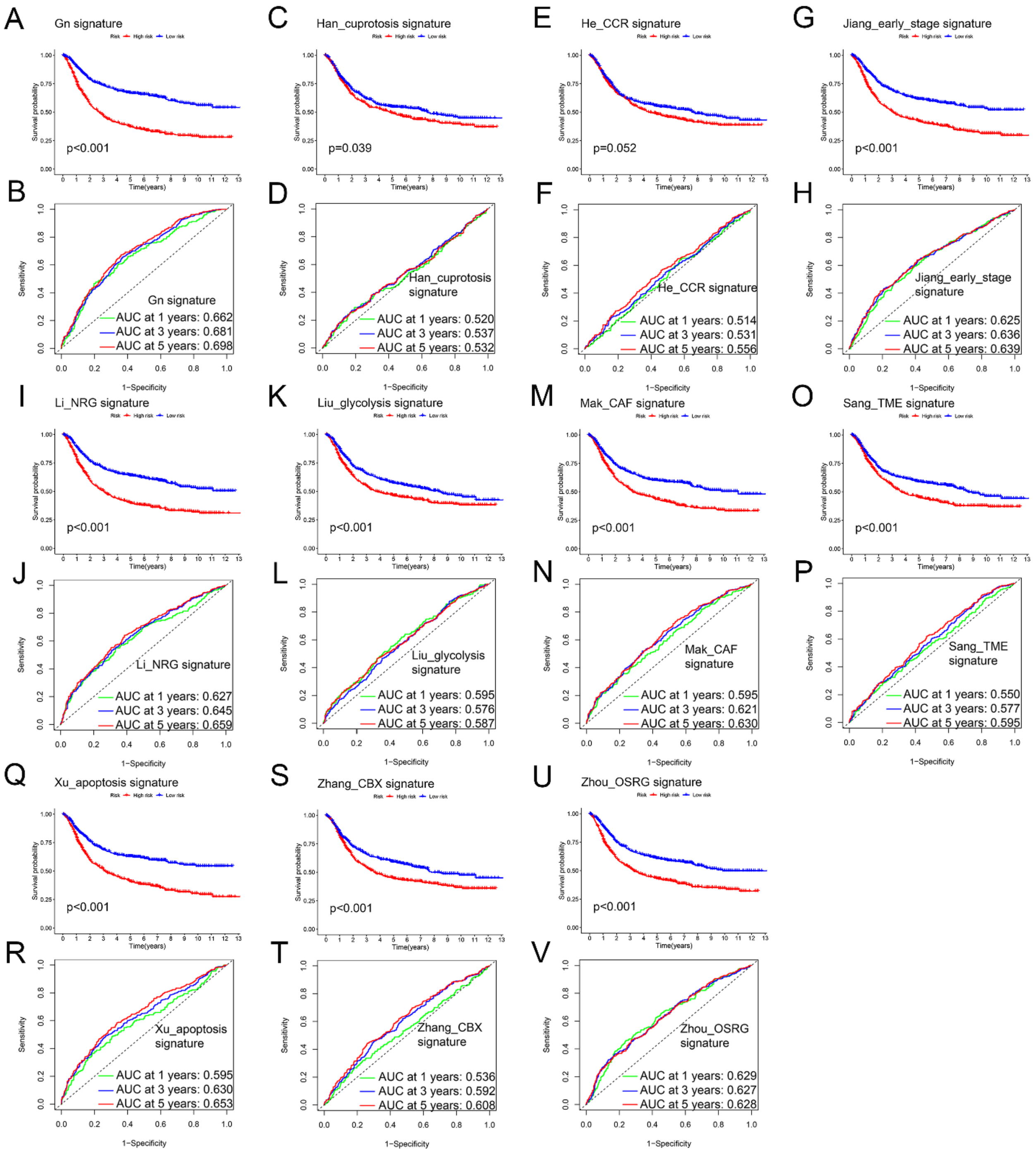

Figure S7: Kaplan-Meier (K-M) curves and time-dependent receiver operating characteristic (ROC) curves at 1, 3, and 5 years in meta-cohort for our glycogen riskScore (A & B), Han's cuprotosis signature (C & D), He's CCR signature (E & F), Jiang's early-stage signature (G & H), Li's NRG signature (I & J), Liu's glycolysis signature (K & L), Mak's CAF signature (M & N), Sang's TME signature (O & P), Xu's apoptosis signature (Q & R), Zhang's CBX signature (S & T), and Zhou's OSRG signature (U & V). CCR: chemokine and chemokine receptor; NRG: necroptosis-related gene; CAF: cancer-associated fibroblast; TME: tumor microenvironment; CBX: chromobox; OSRG: oxidative stress related gene.

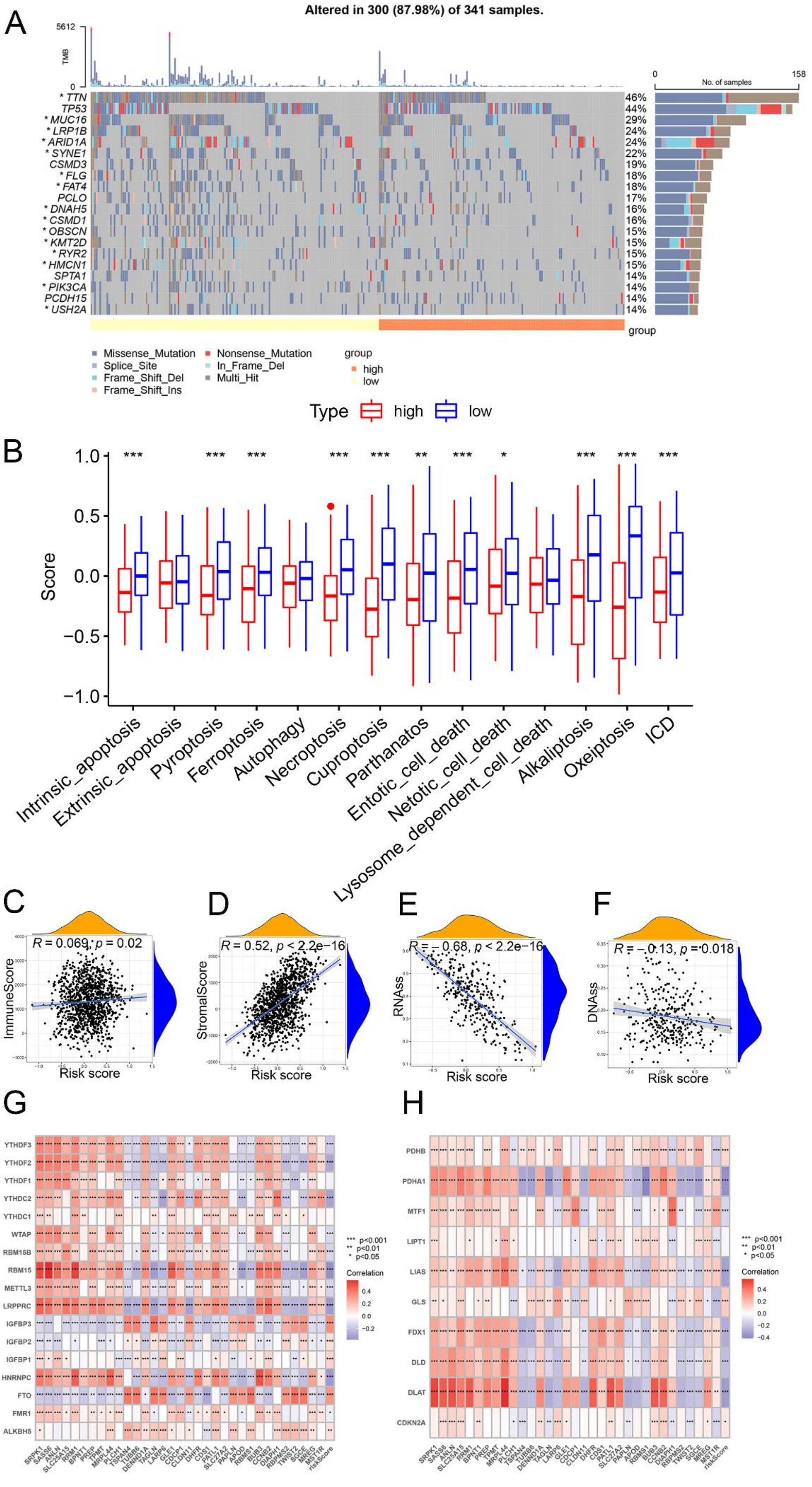

A
Altered in 300 (87.98%) of 341 samples.
TMB
5612
0
No. of samples
158
* TTN 46%
TP53 44%
* MUC16 29%
* LRP1B 24%
* ARID1A 24%
* SYNE1 22%
CSMD3 19%
* FLG 18%
* FAT4 18%
PCLO 17%
* DNAH5 16%
* CSMD1 16%
* OBSCN 15%
* KMT2D 15%
* RYR2 15%
* HMCN1 15%
SPTA1 14%
* PIK3CA 14%
PCDH15 14%
* USH2A 14%
group
Missense_Mutation
Splice_Site
Frame_Shift_Del
Frame_Shift_Ins
Nonsense_Mutation
In_Frame_Del
Multi_Hit
group
high
low
Type high low
B
Score
1.0
0.5
0.0
−0.5
−1.0
*** *** *** *** *** ** *** * *** *** ***
Intrinsic_apoptosis
Extrinsic_apoptosis
Pyroptosis
Ferroptosis
Autophagy
Necroptosis
Cuproptosis
Parthanatos
Entotic_cell_death
Netotic_cell_death
Lysosome_dependent_cell_death
Alkaliptosis
Oxeiptosis
ICD
C
R = 0.069, p = 0.02
ImmuneScore
Risk score
D
R = 0.52, p < 2.2e−16
StromalScore
Risk score
E
R = − 0.68, p < 2.2e−16
RNAss
Risk score
F
R = − 0.13, p = 0.018
DNAss
Risk score
G
YTHDF3
YTHDF2
YTHDF1
YTHDC2
YTHDC1
WTAP
RBM15B
RBM15
METTL3
LRPPRC
IGFBP3
IGFBP2
IGFBP1
HNRNPC
FTO
FMR1
ALKBH5
*** p<0.001
** p<0.01
* p<0.05
Correlation
0.4
0.2
0.0
−0.2
H
PDHB
PDHA1
MTF1
LIPT1
LIAS
GLS
FDX1
DLD
DLAT
CDKN2A
*** p<0.001
** p<0.01
* p<0.05
Correlation
0.4
0.2
0.0
−0.2
−0.4

Figure S8: Correlation of glycogen riskScore with tumor microenvironment (TME) and functional molecules in gastric cancer (GC) patients. A: Genetic mutation landscape of GC patients in low- and high-risk group. B: Different levels of 14 programmed cell death (PCD) patterns in GC patients with low and high riskScore. C-D: Correlation of glycogen riskScore with immune score (C) and stromal score (D). E-F: Relationship of glycogen riskScore and tumour stemness (E: mRNA expression-based stemness scores (RNAss); F: DNA methylation-based stemness scores (DNAss)). G-H: Association of glycogen riskScore with m6A regulators (G) and cuproptosis genes (H). ICD: immunogenic cell death.

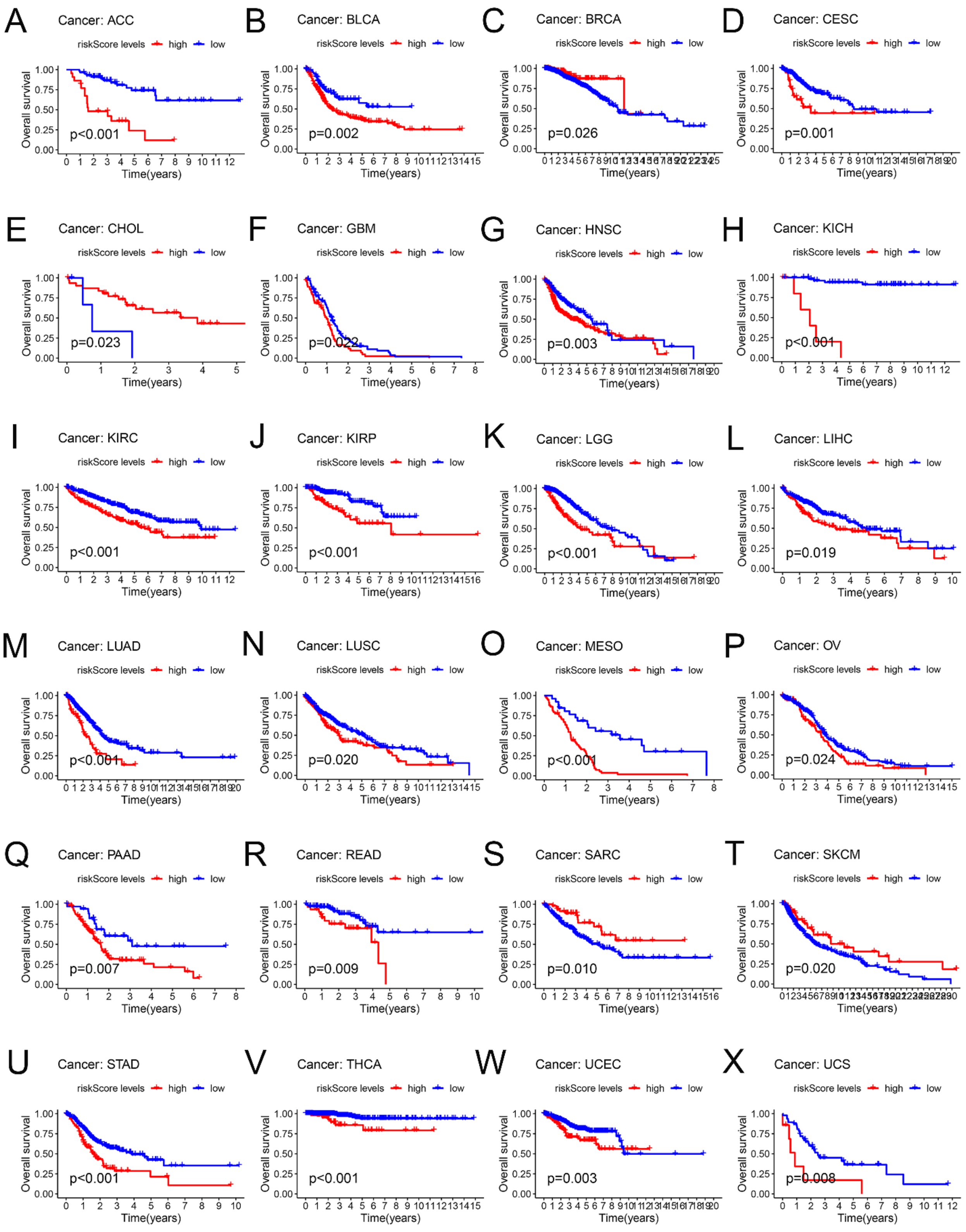

Figure S9: Kaplan-Meier (K-M) curves demonstrated glycogen riskScore as predictors in prognosis of 24 types of tumors. ACC: adrenocortical carcinoma; BLCA: bladder urothelial carcinoma; BRCA: breast invasive carcinoma; CESC: cervical squamous cell carcinoma and endocervical adenocarcinoma; CHOL: cholangiocarcinoma; GBM: glioblastoma multiforme; HNSC: head and neck squamous cell carcinoma; KICH:

kidney chromophobe; KIRC: kidney renal clear cell carcinoma; KIRP: kidney renal papillary cell carcinoma; LGG: brain lower grade glioma; LIHC: liver hepatocellular carcinoma; LUAD: lung adenocarcinoma; LUSC: lung squamous cell carcinoma; MESO: mesothelioma; OV: ovarian serous cystadenocarcinoma; PAAD: pancreatic adenocarcinoma; READ: rectum adenocarcinoma; SARC: sarcoma; SKCM: skin cutaneous melanoma; STAD: stomach adenocarcinoma; THCA: thyroid carcinoma; UCEC: uterine corpus endometrial carcinoma; UCS: uterine carcinosarcoma.

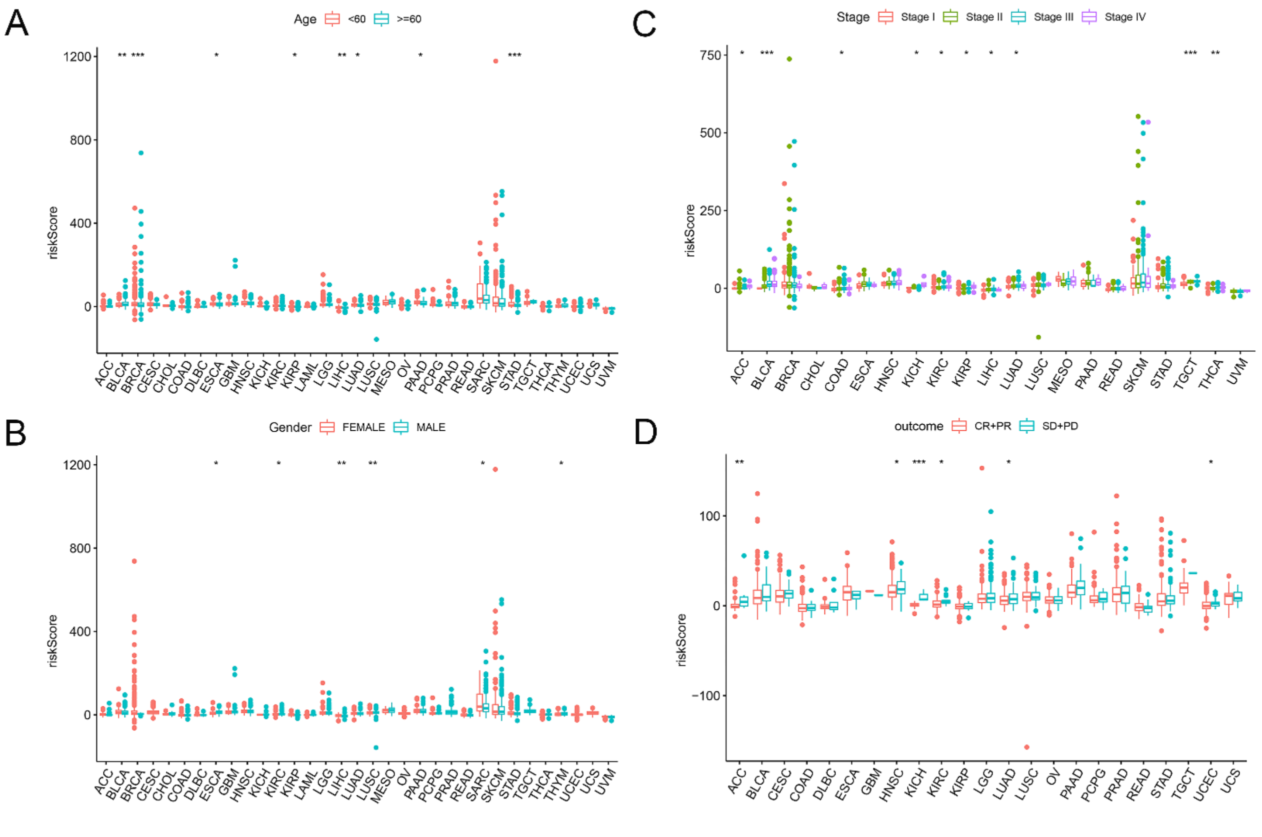

Figure S10: Distribution of glycogen riskScore in patients with different clinical parameters in pan-cancer. CR: complete response; PR: partial response; SD: stable disease; PD: progressive disease.

Table S1 Differences of KEGG pathways in the three clusters

Cluster A vs. B	Cluster A vs. C	Cluster B vs. C
KEGG_UBIQUITIN_MEDIATED_PROTEOLYSIS	KEGG_VASCULAR_SMOOTH_MUSCLE_CONTRACTION	KEGG_VASCULAR_SMOOTH_MUSCLE_CONTRACTION
KEGG_COMPLEMENT_AND_COAGULATION_CASCADES	KEGG_BASE_EXCISION_REPAIR	KEGG_DILATED_CARDIOMYOPATHY
KEGG_AMINOACYL_TRNA_BIOSYNTHESIS	KEGG_CALCIUM_SIGNALING_PATHWAY	KEGG_CALCIUM_SIGNALING_PATHWAY
KEGG_ARACHIDONIC_ACID_METABOLISM	KEGG_HYPERTROPHIC_CARDIOMYOPATHY_HCM	KEGG_HYPERTROPHIC_CARDIOMYOPATHY_HCM
KEGG_BASAL_TRANSCRIPTION_FACTORS	KEGG_LONG_TERM_POTENTIATION	KEGG_AMINOACYL_TRNA_BIOSYNTHESIS
KEGG_CELL_ADHESION_MOLECULES_CAMS	KEGG_DNA_REPLICATION	KEGG_BASE_EXCISION_REPAIR
KEGG_DILATED_CARDIOMYOPATHY	KEGG_DILATED_CARDIOMYOPATHY	KEGG_PYRIMIDINE_METABOLISM
KEGG_GLYCOSYLPHOSPHATIDYLINOSITOL_GPI_ANCHOR_BIOSYNTHESIS	KEGG_MELANOGENESIS	KEGG_CELL_CYCLE
KEGG_PRION_DISEASES	KEGG_ARRHYTHMOGENIC_RIGHT_VENTRICULAR_CARDIOMYOPATHY_ARVC	KEGG_ARRHYTHMOGENIC_RIGHT_VENTRICULAR_CARDIOMYOPATHY_ARVC
KEGG_CYTOKINE_CYTOKINE_RECEPTOR_INTERACTION	KEGG_ALANINE_ASPARTATE_AND_GLUTAMATE_METABOLISM	KEGG_DNA_REPLICATION
KEGG_HEMATOPOIETIC_CELL_LINEAGE	KEGG_DRUG_METABOLISM_OTHER_ENZYMES	KEGG_MISMATCH_REPAIR
KEGG_RNA_DEGRADATION	KEGG_PYRIMIDINE_METABOLISM	KEGG_ONE_CARBON_POOL_BY_FOLATE
KEGG_CITRATE_CYCLE_TCA_CYCLE	KEGG_N_GLYCAN_BIOSYNTHESIS	KEGG_N_GLYCAN_BIOSYNTHESIS
KEGG_PEROXISOME	KEGG_FOLATE_BIOSYNTHESIS	KEGG_MELANOGENESIS
KEGG_HYPERTROPHIC_CARDIOMYOPATHY_HCM	KEGG_CELL_CYCLE	KEGG_HOMOLOGOUS_RECOMBINATION
KEGG_GLYCOSAMINOGLYCAN_BIOSYNTHESIS_CHONDROITIN_SULFATE	KEGG_FRUCTOSE_AND_MANNOSE_METABOLISM	KEGG_RNA_DEGRADATION
KEGG_PROPANOATE_METABOLISM	KEGG_HOMOLOGOUS_RECOMBINATION	KEGG_SELENOAMINO_ACID_METABOLISM
KEGG_TERPENOID_BACKBONE_BIOSYNTHESIS	KEGG_TGF_BETA_SIGNALING_PATHWAY	KEGG_FOCAL_ADHESION

KEGG_TAURINE_AND_HYPOTAURINE_METABOLISM	KEGG_MISMATCH_REPAIR	KEGG_GLYCOSYLPHOSPHATIDYLINOSITOL_GPI_ANCHOR_BIOSYNTHESIS
KEGG_ASTHMA	KEGG_PROTEASOME	KEGG_ALANINE_ASPARTATE_AND_GLUTAMATE_METABOLISM
KEGG_ONE_CARBON_POOL_BY_FOLATE	KEGG_WNT_SIGNALING_PATHWAY	KEGG_NUCLEOTIDE_EXCISION_REPAIR
KEGG_VALINE_LEUCINE_AND_ISOLEUCINE_DEGRADATION	KEGG_FOCAL_ADHESION	KEGG_PROTEASOME
KEGG_GLYOXYLATE_AND_DICARBOXYLATE_METABOLISM	KEGG_STEROID_BIOSYNTHESIS	KEGG_TERPENOID_BACKBONE_BIOSYNTHESIS
KEGG_CELL_CYCLE	KEGG_PENTOSE_PHOSPHATE_PATHWAY	KEGG_FOLATE_BIOSYNTHESIS
KEGG_SYSTEMIC_LUPUS_ERYTHEMATOSUS	KEGG_ONE_CARBON_POOL_BY_FOLATE	KEGG_ECM_RECEPTOR_INTERACTION
KEGG_LYSINE_DEGRADATION	KEGG_P53_SIGNALING_PATHWAY	KEGG_RNA_POLYMERASE
KEGG_GLYCOSPHINGOLIPID_BIOSYNTHESIS_GLOBO_SERIES	KEGG_AMINOACYL_TRNA_BIOSYNTHESIS	KEGG_SPLICEOSOME
KEGG_LEUKOCYTE_TRANSENDOTHELIAL_MIGRATION	KEGG_GLUTATHIONE_METABOLISM	KEGG_P53_SIGNALING_PATHWAY
KEGG_ECM_RECEPTOR_INTERACTION	KEGG_RIBOFLAVIN_METABOLISM	KEGG_REGULATION_OF_ACTIN_CYTOSKELETON
KEGG_PYRUVATE_METABOLISM	KEGG_REGULATION_OF_ACTIN_CYTOSKELETON	KEGG_FRUCTOSE_AND_MANNOSE_METABOLISM
KEGG_INTESTINAL_IMMUNE_NETWORK_FOR_IGA_PRODUCTION	KEGG_ADHERENS_JUNCTION	KEGG_GLYOXYLATE_AND_DICARBOXYLATE_METABOLISM
KEGG_VIRAL_MYOCARDITIS	KEGG_CYTOSOLIC_DNA_SENSING_PATHWAY	KEGG_CYSTEINE_AND_METHIONINE_METABOLISM
KEGG_NUCLEOTIDE_EXCISION_REPAIR	KEGG_VASOPRESSIN_REGULATED_WATER_REABSORPTION	KEGG_PEROXISOME
KEGG_GLYCOSPHINGOLIPID_BIOSYNTHESIS_GANGLIO_SERIES	KEGG_SELENOAMINO_ACID_METABOLISM	KEGG_BASAL_TRANSCRIPTION_FACTORS
KEGG_NON_HOMOLOGOUS_END_JOINING	KEGG_BLADDER_CANCER	KEGG_PENTOSE_PHOSPHATE_PATHWAY
KEGG_RNA_POLYMERASE	KEGG_PHOSPHATIDYLINOSITOL_SIGNALING_SYSTEM	KEGG_GLYCOSPHINGOLIPID_BIOSYNTHESIS_GANGLIO_SERIES

KEGG_PRIMARY_IMMUNODEFICIENCY	KEGG_SPHINGOLIPID_METABOLISM	KEGG_DRUG_METABOLISM_OTHER_ENZYMES
KEGG_SPLICEOSOME	KEGG_SPLICEOSOME	KEGG_MAPK_SIGNALING_PATHWAY
KEGG_AUTOIMMUNE_THYROID_DISEASE	KEGG_MTOR_SIGNALING_PATHWAY	KEGG_CITRATE_CYCLE_TCA_CYCLE
KEGG_BASE_EXCISION_REPAIR	KEGG_RNA_DEGRADATION	KEGG_NON_HOMOLOGOUS_END_JOINING
KEGG_DRUG_METABOLISM_CYTOCHROME_P450	KEGG_GAP_JUNCTION	KEGG_TGF_BETA_SIGNALING_PATHWAY
KEGG_MISMATCH_REPAIR	KEGG_RNA_POLYMERASE	KEGG_CELL_ADHESION_MOLECULES_CAMS
KEGG_ALLOGRAFT_REJECTION	KEGG_CYSTEINE_AND_METHIONINE_METABOLISM	KEGG_SPHINGOLIPID_METABOLISM
KEGG_HOMOLOGOUS_RECOMBINATION	KEGG_NUCLEOTIDE_EXCISION_REPAIR	KEGG_LEUKOCYTE_TRANSENDOTHELIAL_MIGRATION
KEGG_TYPE_I_DIABETES_MELLITUS	KEGG_TERPENOID_BACKBONE_BIOSYNTHESIS	KEGG_RIBOFLAVIN_METABOLISM
KEGG_GRAFT_VERSUS_HOST_DISEASE	KEGG_ECM_RECEPTOR_INTERACTION	KEGG_STEROID_BIOSYNTHESIS
	KEGG_PENTOSE_AND_GLUCURONATE_INTERCONVERSIONS	KEGG_THYROID_CANCER
	KEGG_PORPHYRIN_AND_CHLOROPHYLL_METABOLISM	KEGG_GAP_JUNCTION
	KEGG_PANTOTHENATE_AND_COA_BIOSYNTHESIS	KEGG_GLYCOSAMINOGLYCAN_BIOSYNTHESIS_CHONDROITIN_SULFATE
	KEGG_GALACTOSE_METABOLISM	KEGG_COMPLEMENT_AND_COAGULATION_CASCADES
	KEGG_GLYOXYLATE_AND_DICARBOXYLATE_METABOLISM	KEGG_OOCYTE_MEIOSIS
	KEGG_VIBRIO_CHOLERAE_INFECTION	KEGG_BLADDER_CANCER
	KEGG_GLYCOSPHINGOLIPID_BIOSYNTHESIS_GANGLIO_SERIES	KEGG_VASOPRESSIN_REGULATED_WATER_REABSORPTION
	KEGG_NON_HOMOLOGOUS_END_JOINING	KEGG_AXON_GUIDANCE
	KEGG_MATURITY_ONSET_DIABETES_OF_THE_YOUNG	KEGG_PURINE_METABOLISM
	KEGG_ANTIGEN_PROCESSING_AND_PRESENTATION	KEGG_NEUROACTIVE_LIGAND_RECEPTOR_INTERACTION
	KEGG_INOSITOL_PHOSPHATE_METABOLISM	KEGG_LONG_TERM_POTENTIATION
	KEGG_NOD_LIKE_RECEPTOR_SIGNALING_PATHWAY	KEGG_GALACTOSE_METABOLISM
	KEGG_GLYCOSYLPHOSPHATIDYLINOSITOL_GPI_ANCHOR_BIOSYNTHESIS	KEGG_PANTOTHENATE_AND_COA_BIOSYNTHESIS

	KEGG_BASAL_CELL_CARCINOMA	KEGG_GLYCEROPHOSPHOLIPID_METABOLISM
	KEGG_HUNTINGTONS_DISEASE	KEGG_BASAL_CELL_CARCINOMA
	KEGG_GRAFT_VERSUS_HOST_DISEASE	KEGG_BIOSYNTHESIS_OF_UNSATURATED_FATTY_ACIDS
	KEGG_ETHER_LIPID_METABOLISM	KEGG_CYTOSOLIC_DNA_SENSING_PATHWAY
	KEGG_GLYCOSAMINOGLYCAN_BIOSYNTHESIS_KERATAN_SULFATE	KEGG_TAURINE_AND_HYPOTAURINE_METABOLISM
	KEGG_GLYCOLYSIS_GLUCONEOGENESIS	KEGG_WNT_SIGNALING_PATHWAY
	KEGG_PEROXISOME	KEGG_PROTEIN_EXPORT
	KEGG_GLYCOSPHINGOLIPID_BIOSYNTHESIS_LACTO_AND_NEOLACTO_SERIES	KEGG_AMINO_SUGAR_AND_NUCLEOTIDE_SUGAR_METABOLISM
	KEGG_GLYCINE_SERINE_AND_THREONINE_METABOLISM	KEGG_PENTOSE_AND_GLUCURONATE_INTERCONVERSIONS
	KEGG_BIOSYNTHESIS_OF_UNSATURATED_FATTY_ACIDS	KEGG_UBIQUITIN_MEDIATED_PROTEOLYSIS
	KEGG_PROTEIN_EXPORT	KEGG_MELANOMA
	KEGG_TYPE_I_DIABETES_MELLITUS	KEGG_HUNTINGTONS_DISEASE
	KEGG_APOPTOSIS	KEGG_PYRUVATE_METABOLISM
	KEGG_CITRATE_CYCLE_TCA_CYCLE	KEGG_PORPHYRIN_AND_CHLOROPHYLL_METABOLISM
	KEGG_NATURAL_KILLER_CELL_MEDIATED_CYTOTOXICITY	KEGG_GLUTATHIONE_METABOLISM
	KEGG_AMINO_SUGAR_AND_NUCLEOTIDE_SUGAR_METABOLISM	KEGG_HEDGEHOG_SIGNALING_PATHWAY
	KEGG_SULFUR_METABOLISM	KEGG_HEMATOPOIETIC_CELL_LINEAGE
	KEGG_GLYCOSAMINOGLYCAN_BIOSYNTHESIS_CHONDROITIN_SULFATE	KEGG_ADHERENS_JUNCTION
	KEGG_OXIDATIVE_PHOSPHORYLATION	KEGG_NON_SMALL_CELL_LUNG_CANCER
	KEGG_PRIMARY_IMMUNODEFICIENCY	KEGG_ARACHIDONIC_ACID_METABOLISM
	KEGG_LINOLEIC_ACID_METABOLISM	KEGG_TYROSINE_METABOLISM

	KEGG_OTHER_GLYCAN_DEGRADATION	KEGG_GLYCOLYSIS_GLUCONEOGENESIS
	KEGG_ALLOGRAFT_REJECTION	KEGG_CARDIAC_MUSCLE_CONTRACTION
		KEGG_LYSINE_DEGRADATION
		KEGG_PRION_DISEASES
		KEGG_APOPTOSIS
		KEGG_MTOR_SIGNALING_PATHWAY
		KEGG_VIBRIO_CHOLERAE_INFECTION
		KEGG_SYSTEMIC_LUPUS_ERYTHEMATOSUS
		KEGG_ALZHEIMERS_DISEASE
		KEGG_PRIMARY_BILE_ACID_BIOSYNTHESIS
		KEGG_CHEMOKINE_SIGNALING_PATHWAY
		KEGG_CYTOKINE_CYTOKINE_RECEPTOR_INTERACTION
		KEGG_NOD_LIKE_RECEPTOR_SIGNALING_PATHWAY
		KEGG_GLYCOSPHINGOLIPID_BIOSYNTHESIS_LACTO_AND_NEOLACTO_SERIES
		KEGG_ASTHMA
		KEGG_ETHER_LIPID_METABOLISM
		KEGG_VALINE_LEUCINE_AND_ISOLEUCINE_DEGRADATION
		KEGG_PHENYLALANINE_METABOLISM
		KEGG_BUTANOATE_METABOLISM
		KEGG_MATURITY_ONSET_DIABETES_OF_THE_YOUNG
		KEGG_DRUG_METABOLISM_CYTOCHROME_P450
		KEGG_O_GLYCAN_BIOSYNTHESIS
		KEGG_VIRAL_MYOCARDITIS
		KEGG_ANTIGEN_PROCESSING_AND_PRESENTATION

	KEGG_RENIN_ANGIOTENSIN_SYSTEM
	KEGG_OTHER_GLYCAN_DEGRADATION
	KEGG_OXIDATIVE_PHOSPHORYLATION
	KEGG_INTESTINAL_IMMUNE_NETWORK_FOR_IGA_PRODUCTION
	KEGG_GRAFT_VERSUS_HOST_DISEASE

Table S2. Anti-tumor drugs with significant relationship with the riskScore in the Cell Miner database.

Drug	cor	pvalue
Tamoxifen	-0.53152	1.24E-05
Dasatinib	0.487796	7.71E-05
Erlotinib	0.475286	0.000124
Nelarabine	-0.47341	0.000133
Raloxifene	-0.45623	0.000249
Crizotinib	-0.45158	0.000293
Ixabepilone	-0.45154	0.000293
Nilotinib	-0.43291	0.000551
Ifosfamide	-0.42552	0.0007
Lenvatinib	0.423613	0.000745
Cyclophosphamide	-0.42062	0.000819
Tegafur	-0.41536	0.000966
Simvastatin	0.409153	0.001171
Dexrazoxane	-0.40186	0.001459
Carmustine	-0.38756	0.002218

Palbociclib	-0.38567	0.00234
Fluorouracil	-0.38458	0.002414
Pipamperone	-0.38162	0.002625
Zoledronate	0.373371	0.0033
ETHINYL ESTRADIOL	-0.37247	0.003382
Bleomycin	0.372057	0.00342
Irofulven	0.371497	0.003473
Lomustine	-0.37113	0.003508
Paclitaxel	-0.36635	0.003992
LDK-378	-0.36313	0.004349
Everolimus	0.358534	0.004909
Oxaliplatin	-0.35416	0.0055
Vinblastine	-0.34736	0.006542
Eribulin mesilate	-0.3426	0.007372
DECITABINE	-0.33966	0.007929
Pipobroman	-0.33383	0.009142
Vorinostat	-0.32868	0.010346
Acrichine	-0.32818	0.01047
Fluphenazine	-0.32413	0.01152
Vandetanib	0.32194	0.012126
Sonidegib	0.317987	0.013289
Ibrutinib	0.315641	0.014023
Docetaxel	-0.31552	0.014063
TYROTHRICIN	-0.3155	0.014069
Asparaginase	-0.31244	0.015082

METHOTREXATE	-0.29912	0.02025
VINORELBINE	-0.29786	0.020812
Estramustine	-0.29592	0.021696
Homoharringtonine	-0.28922	0.025007
Vincristine	-0.28646	0.02649
Etoposide	-0.28536	0.027098
Dacomitinib	0.281927	0.029085
Belinostat	-0.28136	0.029426
Dromostanolone Propionate	-0.27607	0.032747
Arsenic trioxide	-0.26935	0.037425
Idarubicin	-0.26841	0.038118
Actinomycin D	-0.26534	0.040462
Cytarabine	-0.26081	0.044146
DAUNORUBICIN	-0.25823	0.046362
JNJ-42756493	0.256009	0.048341
Teniposide	-0.25565	0.048672

Table S3. Significant KEGG and GO enrichment pathways based on glycogen riskScore.

ID	pvalue	ID	pvalue
KEGG_NEUROACTIVE_LIGAND_RECEPTOR_INTERACTION	0.01	GOBP_AXON_DEVELOPMENT	0.01

KEGG_REGULATION_OF_ACTIN_CYTOSKELETON	0.010101	GOBP_CELL_CELL_SIGNALING_BY_WNT	0.01
KEGG_FOCAL_ADHESION	0.010204	GOBP_CELL_MORPHOGENESIS_INVOLVED_IN_NEURON_DIFFERENTIATION	0.01
KEGG_WNT_SIGNALING_PATHWAY	0.010204	GOBP_CELL_SURFACE_RECEPTOR_SIGNALING_PATHWAY_INVOLVED_IN_CELL_CELL_SIGNALING	0.01
KEGG_CALCIUM_SIGNALING_PATHWAY	0.010309	GOBP_EXTERNAL_ENCAPSULATING_STRUCTURE_ORGANIZATION	0.01
KEGG_AXON_GUIDANCE	0.010417	GOBP_MESENCHYME_DEVELOPMENT	0.01
KEGG_CELL_ADHESION_MOLECULES_CAMS	0.010417	GOBP_MUSCLE_CELL_DIFFERENTIATION	0.01
KEGG_ECM_RECEPTOR_INTERACTION	0.011364	GOBP_MUSCLE_CONTRACTION	0.01
KEGG_VASCULAR_SMOOTH_MUSCLE_CONTRACTION	0.011364	GOBP_MUSCLE_ORGAN_DEVELOPMENT	0.01

KEGG_HYPERTROPHIC_CARDIOMYOPATHY_HCM	0.011494	GOBP_MUSCLE_SYSTEM_PROCESS	0.01
KEGG_ARRHYTHMOGENIC_RIGHT_VENTRICULAR_CARDIOMYOPATHY_ARVC	0.011765	GOBP_MUSCLE_TISSUE_DEVELOPMENT	0.01
KEGG_COMPLEMENT_AND_COAGULATION_CASCADES	0.011905	GOBP_OSSIFICATION	0.01
KEGG_DILATED_CARDIOMYOPATHY	0.011905	GOBP_REGULATION_OF_NEURON_PROJECTION_DEVELOPMENT	0.01
KEGG_GLYCOSAMINOGLYCAN_BIOSYNTHESIS_CHONDROITIN_SULFATE	0.013699	GOBP_REGULATION_OF_SYSTEM_PROCESS	0.01
KEGG_MAPK_SIGNALING_PATHWAY	0.02	GOBP_SYNAPSE_ORGANIZATION	0.01
KEGG_GAP_JUNCTION	0.022727	GOCC_COLLAGEN_CONTAINING_EXTRACELLULAR_MATRIX	0.01
KEGG_TGF_BETA_SIGNALING_PATHWAY	0.022989	GOCC_CONTRACTILE_FIBER	0.010101
KEGG_BASAL_CELL_CARCINOMA	0.023256	GOMF_EXTRACELLULAR_MATRIX_STRUCTURAL_CONSTITUENT	0.010101

KEGG_MELANOGENESIS	0.02325 6	GOBP_OLIGODENDROCYTE_DIFFERENTIATION	0.01075 3
KEGG_PATHWAYS_IN_CANCER	0.02970 3	GOBP_SENSORY_PERCEPTION_OF_PAIN	0.01075 3
KEGG_GLYOXYLATE_AND_DICARBOXYLATE_METABOLISM	0.03225 8	GOBP_SPINAL_CORD_DEVELOPMENT	0.01075 3
KEGG_TERPENOID_BACKBONE_BIOSYNTHESIS	0.03225 8	GOBP_CELL_SUBSTRATE_JUNCTION_ORGANIZATION	0.01087
KEGG_TYROSINE_METABOLISM	0.03409 1	GOBP_CELLULAR_RESPONSE_TO_KETONE	0.01087
KEGG_MELANOMA	0.03488 4	GOBP_MESENCHYMAL_CELL_DEVELOPMENT	0.01087
KEGG_CARDIAC_MUSCLE_CONTRACTION	0.03529 4	GOBP_MORPHOGENESIS_OF_A_POLARIZED_EPITHELIUM	0.01087
KEGG_ONE_CARBON_POOL_BY_FOLATE	0.03571 4	GOBP_NEGATIVE_REGULATION_OF_VASCULATURE_DEVELOPMENT	0.01087

KEGG_PENTOSE_AND_GLUCURONATE_INTERCONVERSIONS	0.035714	GOBP_REGULATION_OF_ACTOMYOSIN_STRUCTURE_ORGANIZATION	0.01087
KEGG_HOMOLOGOUS_RECOMBINATION	0.037037	GOBP_REGULATION_OF_BIOMINERALIZATION	0.01087
KEGG_RNA_POLYMERASE	0.037037	GOCC_BASEMENT_MEMBRANE	0.01087
KEGG_AMINOACYL_TRNA_BIOSYNTHESIS	0.038462	GOCC_GOLGI_LUMEN	0.01087
KEGG_MISMATCH_REPAIR	0.038462	GOBP_CELL_DIFFERENTIATION_INVOLVED_IN_KIDNEY_DEVELOPMENT	0.011111
KEGG_GLYCOSPHINGOLIPID_BIOSYNTHESIS_GANGLIO_SERIES	0.041667	GOBP_CELLULAR_RESPONSE_TO_VASCULAR_ENDOTHELIAL_GROWTH_FACTOR_STIMULUS	0.011111
KEGG_BUTANOATE_METABOLISM	0.043478	GOBP_HOMOTYPIC_CELL_CELL_ADHESION	0.011111
KEGG_LEUKOCYTE_TRANSENDOTHELIAL_MIGRATION	0.044944	GOBP_METANEPHROS_DEVELOPMENT	0.011111

KEGG_ALLOGRAFT_REJECTION	0.045455	GOBP_MUSCLE_ORGAN_MORPHOGENESIS	0.011111
KEGG_GRAFT_VERSUS_HOST_DISEASE	0.045455	GOBP_NERVE_DEVELOPMENT	0.011111
KEGG_ADHERENS_JUNCTION	0.047619	GOBP_PROTEIN_LOCALIZATION_TO_CELL_JUNCTION	0.011111
KEGG_ALANINE_ASPARTATE_AND_GLUTAMATE_METABOLISM	0.047619	GOBP_REGULATION_OF_EPITHELIAL_TO_MESENCHYMAL_TRANSITION	0.011111
KEGG_BASE_EXCISION_REPAIR	0.047619	GOBP_REGULATION_OF_LIPASE_ACTIVITY	0.011111
KEGG_CITRATE_CYCLE_TCA_CYCLE	0.047619	GOBP_SOMITE_DEVELOPMENT	0.011111
KEGG_FRUCTOSE_AND_MANNOSE_METABOLISM	0.047619	GOCC_ANCHORED_COMPONENT_OF_PLASMA_MEMBRANE	0.011111
		GOCC_MAIN_AXON	0.011111

	GOCC_POTASSIUM_CHANNEL_COMPLEX	0.011111
	GOMF_VOLTAGE_GATED_POTASSIUM_CHANNEL_ACTIVITY	0.011111
	GOBP_ADENYLATE_CYCLASE_INHIBITING_G_PROTEIN_COUPLED_RECEPTOR_SIGNALING_PATHWAY	0.011236
	GOBP_ARTERY_MORPHOGENESIS	0.011236
	GOBP_CALCIUM_ION_REGULATED_EXOCYTOSIS	0.011236
	GOBP_CARDIAC_MUSCLE_CELL_CONTRACTION	0.011236
	GOBP_CARDIAC_MUSCLE_TISSUE_MORPHOGENESIS	0.011236
	GOBP_CYCLIC_NUCLEOTIDE_MEDIATED_SIGNALING	0.011236

	GOBP_ENDOCHONDRAL_BONE_MORPHOGENESIS	0.011236
	GOBP_ESTABLISHMENT_OF_TISSUE_POLARITY	0.011236
	GOBP_FOREBRAIN_CELL_MIGRATION	0.011236
	GOBP_LENS_DEVELOPMENT_IN_CAMERA_TYPE_EYE	0.011236
	GOBP_MATERNAL_PROCESS_INVOLVED_IN_FEMALE_PREGNANCY	0.011236
	GOBP_MEMBRANE_BIOGENESIS	0.011236
	GOBP_NEGATIVE_REGULATION_OF_CHEMOTAXIS	0.011236
	GOBP_NEGATIVE_REGULATION_OF_ION_TRANSMEMBRANE_TRANSPORTER_ACTIVITY	0.011236

	GOBP_NEGATIVE_REGULATION_OF_NEURON_DIFFERENTIATION	0.011236
	GOBP_NEGATIVE_REGULATION_OF_RESPONSE_TO_WOUNDING	0.011236
	GOBP_NEGATIVE_REGULATION_OF_TRANSFORMING_GROWTH_FACTOR_BETA_RECEPTOR_SIGNALING_PATHWAY	0.011236
	GOBP_NEPHRON_MORPHOGENESIS	0.011236
	GOBP_NEURAL_CREST_CELL_MIGRATION	0.011236
	GOBP_NEURAL_NUCLEUS_DEVELOPMENT	0.011236
	GOBP_NEUROBLAST_PROLIFERATION	0.011236
	GOBP_OVULATION_CYCLE	0.011236

	GOBP_PATHWAY_RESTRICTED_SMAD_PROTEIN_PHOSPHORYLATION	0.011236
	GOBP_PERIPHERAL_NERVOUS_SYSTEM_DEVELOPMENT	0.011236
	GOBP_POSITIVE_REGULATION_OF_ACTIN_FILAMENT_BUNDLE_ASSEMBLY	0.011236
	GOBP_POSITIVE_REGULATION_OF_CALCIUM_ION_TRANSMEMBRANE_TRANSPORT	0.011236
	GOBP_POSITIVE_REGULATION_OF_CATION_CHANNEL_ACTIVITY	0.011236
	GOBP_POSITIVE_REGULATION_OF_ENDOTHELIAL_CELL_PROLIFERATION	0.011236
	GOBP_POSITIVE_REGULATION_OF_FAT_CELL_DIFFERENTIATION	0.011236
	GOBP_POSITIVE_REGULATION_OF_NEURON_DIFFERENTIATION	0.011236

	GOBP_POSITIVE_REGULATION_OF_OSTEOBLAST_DIFFERENTIATION	0.011236
	GOBP_POSITIVE_REGULATION_OF_PHOSPHATIDYLINOSITOL_3_KINASE_SIGNALING	0.011236
	GOBP_POSITIVE_REGULATION_OF_PHOSPHOLIPASE_ACTIVITY	0.011236
	GOBP_PRESYNAPTIC_ENDOCYTOSIS	0.011236
	GOBP_PROTEOGLYCAN_BIOSYNTHETIC_PROCESS	0.011236
	GOBP_REGULATION_OF_CARTILAGE_DEVELOPMENT	0.011236
	GOBP_REGULATION_OF_ESTABLISHMENT_OF_PLANAR_POLARITY	0.011236
	GOBP_REGULATION_OF_HEART_GROWTH	0.011236

	GOBP_REGULATION_OF_MUSCLE_ADAPTATION	0.012366
	GOBP_REGULATION_OF_NEURAL_PRECURSOR_CELL_PROLIFERATION	0.012366
	GOBP_REGULATION_OF_NEUROTRANSMITTER_RECEPTOR_ACTIVITY	0.012366
	GOBP_REGULATION_OF_ORGAN_GROWTH	0.012366
	GOBP_REGULATION_OF_RECEPTOR_INTERNALIZATION	0.012366
	GOBP_REGULATION_OF_RHO_PROTEIN_SIGNAL_TRANSDUCTION	0.012366
	GOBP_REGULATION_OF_SMOOTH_MUSCLE_CONTRACTION	0.012366
	GOBP_REGULATION_OF_SUBSTRATE_ADHESION_DEPENDENT_CELL_SPREADING	0.012366

	GOBP_RESPONSE_TO_DOPAMINE	0.01<br>123<br>6
	GOBP_SKELETAL_MUSCLE_CELL_DIFFERENTIATION	0.01<br>123<br>6
	GOBP_SMAD_PROTEIN_SIGNAL_TRANSDUCTION	0.01<br>123<br>6
	GOBP_SMOOTH_MUSCLE_CELL_DIFFERENTIATION	0.01<br>123<br>6
	GOBP_TISSUE_REGENERATION	0.01<br>123<br>6
	GOCC_ACTOMYOSIN	0.01<br>123<br>6
	GOCC_CAVEOLA	0.01<br>123<br>6
	GOCC_GABA_ERGIC_SYNAPSE	0.01<br>123<br>6

	GOCC_LYSOSOMAL_LUMEN	0.01<br>123<br>6
	GOCC_PLATELET_ALPHA_GRANULE	0.01<br>123<br>6
	GOCC_POSTSYNAPTIC_DENSITY_MEMBRANE	0.01<br>123<br>6
	GOCC_SARCOPLASM	0.01<br>123<br>6
	GOMF_HORMONE_BINDING	0.01<br>123<br>6
	GOMF_SERINE_TYPE_ENDOPEPTIDASE_INHIBITOR_ACTIVITY	0.01<br>123<br>6
	GOBP_AORTA_DEVELOPMENT	0.01<br>136<br>4
	GOBP_BONE_REMODELING	0.01<br>136<br>4

	GOBP_CARDIAC_CELL_DEVELOPMENT	0.01<br>136<br>4
	GOBP_CARDIAC_SEPTUM_MORPHOGENESIS	0.01<br>136<br>4
	GOBP_CELLULAR_GLUCAN_METABOLIC_PROCESS	0.01<br>136<br>4
	GOBP_CELLULAR_RESPONSE_TO_CALCIUM_ION	0.01<br>136<br>4
	GOBP_CENTRAL_NERVOUS_SYSTEM_NEURON_DEVELOPMENT	0.01<br>136<br>4
	GOBP_COLLAGEN_FIBRIL_ORGANIZATION	0.01<br>136<br>4
	GOBP_DIENCEPHALON_DEVELOPMENT	0.01<br>136<br>4
	GOBP_EMBRYONIC_HEART_TUBE_DEVELOPMENT	0.01<br>136<br>4

	GOBP_ENDOCRINE_PROCESS	0.01<br>136<br>4
	GOBP_FIBROBLAST_GROWTH_FACTOR_RECEPTOR_SIGNALING_PA<br>THWAY	0.01<br>136<br>4
	GOBP_FOCAL_ADHESION_ASSEMBLY	0.01<br>136<br>4
	GOBP_GLOMERULUS_DEVELOPMENT	0.01<br>136<br>4
	GOBP_HEART_GROWTH	0.01<br>136<br>4
	GOBP_MESENCHYME_MORPHOGENESIS	0.01<br>136<br>4
	GOBP_MESONEPHRIC_TUBULE_MORPHOGENESIS	0.01<br>136<br>4
	GOBP_MIDBRAIN_DEVELOPMENT	0.01<br>136<br>4

	GOBP_MONOAMINE_TRANSPORT	0.011364
	GOBP_MUSCLE_CELL_MIGRATION	0.011364
	GOBP_MUSCLE_HYPERTROPHY	0.011364
	GOBP_NEGATIVE_REGULATION_OF_CELL_SUBSTRATE_ADHESION	0.011364
	GOBP_NEGATIVE_REGULATION_OF_ION_TRANSMEMBRANE_TRANSPORT	0.011364
	GOBP_NEGATIVE_REGULATION_OF_TRANSPORTER_ACTIVITY	0.011364
	GOBP_NEGATIVE_REGULATION_OF_WOUND_HEALING	0.011364
	GOBP_POSITIVE_REGULATION_OF_CELL_MORPHOGENESIS_INVOLVED_IN_DIFFERENTIATION	0.011364

	GOBP_POSITIVE_REGULATION_OF_SMOOTH_MUSCLE_CELL_PROLIFERATION	0.011364
	GOBP_PROTEIN_LOCALIZATION_TO_SYNAPSE	0.011364
	GOBP_PROTEOGLYCAN_METABOLIC_PROCESS	0.011364
	GOBP_REGULATION_OF_BONE_MINERALIZATION	0.011364
	GOBP_REGULATION_OF_CALCIUM_ION_TRANSMEMBRANE_TRANSPORTER_ACTIVITY	0.011364
	GOBP_REGULATION_OF_CALCIUM_ION_TRANSPORT_INTO_CYTOSOL	0.011364
	GOBP_REGULATION_OF_CELL_SUBSTRATE_JUNCTION_ORGANIZATION	0.011364
	GOBP_REGULATION_OF_EPITHELIAL_CELL_APOPTOTIC_PROCESS	0.011364

	GOBP_REGULATION_OF_POSTSYNAPSE_ORGANIZATION	0.011364
	GOBP_REGULATION_OF_POTASSIUM_ION_TRANSMEMBRANE_TRANSPORTER_ACTIVITY	0.011364
	GOBP_REGULATION_OF_SMOOTHENED_SIGNALING_PATHWAY	0.011364
	GOBP_REGULATION_OF_STRIATED_MUSCLE_CELL_DIFFERENTIATION	0.011364
	GOBP_REGULATION_OF_TISSUE_REMODELING	0.011364
	GOBP_RESPONSE_TO_AXON_INJURY	0.011364
	GOBP_RESPONSE_TO_VITAMIN	0.011364
	GOBP_ROOF_OF_MOUTH_DEVELOPMENT	0.011364

	GOBP_STRIATED_MUSCLE_CELL_PROLIFERATION	0.01<br>136<br>4
	GOBP_VASCULOGENESIS	0.01<br>136<br>4
	GOBP_VENTRICULAR_CARDIAC_MUSCLE_TISSUE_DEVELOPMENT	0.01<br>136<br>4
	GOBP_VENTRICULAR_SEPTUM_DEVELOPMENT	0.01<br>136<br>4
	GOCC_COLLAGEN_TRIMER	0.01<br>136<br>4
	GOCC_INTRINSIC_COMPONENT_OF_PRESYNAPTIC_MEMBRANE	0.01<br>136<br>4
	GOCC_PLATELET_ALPHA_GRANULE_LUMEN	0.01<br>136<br>4
	GOMF_COLLAGEN_BINDING	0.01<br>136<br>4

	GOMF_SMAD_BINDING	0.011364
	GOMF_TRANSMEMBRANE_RECEPTOR_PROTEIN_KINASE_ACTIVITY	0.011364
	GOBP_ADULT_LOCOMOTORY_BEHAVIOR	0.011494
	GOBP_ANIMAL_ORGAN_FORMATION	0.011494
	GOBP_BIOLOGICAL_PROCESS_INVOLVED_IN_INTRASPECIES_INTERACTION_BETWEEN_ORGANISMS	0.011494
	GOBP_CALCIUM_ION_IMPORT	0.011494
	GOBP_CARDIAC_MUSCLE_CELL_ACTION_POTENTIAL	0.011494
	GOBP_CARDIAC_VENTRICLE_MORPHOGENESIS	0.011494

	GOBP_CATECHOL_CONTAINING_COMPOUND_METABOLIC_PROCES S	0.01 149 4
	GOBP_CATECHOLAMINE_SECRETION	0.01 149 4
	GOBP_CELL_COMMUNICATION_INVOLVED_IN_CARDIAC_CONDUC TION	0.01 149 4
	GOBP_CELLULAR_EXTRAVASATION	0.01 149 4
	GOBP_CELLULAR_RESPONSE_TO_ACID_CHEMICAL	0.01 149 4
	GOBP_COMPLEMENT_ACTIVATION	0.01 149 4
	GOBP_DOPAMINE_TRANSPORT	0.01 149 4
	GOBP_ENDODERM_FORMATION	0.01 149 4

	GOBP_ENERGY_RESERVE_METABOLIC_PROCESS	0.011494
	GOBP_FILOPODIUM_ASSEMBLY	0.011494
	GOBP_HEART_VALVE_DEVELOPMENT	0.011494
	GOBP_HEART_VALVE_MORPHOGENESIS	0.011494
	GOBP_NEGATIVE_REGULATION_OF_ACTIN_FILAMENT_POLYMERIZATION	0.011494
	GOBP_NEGATIVE_REGULATION_OF_AXONOGENESIS	0.011494
	GOBP_NEGATIVE_REGULATION_OF_BMP_SIGNALING_PATHWAY	0.011494
	GOBP_NEGATIVE_REGULATION_OF_CALCIUM_ION_TRANSPORT	0.011494

	GOBP_NEGATIVE_REGULATION_OF_EPITHELIAL_CELL_MIGRATION	0.011494
	GOBP_NEGATIVE_REGULATION_OF_MUSCLE_CELL_DIFFERENTIATION	0.011494
	GOBP_NEGATIVE_REGULATION_OF_PROTEIN_POLYMERIZATION	0.011494
	GOBP_NEGATIVE_REGULATION_OF_SMOOTH_MUSCLE_CELL_PROLIFERATION	0.011494
	GOBP_NEURON_PROJECTION_REGENERATION	0.011494
	GOBP_NON_CANONICAL_WNT_SIGNALING_PATHWAY	0.011494
	GOBP_PLATELET_AGGREGATION	0.011494
	GOBP_POSITIVE_CHEMOTAXIS	0.011494

	GOBP_POSITIVE_REGULATION_OF_AXONOGENESIS	0.01<br>149<br>4
	GOBP_POSITIVE_REGULATION_OF_LIPASE_ACTIVITY	0.01<br>149<br>4
	GOBP_POSITIVE_REGULATION_OF_MUSCLE_CELL_DIFFERENTIATI<br>ON	0.01<br>149<br>4
	GOBP_POSITIVE_REGULATION_OF_RECEPTOR_MEDIATED_ENDOC<br>YTOSIS	0.01<br>149<br>4
	GOBP_POSITIVE_REGULATION_OF_SMALL_GTPASE_MEDIATED_SI<br>GNAL_TRANSDUCTION	0.01<br>149<br>4
	GOBP_PROTEIN_LOCALIZATION_TO_CELL_SURFACE	0.01<br>149<br>4
	GOBP_RECEPTOR_CLUSTERING	0.01<br>149<br>4
	GOBP_REGULATION_OF_ACTION_POTENTIAL	0.01<br>149<br>4

	GOBP_REGULATION_OF_BEHAVIOR	0.011494
	GOBP_REGULATION_OF_COAGULATION	0.011494
	GOBP_REGULATION_OF_GLUCOSE_IMPORT	0.011494
	GOBP_REGULATION_OF_GLUCOSE_TRANSMEMBRANE_TRANSPORT	0.011494
	GOBP_REGULATION_OF_MORPHOGENESIS_OF_A_BRANCHING_STRUCTURE	0.011494
	GOBP_REGULATION_OF_MORPHOGENESIS_OF_AN_EPITHELIUM	0.011494
	GOBP_REGULATION_OF_MUSCLE_HYPERTROPHY	0.011494
	GOBP_REGULATION_OF_MYOBLAST_DIFFERENTIATION	0.011494

	GOBP_REGULATION_OF_PHOSPHOLIPASE_ACTIVITY	0.011494
	GOBP_REGULATION_OF_RELEASE_OF_SEQUESTERED_CALCIUM_ION_INTO_CYTOSOL	0.011494
	GOBP_REGULATION_OF_VASOCONSTRICTION	0.011494
	GOBP_RESPIRATORY_GASEOUS_EXCHANGE_BY_RESPIRATORY_SYSTEM	0.011494
	GOBP_SMOOTH_MUSCLE_CELL_MIGRATION	0.011494
	GOBP_STEM_CELL_PROLIFERATION	0.011494
	GOBP_STRIATED_MUSCLE_CELL_DEVELOPMENT	0.011494
	GOBP_SYNAPTIC_VESICLE_RECYCLING	0.011494

	GOBP_VASCULAR_ASSOCIATED_SMOOTH_MUSCLE_CELL_PROLIFERATION	0.011494
	GOCC_ACTIN_FILAMENT_BUNDLE	0.011494
	GOCC_INTRINSIC_COMPONENT_OF_POSTSYNAPTIC_SPECIALIZATION_MEMBRANE	0.011494
	GOCC_SCHAFFER_COLLATERAL_CA1_SYNAPSE	0.011494
	GOCC_T_TUBULE	0.011494
	GOMF_COPPER_ION_BINDING	0.011494
	GOMF_PEPTIDE_HORMONE_BINDING	0.011494
	GOMF_PHOSPHATIDYLINOSITOL_4_5_BISPHOSPHATE_BINDING	0.011494

	GOMF_PHOSPHATIDYLSERINE_BINDING	0.011494
	GOMF_TRANSMEMBRANE_RECEPTOR_PROTEIN_TYROSINE_KINASE_ACTIVITY	0.011494
	GOBP_CELL_ADHESION_MEDIATED_BY_INTEGRIN	0.011628
	GOBP_CELL_MIGRATION_INVOLVED_IN_SPROUTING_ANGIOGENESIS	0.011628
	GOBP_CRANIAL_NERVE_DEVELOPMENT	0.011628
	GOBP_DORSAL_VENTRAL_PATTERN_FORMATION	0.011628
	GOBP_ENDOTHELIAL_CELL_APOPTOTIC_PROCESS	0.011628
	GOBP_LONG_TERM_SYNAPTIC_POTENTIATION	0.011628

	GOBP_MEMBRANE_DEPOLARIZATION	0.011628
	GOBP_MUCOPOLYSACCHARIDE_METABOLIC_PROCESS	0.011628
	GOBP_MYOBLAST_DIFFERENTIATION	0.011628
	GOBP_NEGATIVE_REGULATION_OF_INTRACELLULAR_TRANSPORT	0.011628
	GOBP_OUTFLOW_TRACT_MORPHOGENESIS	0.011628
	GOBP_PLATELET_DERIVED_GROWTH_FACTOR_RECEPTOR_SIGNALING_PATHWAY	0.011628
	GOBP_POSITIVE_REGULATION_OF_SYNAPSE_ASSEMBLY	0.011628
	GOBP_REGULATION_OF_SYNAPTIC_TRANSMISSION_GLUTAMATERGIC	0.011628

	GOBP_TRANSMISSION_OF_NERVE_IMPULSE	0.011628
	GOBP_VASOCONSTRICTION	0.011628
	GOCC_CELL_CELL_CONTACT_ZONE	0.011628
	GOCC_NEUROMUSCULAR_JUNCTION	0.011628
	GOMF_AMYLOID_BETA_BINDING	0.011628
	GOBP_CARDIAC_MUSCLE_CELL_ACTION_POTENTIAL_INVOLVED_IN_CONTRACTION	0.01765
	GOBP_ENDOCARDIAL_CUSHION_DEVELOPMENT	0.01765
	GOBP_EPITHELIAL_CELL_DIFFERENTIATION_INVOLVED_IN_KIDNEY_DEVELOPMENT	0.01765

	GOBP_LUNG_MORPHOGENESIS	0.011765
	GOBP_POSITIVE_REGULATION_OF_BLOOD_VESSEL_ENDOTHELIAL_CELL_MIGRATION	0.011765
	GOBP_PRESYNAPSE_ORGANIZATION	0.011765
	GOBP_REGULATION_OF_CYCLASE_ACTIVITY	0.011765
	GOBP_RESPONSE_TO_AMYLOID_BETA	0.011765
	GOCC_INTERCALATED_DISC	0.011765
	GOMF_EXTRACELLULAR_MATRIX_BINDING	0.011765
	GOBP_NEGATIVE_REGULATION_OF_COAGULATION	0.012048

	GOBP_NEGATIVE_REGULATION_OF_FAT_CELL_DIFFERENTIATION	0.012048
	GOBP_POSITIVE_REGULATION_OF_EXTRINSIC_APOPTOTIC_SIGNALING_PATHWAY	0.012048
	GOBP_POSITIVE_REGULATION_OF_NEURAL_PRECURSOR_CELL_PROLIFERATION	0.012048
	GOBP_POSITIVE_REGULATION_OF_STRESS_FIBER_ASSEMBLY	0.012048
	GOBP_REGULATION_OF_LYASE_ACTIVITY	0.012048
	GOBP_VASCULAR_ENDOTHELIAL_GROWTH_FACTOR_RECEPTOR_SIGNALING_PATHWAY	0.012048
	GOCC_CALCIUM_CHANNEL_COMPLEX	0.012048
	GOCC_NEURON_PROJECTION_MEMBRANE	0.012048

	GOBP_ADHERENS_JUNCTION_ORGANIZATION	0.012195
	GOBP_DIGESTIVE_TRACT_MORPHOGENESIS	0.012195
	GOBP_ENDOCRINE_HORMONE_SECRETION	0.012195
	GOBP_FACE_DEVELOPMENT	0.012195
	GOBP_MACROPHAGE_MIGRATION	0.012195
	GOBP_NEURON_RECOGNITION	0.012195
	GOBP_REGULATION_OF_NEURONAL_SYNAPTIC_PLASTICITY	0.012195
	GOBP_RUFFLE_ORGANIZATION	0.012195

	GOBP_SYNAPTIC_VESICLE_LOCALIZATION	0.01<br>219<br>5
	GOMF_POTASSIUM_CHANNEL_REGULATOR_ACTIVITY	0.01<br>219<br>5
	GOBP_APOPTOTIC_CELL_CLEARANCE	0.01<br>234<br>6
	GOBP_AUTONOMIC_NERVOUS_SYSTEM_DEVELOPMENT	0.01<br>234<br>6
	GOBP_BROWN_FAT_CELL_DIFFERENTIATION	0.01<br>234<br>6
	GOBP_CAMP_MEDIATED_SIGNALING	0.01<br>234<br>6
	GOBP_EXTRACELLULAR_MATRIX_ASSEMBLY	0.01<br>234<br>6
	GOBP_MEMBRANE_REPOLARIZATION	0.01<br>234<br>6

	GOBP_NEGATIVE_CHEMOTAXIS	0.012346
	GOBP_NEGATIVE_REGULATION_OF_EPITHELIAL_CELL_APOPTOTIC_PROCESS	0.012346
	GOBP_OVULATION_CYCLE_PROCESS	0.012346
	GOBP_POSITIVE_REGULATION_OF_BIOMINERALIZATION	0.012346
	GOBP_POSITIVE_REGULATION_OF_EPITHELIAL_TO_MESENCHYMAL_TRANSITION	0.012346
	GOBP_POSITIVE_REGULATION_OF_OSSIFICATION	0.012346
	GOBP_POSITIVE_REGULATION_OF_PATHWAY_RESTRICTED_SMAD_PROTEIN_PHOSPHORYLATION	0.012346
	GOBP_REGULATION_OF_BONE_REMODELING	0.012346

	GOBP_REGULATION_OF_CELL_ADHESION_MEDIATED_BY_INTEGRIN	0.012346
	GOBP_REGULATION_OF_EXTRINSIC_APOPTOTIC_SIGNALING_PATHWAY_VIA_DEATH_DOMAIN_RECEPTORS	0.012346
	GOBP_REGULATION_OF_GRANULOCYTE_CHEMOTAXIS	0.012346
	GOBP_REGULATION_OF_MICROTUBULE_POLYMERIZATION	0.012346
	GOBP_VENTRICULAR_CARDIAC_MUSCLE_TISSUE_MORPHOGENESIS	0.012346
	GOCC_EXCITATORY_SYNAPSE	0.012346
	GOCC_INTRINSIC_COMPONENT_OF_POSTSYNAPTIC_DENSITY_MEMBRANE	0.012346
	GOMF_PROTEIN_KINASE_A_BINDING	0.012346
	GOBP_BODY_MORPHOGENESIS	0.0125

	GOBP_CARDIAC_MUSCLE_CELL_PROLIFERATION	0.0125
	GOBP_ENDODERMAL_CELL_DIFFERENTIATION	0.0125
	GOBP_EXOCRINE_SYSTEM_DEVELOPMENT	0.0125
	GOBP_NEUROTRANSMITTER_UPTAKE	0.0125
	GOBP_REGULATION_OF_GLYCOPROTEIN_METABOLIC_PROCESS	0.0125
	GOBP_REGULATION_OF_VASCULAR_PERMEABILITY	0.0125
	GOBP_SEMAPHORIN_PLEXIN_SIGNALING_PATHWAY	0.0125
	GOBP_TRABECULA_MORPHOGENESIS	0.0125
	GOMF_CORECEPTOR_ACTIVITY	0.0125
	GOBP_ACTIVIN_RECEPTOR_SIGNALING_PATHWAY	0.012821
	GOBP_AMYLOID_BETA_FORMATION	0.012821

	GOBP_BLOOD_VESSEL_REMODELING	0.012821
	GOBP_CELLULAR_RESPONSE_TO_AMYLOID_BETA	0.012821
	GOBP_CORONARY_VASCULATURE_DEVELOPMENT	0.012821
	GOBP_GLIAL_CELL_MIGRATION	0.012821
	GOBP_MESENCHYMAL_CELL_PROLIFERATION	0.012821
	GOBP_METAL_ION_EXPORT	0.012821
	GOBP_MITOCHONDRION_LOCALIZATION	0.012821
	GOBP_NEGATIVE_REGULATION_OF_OSTEOBLAST_DIFFERENTIATION	0.012821

	GOBP_NEUROMUSCULAR_JUNCTION_DEVELOPMENT	0.012821
	GOBP_NEURON_MATURATION	0.012821
	GOBP_PHENOL_CONTAINING_COMPOUND_BIOSYNTHETIC_PROCESS	0.012821
	GOBP_PITUITARY_GLAND_DEVELOPMENT	0.012821
	GOBP_POSITIVE_REGULATION_OF_SUBSTRATE_ADHESION_DEPENDENT_CELL_SPREADING	0.012821
	GOBP_REGULATION_OF_CHONDROCYTE_DIFFERENTIATION	0.012821
	GOBP_REGULATION_OF_LONG_TERM_SYNAPTIC_POTENTIATION	0.012821
	GOBP_REGULATION_OF_MUSCLE_ORGAN_DEVELOPMENT	0.012821

	GOBP_REGULATION_OF_NEUTROPHIL_MIGRATION	0.012821
	GOBP_REGULATION_OF_PHOSPHOLIPASE_C_ACTIVITY	0.012821
	GOBP_SEMI_LUNAR_VALVE_DEVELOPMENT	0.012821
	GOBP_VENTRAL_SPINAL_CORD_DEVELOPMENT	0.012821
	GOCC_PROTEIN_COMPLEX_INVOLVED_IN_CELL_ADHESION	0.012821
	GOMF_SODIUM_CHANNEL_ACTIVITY	0.012821
	GOMF_STRUCTURAL_CONSTITUENT_OF_MUSCLE	0.012821
	GOBP_ACROSOME_REACTION	0.013514

	GOBP_BRANCHING_INVOLVED_IN_BLOOD_VESSEL_MORPHOGENESIS	0.013514
	GOBP_CARDIAC_ATRIUM_DEVELOPMENT	0.013514
	GOBP_CARDIAC_MUSCLE_CELL_MEMBRANE_REPOLARIZATION	0.013514
	GOBP_CELLULAR_RESPONSE_TO_NUTRIENT	0.013514
	GOBP_COLLAGEN_BIOSYNTHETIC_PROCESS	0.013514
	GOBP_CYCLIC_NUCLEOTIDE_METABOLIC_PROCESS	0.013514
	GOBP_DETECTION_OF_MECHANICAL_STIMULUS	0.013514
	GOBP_DOPAMINE_RECEPTOR_SIGNALING_PATHWAY	0.013514

	GOBP_DOPAMINE_SECRETION	0.013514
	GOBP_EMBRYONIC_EYE_MORPHOGENESIS	0.013514
	GOBP_ENDOCARDIAL_CUSHION_MORPHOGENESIS	0.013514
	GOBP_INSULIN_LIKE_GROWTH_FACTOR_RECEPTOR_SIGNALING_PATHWAY	0.013514
	GOBP_MAINTENANCE_OF_BLOOD_BRAIN_BARRIER	0.013514
	GOBP_MEMBRANE_DEPOLARIZATION_DURING_ACTION_POTENTIAL	0.013514
	GOBP_MODULATION_OF_EXCITATORY_POSTSYNAPTIC_POTENTIAL	0.013514
	GOBP_NEGATIVE_REGULATION_OF_BLOOD_VESSEL_ENDOTHELIAL_CELL_MIGRATION	0.013514

	GOBP_NEGATIVE_REGULATION_OF_ENDOTHELIAL_CELL_PROLIFERATION	0.013514
	GOBP_NEGATIVE_REGULATION_OF_INTRACELLULAR_STEROID_HORMONE_RECEPTOR_SIGNALING_PATHWAY	0.013514
	GOBP_NEGATIVE_REGULATION_OF_MICROTUBULE_POLYMERIZATION_OR_DEPOLYMERIZATION	0.013514
	GOBP_NEURON_PROJECTION_EXTENSION_INVOLVED_IN_NEURON_PROJECTION_GUIDANCE	0.013514
	GOBP_NEUROTRANSMITTER_REUPTAKE	0.013514
	GOBP_NEUROTROPHIN_SIGNALING_PATHWAY	0.013514
	GOBP_POSITIVE_REGULATION_OF_ANIMAL_ORGAN_MORPHOGENESIS	0.013514
	GOBP_POSITIVE_REGULATION_OF_GLUCOSE_IMPORT	0.013514

	GOBP_POSITIVE_REGULATION_OF_GLUCOSE_TRANSMEMBRANE_TRANSPORT	0.013514
	GOBP_POSITIVE_REGULATION_OF_LIPID_KINASE_ACTIVITY	0.013514
	GOBP_POSITIVE_REGULATION_OF_MORPHOGENESIS_OF_AN_EPITHELIUM	0.013514
	GOBP_POSITIVE_REGULATION_OF_ORGAN_GROWTH	0.013514
	GOBP_POSTSYNAPTIC_MEMBRANE_ORGANIZATION	0.013514
	GOBP_POSTSYNAPTIC_SPECIALIZATION_ORGANIZATION	0.013514
	GOBP_REGULATION_OF_ACTIN_FILAMENT_BASED_MOVEMENT	0.013514
	GOBP_REGULATION_OF_CALCINEURIN_MEDIATED_SIGNALING	0.013514

	GOBP_REGULATION_OF_CARDIAC_MUSCLE_CELL_PROLIFERATION	0.013514
	GOBP_REGULATION_OF_ENDOCRINE_PROCESS	0.013514
	GOBP_REGULATION_OF_EXTRACELLULAR_MATRIX_ORGANIZATION	0.013514
	GOBP_REGULATION_OF_MYOTUBE_DIFFERENTIATION	0.013514
	GOBP_RESPIRATORY_SYSTEM_PROCESS	0.013514
	GOBP_RESPONSE_TO_ELECTRICAL_STIMULUS	0.013514
	GOBP_SALIVARY_GLAND_DEVELOPMENT	0.013514
	GOBP_SARCOMERE_ORGANIZATION	0.013514

	GOBP_SIGNALING_RECEPTOR_LIGAND_PRECURSOR_PROCESSING	0.01<br>351<br>4
	GOBP_SOMATIC_STEM_CELL_POPULATION_MAINTENANCE	0.01<br>351<br>4
	GOBP_SYNAPTIC_VESICLE_TRANSPORT	0.01<br>351<br>4
	GOBP_VASCULAR_ENDOTHELIAL_GROWTH_FACTOR_SIGNALING_<br>PATHWAY	0.01<br>351<br>4
	GOCC_DENDRITE_MEMBRANE	0.01<br>351<br>4
	GOCC_GTPASE_COMPLEX	0.01<br>351<br>4
	GOCC_SARCOPLASMIC_RETICULUM_MEMBRANE	0.01<br>351<br>4
	GOCC_VOLTAGE_GATED_CALCIUM_CHANNEL_COMPLEX	0.01<br>351<br>4

	GOMF_ACTININ_BINDING	0.01 351 4
	GOMF_CYCLIC_NUCLEOTIDE_BINDING	0.01 351 4
	GOMF_FRIZZLED_BINDING	0.01 351 4
	GOMF_GLUTAMATE_RECEPTOR_BINDING	0.01 351 4
	GOMF_PROTEOGLYCAN_BINDING	0.01 351 4
	GOMF_VOLTAGE_GATED_CALCIUM_CHANNEL_ACTIVITY	0.01 351 4
	GOBP_AMINE_BIOSYNTHETIC_PROCESS	0.01 369 9
	GOBP_ANIMAL_ORGAN_MATURATION	0.01 369 9

	GOBP_CALCIUM_DEPENDENT_CELL_CELL_ADHESION_VIA_PLASMA_MEMBRANE_CELL_ADHESION_MOLECULES	0.013699
	GOBP_CARDIAC_EPITHELIAL_TO_MESENCHYMAL_TRANSITION	0.013699
	GOBP_CELL_SURFACE_RECEPTOR_SIGNALING_PATHWAY_INVOLVED_IN_HEART_DEVELOPMENT	0.013699
	GOBP_DOPAMINE_METABOLIC_PROCESS	0.013699
	GOBP_GLUCAN_BIOSYNTHETIC_PROCESS	0.013699
	GOBP_HETEROPHILIC_CELL_CELL_ADHESION_VIA_PLASMA_MEMBRANE_CELL_ADHESION_MOLECULES	0.013699
	GOBP_METANEPHRIC_NEPHRON_DEVELOPMENT	0.013699
	GOBP_NEGATIVE_REGULATION_OF_CATION_CHANNEL_ACTIVITY	0.013699

	GOBP_NEGATIVE_REGULATION_OF_CELL_JUNCTION_ASSEMBLY	0.01369 9
	GOBP_NEGATIVE_REGULATION_OF_LEUKOCYTE_MIGRATION	0.01369 9
	GOBP_NEGATIVE_REGULATION_OF_POTASSIUM_ION_TRANSPORT	0.01369 9
	GOBP_OLIGODENDROCYTE_DEVELOPMENT	0.01369 9
	GOBP_OSTEOBLAST_PROLIFERATION	0.01369 9
	GOBP_POSITIVE_REGULATION_OF_AMINE_TRANSPORT	0.01369 9
	GOBP_POSITIVE_REGULATION_OF_AXON_EXTENSION	0.01369 9
	GOBP_POSITIVE_REGULATION_OF_BONE_MINERALIZATION	0.01369 9

	GOBP_POSITIVE_REGULATION_OF_NEUTROPHIL_MIGRATION	0.013699
	GOBP_POSITIVE_REGULATION_OF_STRIATED_MUSCLE_CELL_DIFFERENTIATION	0.013699
	GOBP_POSTSYNAPSE_ASSEMBLY	0.013699
	GOBP_PROTEIN_KINASE_A_SIGNALING	0.013699
	GOBP_REGULATION_OF_AMINO_ACID_TRANSPORT	0.013699
	GOBP_REGULATION_OF_CELL_MIGRATION_INVOLVED_IN_SPROUTING_ANGIOGENESIS	0.013699
	GOBP_REGULATION_OF_CELLULAR_EXTRAVASATION	0.013699
	GOBP_REGULATION_OF_FIBROBLAST_GROWTH_FACTOR_RECEPTOR_SIGNALING_PATHWAY	0.013699

	GOBP_REGULATION_OF_FILOPODIUM_ASSEMBLY	0.013699
	GOBP_REGULATION_OF_GLYCOGEN_METABOLIC_PROCESS	0.013699
	GOBP_REGULATION_OF_HEART_RATE_BY_CARDIAC_CONDUCTION	0.013699
	GOBP_REGULATION_OF_PROTEIN_AUTOPHOSPHORYLATION	0.013699
	GOBP_RENAL_SYSTEM_VASCULATURE_DEVELOPMENT	0.013699
	GOBP_SPECIFICATION_OF_ANIMAL_ORGAN_IDENTITY	0.013699
	GOBP_SUBSTANTIA_NIGRA_DEVELOPMENT	0.013699
	GOBP_VENTRICULAR_SEPTUM_MORPHOGENESIS	0.013699

	GOCC_CILIARY_TIP	0.01<br>369<br>9
	GOMF_BETA_TUBULIN_BINDING	0.01<br>369<br>9
	GOMF_CALCIUM_CHANNEL_REGULATOR_ACTIVITY	0.01<br>369<br>9
	GOMF_EXTRACELLULAR_MATRIX_STRUCTURAL_CONSTITUENT_CONFERRING_TENSILE_STRENGTH	0.01<br>369<br>9
	GOMF_PEPTIDASE_ACTIVATOR_ACTIVITY	0.01<br>369<br>9
	GOBP_ACTIVATION_OF_PHOSPHOLIPASE_C_ACTIVITY	0.01<br>408<br>5
	GOBP_AORTIC_VALVE_MORPHOGENESIS	0.01<br>408<br>5
	GOBP_BASEMENT_MEMBRANE_ORGANIZATION	0.01<br>408<br>5

	GOBP_BONE_GROWTH	0.014085
	GOBP_CELL_CELL_SIGNALING_INVOLVED_IN_CARDIAC_CONDUCTION	0.014085
	GOBP_CELL_COMMUNICATION_BY_ELECTRICAL_COUPLING	0.014085
	GOBP_CELLULAR_RESPONSE_TO_VITAMIN	0.014085
	GOBP_CENTRAL_NERVOUS_SYSTEM_NEURON_AXONOGENESIS	0.014085
	GOBP_CGMP_MEDIATED_SIGNALING	0.014085
	GOBP_CHONDROCYTE_DEVELOPMENT	0.014085
	GOBP_CHONDROITIN_SULFATE_PROTEOGLYCAN_METABOLIC_PROCESS	0.014085

	GOBP_DOPAMINERGIC_NEURON_DIFFERENTIATION	0.014085
	GOBP_EMBRYONIC_DIGESTIVE_TRACT_DEVELOPMENT	0.014085
	GOBP_ENDOCARDIAL_CUSHION_FORMATION	0.014085
	GOBP_EPIDERMIS_MORPHOGENESIS	0.014085
	GOBP_HEART_FORMATION	0.014085
	GOBP_MATERNAL_PLACENTA_DEVELOPMENT	0.014085
	GOBP_MYOTUBE_CELL_DEVELOPMENT	0.014085
	GOBP_NEGATIVE_REGULATION_OF_CALCIUM_ION_TRANSMEMBRANE_TRANSPORT	0.014085

	GOBP_NEGATIVE_REGULATION_OF_CELL_MATRIX_ADHESION	0.01<br>408<br>5
	GOBP_NEGATIVE_REGULATION_OF_ENDOTHELIAL_CELL_APOPTO<br>TIC_PROCESS	0.01<br>408<br>5
	GOBP_NEGATIVE_REGULATION_OF_EPITHELIAL_TO_MESENCHYM<br>AL_TRANSITION	0.01<br>408<br>5
	GOBP_NEGATIVE_REGULATION_OF_OSSIFICATION	0.01<br>408<br>5
	GOBP_NEGATIVE_REGULATION_OF_SMOOTHENED_SIGNALING_PA<br>THWAY	0.01<br>408<br>5
	GOBP_PIGMENT_CELL_DIFFERENTIATION	0.01<br>408<br>5
	GOBP_POSITIVE_REGULATION_OF_CALCIUM_ION_TRANSMEMBRA<br>NE_TRANSPORTER_ACTIVITY	0.01<br>408<br>5
	GOBP_POSITIVE_REGULATION_OF_CELL_SUBSTRATE_JUNCTION_O<br>RGANIZATION	0.01<br>408<br>5

	GOBP_POSITIVE_REGULATION_OF_HEART_GROWTH	0.014085
	GOBP_POSITIVE_REGULATION_OF_NERVOUS_SYSTEM_PROCESS	0.014085
	GOBP_PURINERGIC_NUCLEOTIDE_RECEPTOR_SIGNALING_PATHWAY	0.014085
	GOBP_REGULATION_OF_ANIMAL_ORGAN_FORMATION	0.014085
	GOBP_REGULATION_OF_FIBROBLAST_MIGRATION	0.014085
	GOBP_REGULATION_OF_NEURON_PROJECTION_REGENERATION	0.014085
	GOBP_REGULATION_OF_NEUTROPHIL_CHEMOTAXIS	0.014085
	GOBP_REGULATION_OF_PRESYNAPSE_ORGANIZATION	0.014085

	GOBP_REGULATION_OF_RENAL_SYSTEM_PROCESS	0.01<br>408<br>5
	GOBP_REGULATION_OF_VOLTAGE_GATED_CALCIUM_CHANNEL_A<br>CTIVITY	0.01<br>408<br>5
	GOBP_RELAXATION_OF_MUSCLE	0.01<br>408<br>5
	GOBP_RELEASE_OF_SEQUESTERED_CALCIUM_ION_INTO_CYTOSOL<br>_BY_ENDOPLASMIC_RETICULUM	0.01<br>408<br>5
	GOBP_RESPONSE_TO_FLUID_SHEAR_STRESS	0.01<br>408<br>5
	GOBP_RESPONSE_TO_ISOQUINOLINE_ALKALOID	0.01<br>408<br>5
	GOBP_RESPONSE_TO_PROSTAGLANDIN	0.01<br>408<br>5
	GOBP_RESPONSE_TO_VITAMIN_D	0.01<br>408<br>5

	GOBP_SARCOPLASMIC_RETICULUM_CALCIUM_ION_TRANSPORT	0.01 408 5
	GOCC_DENDRITIC_SHAFT	0.01 408 5
	GOCC_INTEGRIN_COMPLEX	0.01 408 5
	GOCC_PRESYNAPTIC_ACTIVE_ZONE_MEMBRANE	0.01 408 5
	GOMF_FIBRONECTIN_BINDING	0.01 408 5
	GOMF_HORMONE_RECEPTOR_BINDING	0.01 408 5
	GOBP_AORTA_MORPHOGENESIS	0.01 428 6
	GOBP_HEAD_MORPHOGENESIS	0.01 428 6

	GOBP_HINDLIMB_MORPHOGENESIS	0.01 428 6
	GOBP_NEGATIVE_REGULATION_OF_PROTEIN_LOCALIZATION_TO_NUCLEUS	0.01 428 6
	GOBP_NEGATIVE_REGULATION_OF_STRIATED_MUSCLE_CELL_DIFFERENTIATION	0.01 428 6
	GOBP_POSITIVE_REGULATION_OF_CELLULAR_RESPONSE_TO_TRANSFORMING_GROWTH_FACTOR_BETA_STIMULUS	0.01 428 6
	GOBP_POSITIVE_REGULATION_OF_SMOOTH_MUSCLE_CELL_MIGRATION	0.01 428 6
	GOBP_POSITIVE_REGULATION_OF_SMOOTHENED_SIGNALING_PATHWAY	0.01 428 6
	GOBP_REGULATION_OF_ADENYLATE_CYCLASE_ACTIVITY	0.01 428 6
	GOBP_REGULATION_OF_KIDNEY_DEVELOPMENT	0.01 428 6

	GOBP_REGULATION_OF_MEMBRANE_REPOLARIZATION	0.014286
	GOCC_A_BAND	0.014286
	GOBP_ATRIOVENTRICULAR_VALVE_DEVELOPMENT	0.014493
	GOBP_BONE_MATURATION	0.014493
	GOBP_EMBRYONIC_HINDLIMB_MORPHOGENESIS	0.014493
	GOBP_POSITIVE_REGULATION_OF_FOCAL_ADHESION_ASSEMBLY	0.014493
	GOBP_REGULATION_OF_ACTIVIN_RECEPTOR_SIGNALING_PATHWAY	0.014493
	GOBP_REGULATION_OF_CARDIAC_CONDUCTION	0.014493

	GOBP_REGULATION_OF_NON_CANONICAL_WNT_SIGNALING_PATHWAY	0.014493
	GOBP_RENAL_TUBULAR_SECRETION	0.014493
	GOBP_SYNAPTIC_MEMBRANE_ADHESION	0.014493
	GOMF_ALPHA_ACTININ_BINDING	0.014493
	GOMF_VOLTAGE_GATED_SODIUM_CHANNEL_ACTIVITY	0.014493
	GOBP_ADRENAL_GLAND_DEVELOPMENT	0.014706
	GOBP_CARDIAC_ATRIUM_MORPHOGENESIS	0.014706
	GOBP_ENDOTHELIAL_CELL_CHEMOTAXIS	0.014706

	GOBP_HEART_TRABECULA_MORPHOGENESIS	0.01<br>470<br>6
	GOBP_LYMPH_VESSEL_DEVELOPMENT	0.01<br>470<br>6
	GOBP_NEGATIVE_REGULATION_OF_OSTEOCLAST_DIFFERENTIATI<br>ON	0.01<br>470<br>6
	GOBP_NEURON_PROJECTION_ARBORIZATION	0.01<br>470<br>6
	GOBP_POSITIVE_REGULATION_OF_SYNAPTIC_TRANSMISSION_GLU<br>TAMATERGIC	0.01<br>470<br>6
	GOBP_REGULATION_OF_SMOOTH_MUSCLE_CELL_DIFFERENTIATIO<br>N	0.01<br>470<br>6
	GOBP_RESPONSE_TO_ACETYLCHOLINE	0.01<br>470<br>6
	GOBP_STEM_CELL_DIVISION	0.01<br>470<br>6

	GOMF_LIPOPROTEIN_PARTICLE_RECEPTOR_BINDING	0.01<br>470<br>6
	GOMF_NEUROPEPTIDE_HORMONE_ACTIVITY	0.01<br>470<br>6
	GOBP_ADENYLATE_CYCLASE_ACTIVATING_ADRENERGIC_RECEPT<br>OR_SIGNALING_PATHWAY	0.01<br>492<br>5
	GOBP_AXIS_ELONGATION	0.01<br>492<br>5
	GOBP_CELL_COMMUNICATION_BY_ELECTRICAL_COUPLING_INVO<br>LVED_IN_CARDIAC_CONDUCTION	0.01<br>492<br>5
	GOBP_CELL_DIFFERENTIATION_INVOLVED_IN_METANEPHROS_DE<br>VELOPMENT	0.01<br>492<br>5
	GOBP_ESTABLISHMENT_OF_MITOCHONDRION_LOCALIZATION	0.01<br>492<br>5
	GOBP_FIBRINOLYSIS	0.01<br>492<br>5

	GOBP_GAMMA_AMINOBUTYRIC_ACID_SIGNALING_PATHWAY	0.014925
	GOBP_GLOMERULAR_EPITHELIAL_CELL_DIFFERENTIATION	0.014925
	GOBP_GLOMERULAR_EPITHELIUM_DEVELOPMENT	0.014925
	GOBP_INNERVATION	0.014925
	GOBP_MELANOCYTE_DIFFERENTIATION	0.014925
	GOBP_MOTOR_NEURON_AXON_GUIDANCE	0.014925
	GOBP_NEGATIVE_REGULATION_OF_AMINE_TRANSPORT	0.014925
	GOBP_NEGATIVE_REGULATION_OF_CARTILAGE_DEVELOPMENT	0.014925

	GOBP_NEGATIVE_REGULATION_OF_OXIDOREDUCTASE_ACTIVITY	0.014925
	GOBP_NEUROMUSCULAR_SYNAPTIC_TRANSMISSION	0.014925
	GOBP_OUTFLOW_TRACT_SEPTUM_MORPHOGENESIS	0.014925
	GOBP_PHAGOCYTOSIS_RECOGNITION	0.014925
	GOBP_PHARYNGEAL_SYSTEM_DEVELOPMENT	0.014925
	GOBP_POSITIVE_REGULATION_OF_EXCITATORY_POSTSYNAPTIC_POTENTIAL	0.014925
	GOBP_POSITIVE_REGULATION_OF_MUSCLE_HYPERTROPHY	0.014925
	GOBP_REGULATION_OF_CARDIAC_MUSCLE_CELL_MEMBRANE_REPOLARIZATION	0.014925

	GOBP_REGULATION_OF_CARDIOCYTE_DIFFERENTIATION	0.014925
	GOBP_REGULATION_OF_GLUCAN_BIOSYNTHETIC_PROCESS	0.014925
	GOBP_REGULATION_OF_OSTEOBLAST_PROLIFERATION	0.014925
	GOBP_REGULATION_OF_THE_FORCE_OF_HEART_CONTRACTION	0.014925
	GOBP_RESPONSE_TO_PAIN	0.014925
	GOBP_RESPONSE_TO_PROSTAGLANDIN_E	0.014925
	GOBP_SECONDARY_PALATE_DEVELOPMENT	0.014925
	GOBP_SUBSTRATE_DEPENDENT_CELL_MIGRATION	0.014925

	GOBP_TRABECULA_FORMATION	0.014925
	GOBP_VENTRICULAR_CARDIAC_MUSCLE_CELL_MEMBRANE_REPOLARIZATION	0.014925
	GOCC_ACROSOMAL_MEMBRANE	0.014925
	GOCC_ATPASE_DEPENDENT_TRANSMEMBRANE_TRANSPORT_COMPLEX	0.014925
	GOCC_DENSE_CORE_GRANULE	0.014925
	GOCC_SODIUM_CHANNEL_COMPLEX	0.014925
	GOMF_CYCLIC_NUCLEOTIDE_PHOSPHODIESTERASE_ACTIVITY	0.014925
	GOMF_FIBROBLAST_GROWTH_FACTOR_RECEPTOR_BINDING	0.014925

	GOMF_INSULIN_LIKE_GROWTH_FACTOR_BINDING	0.01 492 5
	GOMF_WNT_PROTEIN_BINDING	0.01 492 5
	GOMF_WW_DOMAIN_BINDING	0.01 492 5
	GOBP_BASEMENT_MEMBRANE_ASSEMBLY	0.01 515 2
	GOBP_CARDIAC_LEFT_VENTRICLE_MORPHOGENESIS	0.01 515 2
	GOBP_CELLULAR_POTASSIUM_ION_HOMEOSTASIS	0.01 515 2
	GOBP_CELLULAR_RESPONSE_TO_FOLLICLE_STIMULATING_HORMONE_STIMULUS	0.01 515 2
	GOBP_CELLULAR_RESPONSE_TO_PROSTAGLANDIN_E_STIMULUS	0.01 515 2

	GOBP_CELLULAR_RESPONSE_TO_THYROID_HORMONE_STIMULUS	0.01 515 2
	GOBP_COMPLEMENT_ACTIVATION_LECTIN_PATHWAY	0.01 515 2
	GOBP_CYCLIC_NUCLEOTIDE_CATABOLIC_PROCESS	0.01 515 2
	GOBP_DENDRITIC_CELL_ANTIGEN_PROCESSING_AND_PRESENTATION	0.01 515 2
	GOBP_DETECTION_OF_MECHANICAL_STIMULUS_INVOLVED_IN_SENSORY_PERCEPTION_OF_PAIN	0.01 515 2
	GOBP_DORSAL_SPINAL_CORD_DEVELOPMENT	0.01 515 2
	GOBP_GANGLIOSIDE_BIOSYNTHETIC_PROCESS	0.01 515 2
	GOBP_HEART_TRABECULA_FORMATION	0.01 515 2

	GOBP_INDUCTION_OF_POSITIVE_CHEMOTAXIS	0.015152
	GOBP_INHIBITORY_POSTSYNAPTIC_POTENTIAL	0.015152
	GOBP_LUTEINIZATION	0.015152
	GOBP_MIDBRAIN_DOPAMINERGIC_NEURON_DIFFERENTIATION	0.015152
	GOBP_MODIFICATION_OF_POSTSYNAPTIC_STRUCTURE	0.015152
	GOBP_MYOBLAST_MIGRATION	0.015152
	GOBP_NEGATIVE_REGULATION_OF_ANDROGEN_RECEPTOR_SIGNALING_PATHWAY	0.015152
	GOBP_NEGATIVE_REGULATION_OF_LEUKOCYTE_CHEMOTAXIS	0.015152

	GOBP_NEGATIVE_REGULATION_OF_MUSCLE_TISSUE_DEVELOPMENT	0.015152
	GOBP_NEGATIVE_REGULATION_OF_NITRIC_OXIDE_METABOLIC_PROCESS	0.015152
	GOBP_NEGATIVE_REGULATION_OF_PLATELET_DERIVED_GROWTH_FACTOR_RECEPTOR_SIGNALING_PATHWAY	0.015152
	GOBP_NEGATIVE_REGULATION_OF_VASCULAR_PERMEABILITY	0.015152
	GOBP_NEUROTRANSMITTER_GATED_ION_CHANNEL_CLUSTERING	0.015152
	GOBP_NOREPINEPHRINE_METABOLIC_PROCESS	0.015152
	GOBP_OLFACTORY_BULB_INTERNEURON_DIFFERENTIATION	0.015152
	GOBP_OPSONIZATION	0.015152

	GOBP_PERIPHERAL_NERVOUS_SYSTEM_NEURON_DIFFERENTIATION	0.015152
	GOBP_POSITIVE_REGULATION_OF_CALCIUM_ION_DEPENDENT_EXOCYTOSIS	0.015152
	GOBP_POSITIVE_REGULATION_OF_CATECHOLAMINE_SECRETION	0.015152
	GOBP_POSITIVE_REGULATION_OF_ENDOTHELIAL_CELL_CHEMOTAXIS	0.015152
	GOBP_POSITIVE_REGULATION_OF_NEUROTRANSMITTER_SECRETION	0.015152
	GOBP_REGULATION_OF_ADENYLATE_CYCLASE_ACTIVATING_G_PROTEIN_COUPLED_RECEPTOR_SIGNALING_PATHWAY	0.015152
	GOBP_REGULATION_OF_INCLUSION_BODY_ASSEMBLY	0.015152
	GOBP_REGULATION_OF_METALLOPEPTIDASE_ACTIVITY	0.015152

	GOBP_REGULATION_OF_RESPIRATORY_SYSTEM_PROCESS	0.01<br>515<br>2
	GOBP_REGULATION_OF_SKELETAL_MUSCLE_CELL_DIFFERENTIATION	0.01<br>515<br>2
	GOBP_RESPONSE_TO_INACTIVITY	0.01<br>515<br>2
	GOBP_SEMAPHORIN_PLEXIN_SIGNALING_PATHWAY_INVOLVED_IN_NEURON_PROJECTION_GUIDANCE	0.01<br>515<br>2
	GOBP_SUBSTRATE_INDEPENDENT_TELENCEPHALIC_TANGENTIAL_MIGRATION	0.01<br>515<br>2
	GOBP_SYNAPTIC_VESICLE_CLUSTERING	0.01<br>515<br>2
	GOBP_TRIGLYCERIDE_RICH_LIPOPROTEIN_PARTICLE_REMODELING	0.01<br>515<br>2
	GOBP_VENOUS_BLOOD_VESSEL_DEVELOPMENT	0.01<br>515<br>2

	GOBP_VENTRAL_SPINAL_CORD_INTERNEURON_DIFFERENTIATION	0.015152
	GOBP_VENTRICULAR_CARDIAC_MUSCLE_CELL_DIFFERENTIATION	0.015152
	GOCC_CHYLOMICRON	0.015152
	GOCC_EARLY_PHAGOSOME	0.015152
	GOCC_FIBRILLAR_COLLAGEN_TRIMER	0.015152
	GOCC_MICROFIBRIL	0.015152
	GOCC_MUSCLE_MYOSIN_COMPLEX	0.015152
	GOCC_NODE_OF_RANVIER	0.015152

	GOCC_PLATELET_ALPHA_GRANULE_MEMBRANE	0.01 515 2
	GOMF_FILAMIN_BINDING	0.01 515 2
	GOMF_NEUREXIN_FAMILY_PROTEIN_BINDING	0.01 515 2
	GOMF_TRANSFORMING_GROWTH_FACTOR_BETA_ACTIVATED_RECEPTOR_ACTIVITY	0.01 515 2
	GOMF_TROPOMYOSIN_BINDING	0.01 515 2
	GOBP_ACTIVATION_OF_TRANSMEMBRANE_RECEPTOR_PROTEIN_TYROSINE_KINASE_ACTIVITY	0.01 538 5
	GOBP_BEHAVIORAL_RESPONSE_TO_PAIN	0.01 538 5
	GOBP_CARDIAC_NEURAL_CREST_CELL_DEVELOPMENT_INVOLVED_IN_OUTFLOW_TRACT_MORPHOGENESIS	0.01 538 5

	GOBP_CARDIAC_NEURAL_CREST_CELL_MIGRATION_INVOLVED_IN_OUTFLOW_TRACT_MORPHOGENESIS	0.015385
	GOBP_CDC42_PROTEIN_SIGNAL_TRANSDUCTION	0.015385
	GOBP_CELLULAR_RESPONSE_TO_X_RAY	0.015385
	GOBP_DETECTION_OF_CALCIUM_ION	0.015385
	GOBP_ELASTIC_FIBER_ASSEMBLY	0.015385
	GOBP_EPINEPHRINE_TRANSPORT	0.015385
	GOBP_ESTABLISHMENT_OF_PLANAR_POLARITY_INVOLVED_IN_NEURAL_TUBE_CLOSURE	0.015385
	GOBP_EXTRACELLULAR_REGULATION_OF_SIGNAL_TRANSDUCTION	0.015385

	GOBP_G_PROTEIN_COUPLED_PURINERGIC_NUCLEOTIDE_RECEPTOR_SIGNALING_PATHWAY	0.015385
	GOBP_GLOMERULAR_BASEMENT_MEMBRANE_DEVELOPMENT	0.015385
	GOBP_INNATE_IMMUNE_RESPONSE_IN_MUCOSA	0.015385
	GOBP_MINERALOCORTICOID_METABOLIC_PROCESS	0.015385
	GOBP_NEGATIVE_REGULATION_OF_CALCINEURIN_MEDIATED_SIGNALING	0.015385
	GOBP_NEGATIVE_REGULATION_OF_CALCIUM_ION_TRANSPORT_INTO_CYTOSOL	0.015385
	GOBP_NEGATIVE_REGULATION_OF_CATECHOLAMINE_SECRETION	0.015385
	GOBP_NEGATIVE_REGULATION_OF_HOMOTYPIC_CELL_CELL_ADHESION	0.015385

	GOBP_NEGATIVE_REGULATION_OF_LIPASE_ACTIVITY	0.01<br>538<br>5
	GOBP_NEGATIVE_REGULATION_OF_LONG_TERM_SYNAPTIC_POTE<br>NTIATION	0.01<br>538<br>5
	GOBP_NEGATIVE_REGULATION_OF_MICROTUBULE_POLYMERIZAT<br>ION	0.01<br>538<br>5
	GOBP_NEGATIVE_REGULATION_OF_MONOOXYGENASE_ACTIVITY	0.01<br>538<br>5
	GOBP_NEGATIVE_REGULATION_OF_PROTEIN_IMPORT_INTO_NUCL<br>EUS	0.01<br>538<br>5
	GOBP_NEGATIVE_REGULATION_OF_SYNAPSE_ORGANIZATION	0.01<br>538<br>5
	GOBP_NEPHRIC_DUCT_DEVELOPMENT	0.01<br>538<br>5
	GOBP_NEPHRIC_DUCT_MORPHOGENESIS	0.01<br>538<br>5

	GOBP_NOTCH_SIGNALING_INVOLVED_IN_HEART_DEVELOPMENT	0.015385
	GOBP_PENILE_ERECTION	0.015385
	GOBP_PLANAR_CELL_POLARITY_PATHWAY_INVOLVED_IN_NEURAL_TUBE_CLOSURE	0.015385
	GOBP_POSITIVE_REGULATION_OF_ACTIN_NUCLEATION	0.015385
	GOBP_POSITIVE_REGULATION_OF_FIBROBLAST_MIGRATION	0.015385
	GOBP_POSITIVE_REGULATION_OF_INSULIN_LIKE_GROWTH_FACTOR_RECEPTOR_SIGNALING_PATHWAY	0.015385
	GOBP_POSITIVE_REGULATION_OF_SEQUESTERING_OF_CALCIUM_ION	0.015385
	GOBP_PRESYNAPTIC_MEMBRANE_ORGANIZATION	0.015385

	GOBP_PROTEIN_OXIDATION	0.01<br>538<br>5
	GOBP_REGULATION_OF_AXON_GUIDANCE	0.01<br>538<br>5
	GOBP_REGULATION_OF_CARDIAC_EPITHELIAL_TO_MESENCHYMAL_TRANSITION	0.01<br>538<br>5
	GOBP_REGULATION_OF_CELL_FATE_SPECIFICATION	0.01<br>538<br>5
	GOBP_REGULATION_OF_DOPAMINE_RECEPTOR_SIGNALING_PATHWAY	0.01<br>538<br>5
	GOBP_REGULATION_OF_GONADOTROPIN_SECRETION	0.01<br>538<br>5
	GOBP_REGULATION_OF_HEPATOCYTE_PROLIFERATION	0.01<br>538<br>5
	GOBP_REGULATION_OF_HIGH_VOLTAGE_GATED_CALCIUM_CHANNEL_ACTIVITY	0.01<br>538<br>5

	GOBP_REGULATION_OF_NEUROTROPHIN_TRK_RECEPTOR_SIGNALING_PATHWAY	0.015385
	GOBP_REGULATION_OF_ODONTOGENESIS_OF_DENTIN_CONTAINING_TOOTH	0.015385
	GOBP_REGULATION_OF_PEROXISOME_PROLIFERATOR_ACTIVATED_RECEPTOR_SIGNALING_PATHWAY	0.015385
	GOBP_REGULATION_OF_RESPIRATORY_GASEOUS_EXCHANGE_BY_NERVOUS_SYSTEM_PROCESS	0.015385
	GOBP_REGULATION_OF_STEM_CELL_DIVISION	0.015385
	GOBP_RESPONSE_TO_STIMULUS_INVOLVED_IN_REGULATION_OF_MUSCLE_ADAPTATION	0.015385
	GOBP_RETINA_VASCULATURE_MORPHOGENESIS_IN_CAMERA_TYPE_EYE	0.015385
	GOBP_SA_NODE_CELL_TO_ATRIAL_CARDIAC_MUSCLE_CELL_COMMUNICATION	0.015385

	GOBP_SEROTONIN_UPTAKE	0.01 538 5
	GOBP_VASCULAR_ASSOCIATED_SMOOTH_MUSCLE_CELL_DEVELOPMENT	0.01 538 5
	GOBP_VENTRICULAR_TRABECULA_MYOCARDIUM_MORPHOGENESIS	0.01 538 5
	GOCC_CHROMAFFIN_GRANULE	0.01 538 5
	GOCC_INTEGRAL_COMPONENT_OF_PRESYNAPTIC_ACTIVE_ZONE_MEMBRANE	0.01 538 5
	GOCC_INTERSTITIAL_MATRIX	0.01 538 5
	GOCC_L_TYPE_VOLTAGE_GATED_CALCIUM_CHANNEL_COMPLEX	0.01 538 5
	GOCC_NEURONAL_DENSE_CORE_VESICLE	0.01 538 5

	GOCC_SCAR_COMPLEX	0.01<br>538<br>5
	GOMF_CALCIUM_ACTIVATED_POTASSIUM_CHANNEL_ACTIVITY	0.01<br>538<br>5
	GOMF_G_PROTEIN_COUPLED_PURINERGIC_NUCLEOTIDE_RECEPTO<br>R_ACTIVITY	0.01<br>538<br>5
	GOMF_INSULIN_LIKE_GROWTH_FACTOR_I_BINDING	0.01<br>538<br>5
	GOMF_OUTWARD_RECTIFIER_POTASSIUM_CHANNEL_ACTIVITY	0.01<br>538<br>5
	GOMF_PLATELET_DERIVED_GROWTH_FACTOR_BINDING	0.01<br>538<br>5
	GOMF_STRUCTURAL_MOLECULE_ACTIVITY_CONFERRING_ELASTI<br>CITY	0.01<br>538<br>5
	GOBP_APICAL_PROTEIN_LOCALIZATION	0.01<br>562<br>5

	GOBP_CALCIUM_ION_TRANSMEMBRANE_TRANSPORT_VIA_HIGH_VOLTAGE_GATED_CALCIUM_CHANNEL	0.015625
	GOBP_CARDIAC_CHAMBER_FORMATION	0.015625
	GOBP_CGMP_METABOLIC_PROCESS	0.015625
	GOBP_CHAPERONE_MEDIATED_AUTOPHAGY	0.015625
	GOBP_COLLAGEN_ACTIVATED_SIGNALING_PATHWAY	0.015625
	GOBP_CONVERGENT_EXTENSION	0.015625
	GOBP_ENTERIC_NERVOUS_SYSTEM_DEVELOPMENT	0.015625
	GOBP_G_PROTEIN_COUPLED_RECEPTOR_SIGNALING_PATHWAY_INVOLVED_IN_HEART_PROCESS	0.015625

	GOBP_MORPHOGENESIS_OF_AN_EPITHELIAL_BUD	0.015625
	GOBP_NEGATIVE_REGULATION_OF_JUN_KINASE_ACTIVITY	0.015625
	GOBP_NEGATIVE_REGULATION_OF_NEURON_PROJECTION_REGENERATION	0.015625
	GOBP_PARTURITION	0.015625
	GOBP_PLATELET_DERIVED_GROWTH_FACTOR_RECEPTOR_BETA_SIGNALING_PATHWAY	0.015625
	GOBP_POSITIVE_REGULATION_OF_EXTRINSIC_APOPTOTIC_SIGNALING_PATHWAY_VIA_DEATH_DOMAIN_RECEPTORS	0.015625
	GOBP_POSITIVE_REGULATION_OF_GLYCOGEN_METABOLIC_PROCESS	0.015625
	GOBP_POSITIVE_REGULATION_OF_NON_CANONICAL_WNT_SIGNALING_PATHWAY	0.015625

	GOBP_REGULATION_OF_CARDIAC_MUSCLE_CELL_DIFFERENTIATION	0.015625
	GOBP_REGULATION_OF_CELL_GROWTH_INVOLVED_IN_CARDIAC_MUSCLE_CELL_DEVELOPMENT	0.015625
	GOBP_REGULATION_OF_COLLATERAL_SPROUTING	0.015625
	GOBP_REGULATION_OF_EXTRACELLULAR_MATRIX_ASSEMBLY	0.015625
	GOBP_REGULATION_OF_INTEGRIN_MEDIATED_SIGNALING_PATHWAY	0.015625
	GOBP_SEGMENT_SPECIFICATION	0.015625
	GOBP_STORE_OPERATED_CALCIUM_ENTRY	0.015625
	GOBP_WHITE_FAT_CELL_DIFFERENTIATION	0.015625

	GOCC_COSTAMERE	0.01<br>562<br>5
	GOCC_DYNACTIN_COMPLEX	0.01<br>562<br>5
	GOMF_3_5_CYCLIC_AMP_PHOSPHODIESTERASE_ACTIVITY	0.01<br>562<br>5
	GOMF_3_5_CYCLIC_GMP_PHOSPHODIESTERASE_ACTIVITY	0.01<br>562<br>5
	GOMF_ACTIVIN_BINDING	0.01<br>562<br>5
	GOMF_BMP_BINDING	0.01<br>562<br>5
	GOMF_HMG_BOX_DOMAIN_BINDING	0.01<br>562<br>5
	GOMF_INHIBITORY_EXTRACELLULAR_LIGAND_GATED_ION_CHAN<br>NEL_ACTIVITY	0.01<br>562<br>5

	GOMF_MUSCLE_ALPHA_ACTININ_BINDING	0.015625
	GOMF_OXIDOREDUCTASE_ACTIVITY_ACTING_ON_THE_CH_NH2_GROUP_OF_DONORS_OXYGEN_AS_ACCEPTOR	0.015625
	GOBP_CALCIUM_ION_IMPORT_INTO_CYTOSOL	0.015873
	GOBP_CAMP_METABOLIC_PROCESS	0.015873
	GOBP_CARDIAC_RIGHT_VENTRICLE_MORPHOGENESIS	0.015873
	GOBP_CATECHOL_CONTAINING_COMPOUND_BIOSYNTHETIC_PROCESS	0.015873
	GOBP_CELL_MIGRATION_INVOLVED_IN_HEART_DEVELOPMENT	0.015873
	GOBP_CENTRAL_NERVOUS_SYSTEM_PROJECTION_NEURON_AXONOGENESIS	0.015873

	GOBP_CHONDROITIN_SULFATE_PROTEOGLYCAN_BIOSYNTHETIC_PROCESS	0.015873
	GOBP_COLLATERAL_SPROUTING	0.015873
	GOBP_CYTOPLASMIC_SEQUESTERING_OF_PROTEIN	0.015873
	GOBP_EPITHELIAL_TO_MESENCHYMAL_TRANSITION_INVOLVED_IN_ENDOCARDIAL_CUSHION_FORMATION	0.015873
	GOBP_FOREBRAIN_NEURON_DEVELOPMENT	0.015873
	GOBP_GLUTAMATE_SECRETION	0.015873
	GOBP_INCLUSION_BODY_ASSEMBLY	0.015873
	GOBP_NEGATIVE_REGULATION_OF_ADENYLATE_CYCLASE_ACTIVITY	0.015873

	GOBP_NEGATIVE_REGULATION_OF_AMYLOID_PRECURSOR_PROTEIN_CATABOLIC_PROCESS	0.015873
	GOBP_NEGATIVE_REGULATION_OF_POTASSIUM_ION_TRANSMEMBRANE_TRANSPORT	0.015873
	GOBP_NEGATIVE_REGULATION_OF_SMOOTH_MUSCLE_CELL_MIGRATION	0.015873
	GOBP_NEGATIVE_REGULATION_OF_VASCULAR_ASSOCIATED_SMOOTH_MUSCLE_CELL_PROLIFERATION	0.015873
	GOBP_OVULATION	0.015873
	GOBP_PHYSIOLOGICAL_CARDIAC_MUSCLE_HYPERTROPHY	0.015873
	GOBP_POSITIVE_REGULATION_OF_EXTRACELLULAR_MATRIX_ORGANIZATION	0.015873
	GOBP_POSITIVE_REGULATION_OF_FILOPODIUM_ASSEMBLY	0.015873

	GOBP_POSITIVE_REGULATION_OF_MYOBLAST_DIFFERENTIATION	0.015873
	GOBP_POSTSYNAPTIC_SPECIALIZATION_ASSEMBLY	0.015873
	GOBP_REGULATION_OF_AMPA_RECEPTOR_ACTIVITY	0.015873
	GOBP_REGULATION_OF_APPETITE	0.015873
	GOBP_REGULATION_OF_CARDIAC_MUSCLE_CONTRACTION_BY_CALCIUM_ION_SIGNALING	0.015873
	GOBP_REGULATION_OF_FEEDING_BEHAVIOR	0.015873
	GOBP_REGULATION_OF_LONG_TERM_NEURONAL_SYNAPTIC_PLASTICITY	0.015873
	GOBP_REGULATION_OF_RESPIRATORY_GASEOUS_EXCHANGE	0.015873

	GOBP_REGULATION_OF_VASCULAR_ENDOTHELIAL_GROWTH_FACTOR_RECEPTOR_SIGNALING_PATHWAY	0.015873
	GOBP_REGULATION_OF_VESICLE_FUSION	0.015873
	GOBP_RENAL_SODIUM_EXCRETION	0.015873
	GOBP_SMOOTH_MUSCLE_TISSUE_DEVELOPMENT	0.015873
	GOBP_SYMPATHETIC_NERVOUS_SYSTEM_DEVELOPMENT	0.015873
	GOBP_VASCULAR_ENDOTHELIAL_CELL_PROLIFERATION	0.015873
	GOCC_GABA_RECEPTOR_COMPLEX	0.015873
	GOCC_GLYCOPROTEIN_COMPLEX	0.015873

	GOCC_M_BAND	0.01<br>587<br>3
	GOCC_MYOFILAMENT	0.01<br>587<br>3
	GOCC_MYOSIN_FILAMENT	0.01<br>587<br>3
	GOCC_MYOSIN_II_COMPLEX	0.01<br>587<br>3
	GOMF_CAMP_BINDING	0.01<br>587<br>3
	GOMF_EXTRACELLULAR_MATRIX_STRUCTURAL_CONSTITUENT_C<br>ONFERRING_COMPRESSION_RESISTANCE	0.01<br>587<br>3
	GOMF_G_PROTEIN_BETA_GAMMA_SUBUNIT_COMPLEX_BINDING	0.01<br>587<br>3
	GOMF_SIALYLTRANSFERASE_ACTIVITY	0.01<br>587<br>3

	GOBP_ATRIAL_SEPTUM_DEVELOPMENT	0.016129
	GOBP_AXON_ENSHEATHMENT_IN_CENTRAL_NERVOUS_SYSTEM	0.016129
	GOBP_AXONAL_FASCICULATION	0.016129
	GOBP_BLOOD_VESSEL_ENDOTHELIAL_CELL_PROLIFERATION_INVOLVED_IN_SPROUTING_ANGIOGENESIS	0.016129
	GOBP_BRANCHING_INVOLVED_IN_SALIVARY_GLAND_MORPHOGENESIS	0.016129
	GOBP_CARDIAC_MYOFIBRIL_ASSEMBLY	0.016129
	GOBP_CELL_AGGREGATION	0.016129
	GOBP_CELL_JUNCTION_DISASSEMBLY	0.016129

	GOBP_CELLULAR_RESPONSE_TO_PROSTAGLANDIN_STIMULUS	0.016129
	GOBP_CELLULAR_RESPONSE_TO_VITAMIN_D	0.016129
	GOBP_CHONDROCYTE_PROLIFERATION	0.016129
	GOBP_COPULATION	0.016129
	GOBP_DETECTION_OF_STIMULUS_INVOLVED_IN_SENSORY_PERCEPTION_OF_PAIN	0.016129
	GOBP_GROWTH_PLATE_CARTILAGE_DEVELOPMENT	0.016129
	GOBP_LIVER_MORPHOGENESIS	0.016129
	GOBP_LYMPH_VESSEL_MORPHOGENESIS	0.016129

	GOBP_MEMBRANE_DEPOLARIZATION_DURING_CARDIAC_MUSCLE_CELL_ACTION_POTENTIAL	0.016129
	GOBP_MIDDLE_EAR_MORPHOGENESIS	0.016129
	GOBP_MODIFICATION_OF_SYNAPTIC_STRUCTURE	0.016129
	GOBP_MORPHOGENESIS_OF_AN_EPITHELIAL_FOLD	0.016129
	GOBP_NEGATIVE_REGULATION_OF_ANION_TRANSPORT	0.016129
	GOBP_NEGATIVE_REGULATION_OF_CARDIAC_MUSCLE_TISSUE_GROWTH	0.016129
	GOBP_NEGATIVE_REGULATION_OF_MUSCLE_CONTRACTION	0.016129
	GOBP_NEGATIVE_REGULATION_OF_NUCLEOCYTOPLASMIC_TRANSPORT	0.016129

	GOBP_NEGATIVE_REGULATION_OF_ORGANIC_ACID_TRANSPORT	0.016129
	GOBP_NEGATIVE_REGULATION_OF_TISSUE_REMODELING	0.016129
	GOBP_NITRIC_OXIDE_MEDIATED_SIGNAL_TRANSDUCTION	0.016129
	GOBP_NOREPINEPHRINE_TRANSPORT	0.016129
	GOBP_PLASMINOGEN_ACTIVATION	0.016129
	GOBP_POSITIVE_REGULATION_OF_BEHAVIOR	0.016129
	GOBP_POSITIVE_REGULATION_OF_CARDIAC_MUSCLE_CELL_PROLIFERATION	0.016129
	GOBP_POSITIVE_REGULATION_OF_CELL_MIGRATION_INVOLVED_IN_SPROUTING_ANGIOGENESIS	0.016129

	GOBP_POSITIVE_REGULATION_OF_GLYCOPROTEIN_METABOLIC_PROCESS	0.016129
	GOBP_POSITIVE_REGULATION_OF_NEUROTRANSMITTER_TRANSPORT	0.016129
	GOBP_PULMONARY_VALVE_DEVELOPMENT	0.016129
	GOBP_REGULATION_OF_ANION_TRANSMEMBRANE_TRANSPORT	0.016129
	GOBP_REGULATION_OF_ANTIGEN_PROCESSING_AND_PRESENTATION	0.016129
	GOBP_REGULATION_OF_BRANCHING_INVOLVED_IN_URETERIC_BUD_MORPHOGENESIS	0.016129
	GOBP_REGULATION_OF_CELLULAR_RESPONSE_TO_VASCULAR_ENDOTHELIAL_GROWTH_FACTOR_STIMULUS	0.016129
	GOBP_REGULATION_OF_ENDOTHELIAL_CELL_CHEMOTAXIS	0.016129

	GOBP_REGULATION_OF_EXCRETION	0.016129
	GOBP_REGULATION_OF_INSULIN_LIKE_GROWTH_FACTOR_RECEPTOR_SIGNALING_PATHWAY	0.016129
	GOBP_REGULATION_OF_PLATELET_DERIVED_GROWTH_FACTOR_RECEPTOR_SIGNALING_PATHWAY	0.016129
	GOBP_REGULATION_OF_RECEPTOR_BINDING	0.016129
	GOBP_REGULATION_OF_RYANODINE_SENSITIVE_CALCIUM_RELEASE_CHANNEL_ACTIVITY	0.016129
	GOBP_REGULATION_OF_URINE_VOLUME	0.016129
	GOBP_RETINAL_GANGLION_CELL_AXON_GUIDANCE	0.016129
	GOBP_SEROTONIN_TRANSPORT	0.016129

	GOBP_STEROID_HORMONE_SECRETION	0.016129
	GOBP_VASCULAR_ASSOCIATED_SMOOTH_MUSCLE_CELL_MIGRATION	0.016129
	GOCC_AXONAL_GROWTH_CONE	0.016129
	GOCC_COMPLEX_OF_COLLAGEN_TRIMERS	0.016129
	GOCC_STRIATED_MUSCLE_THIN_FILAMENT	0.016129
	GOMF_GABA_RECEPTOR_ACTIVITY	0.016129
	GOMF_INSULIN_RECEPTOR_BINDING	0.016129
	GOMF_PHOSPHORUS_OXYGEN_LYASE_ACTIVITY	0.016129

	GOMF_RETINAL_BINDING	0.016129
	GOMF_STRUCTURAL_CONSTITUENT_OF_EYE_LENS	0.016129
	GOMF_TRANSFORMING_GROWTH_FACTOR_BETA_BINDING	0.016129
	GOBP_CARDIAC_NEURAL_CREST_CELL_DIFFERENTIATION_INVOLVED_IN_HEART_DEVELOPMENT	0.016393
	GOBP_CHONDROITIN_SULFATE_BIOSYNTHETIC_PROCESS	0.016393
	GOBP_EMBRYONIC_DIGESTIVE_TRACT_MORPHOGENESIS	0.016393
	GOBP_ESTABLISHMENT_OF_PLANAR_POLARITY_OF_EMBRYONIC_EPITHELIUM	0.016393
	GOBP_NEGATIVE_REGULATION_OF_CELL_SUBSTRATE_JUNCTION_ORGANIZATION	0.016393

	GOBP_PROTEIN_ACTIVATION_CASCADE	0.016393
	GOBP_REGULATION_OF_NEUROTRANSMITTER_UPTAKE	0.016393
	GOBP_REGULATION_OF_PROTEIN_KINASE_A_SIGNALING	0.016393
	GOBP_REGULATION_OF_WNT_SIGNALING_PATHWAY_PLANAR_CELL_POLARITY_PATHWAY	0.016393
	GOCC_INTRINSIC_COMPONENT_OF_PRESYNAPTIC_ACTIVE_ZONE_MEMBRANE	0.016393
	GOMF_METALLOENDOPEPTIDASE_INHIBITOR_ACTIVITY	0.016393
	GOBP_ATRIAL_CARDIAC_MUSCLE_CELL_TO_AV_NODE_CELL_SIGNALING	0.016667
	GOBP_GANGLION_DEVELOPMENT	0.016667

	GOBP_LYMPHANGIOGENESIS	0.01666 7
	GOBP_NEGATIVE_REGULATION_OF_BONE_REMODELING	0.01666 7
	GOBP_NEGATIVE_REGULATION_OF_CHONDROCYTE_DIFFERENTIATION	0.01666 7
	GOBP_NEGATIVE_REGULATION_OF_MONONUCLEAR_CELL_MIGRATION	0.01666 7
	GOBP_NEURON_CELL_CELL_ADHESION	0.01666 7
	GOBP_POSITIVE_REGULATION_OF_CELLULAR_EXTRAVASATION	0.01666 7
	GOBP_PULMONARY_VALVE_MORPHOGENESIS	0.01666 7
	GOBP_RELAXATION_OF_CARDIAC_MUSCLE	0.01666 7

	GOBP_TONGUE_DEVELOPMENT	0.016667
	GOBP_VOCALIZATION_BEHAVIOR	0.016667
	GOCC_CATION_TRANSPORTING_ATPASE_COMPLEX	0.016667
	GOCC_DYSTROPHIN_ASSOCIATED_GLYCOPROTEIN_COMPLEX	0.016667
	GOCC_VOLTAGE_GATED_SODIUM_CHANNEL_COMPLEX	0.016667
	GOMF_OXIDOREDUCTASE_ACTIVITY_ACTING_ON_THE_CH_NH2_GROUP_OF_DONORS	0.016667
	GOMF_TRANSMEMBRANE_RECEPTOR_PROTEIN_SERINE_THREONINE_KINASE_ACTIVITY	0.016667
	GOBP_BONE_MINERALIZATION_INVOLVED_IN_BONE_MATURATION	0.016949

	GOBP_CARDIAC_MUSCLE_CELL_MYOBLAST_DIFFERENTIATION	0.01<br>694<br>9
	GOBP_CARDIAC_VENTRICLE_FORMATION	0.01<br>694<br>9
	GOBP_CARTILAGE_MORPHOGENESIS	0.01<br>694<br>9
	GOBP_CORONARY_VASCULATURE_MORPHOGENESIS	0.01<br>694<br>9
	GOBP_CRANIAL_NERVE_STRUCTURAL_ORGANIZATION	0.01<br>694<br>9
	GOBP_DEFINITIVE_HEMOPOIESIS	0.01<br>694<br>9
	GOBP_DITERPENOID_BIOSYNTHETIC_PROCESS	0.01<br>694<br>9
	GOBP_DORSAL_AORTA_DEVELOPMENT	0.01<br>694<br>9

	GOBP_EMBRYONIC_BODY_MORPHOGENESIS	0.016949
	GOBP_FACIAL_NERVE_MORPHOGENESIS	0.016949
	GOBP_GASTRO_INTESTINAL_SYSTEM_SMOOTH_MUSCLE_CONTRACTION	0.016949
	GOBP_MEMBRANE_RAFT_ASSEMBLY	0.016949
	GOBP_MUSCLE_CELL_FATE_COMMITMENT	0.016949
	GOBP_NEGATIVE_REGULATION_OF_CARDIOCYTE_DIFFERENTIATION	0.016949
	GOBP_NEGATIVE_REGULATION_OF_FIBROBLAST_GROWTH_FACTOR_RECEPTOR_SIGNALING_PATHWAY	0.016949
	GOBP_NEUROTRANSMITTER_CATABOLIC_PROCESS	0.016949

	GOBP_PLASMA_MEMBRANE_FUSION	0.01694 9
	GOBP_POSITIVE_REGULATION_OF_CYTOPLASMIC_TRANSPORT	0.01694 9
	GOBP_POSITIVE_REGULATION_OF_MYOTUBE_DIFFERENTIATION	0.01694 9
	GOBP_REFLEX	0.01694 9
	GOBP_REGULATION_OF_CARDIAC_MUSCLE_CONTRACTION_BY_REGULATION_OF_THE_RELEASE_OF_SEQUESTERED_CALCIUM_ION	0.01694 9
	GOBP_REGULATION_OF_DENDRITIC_CELL_ANTIGEN_PROCESSING_AND_PRESENTATION	0.01694 9
	GOBP_REGULATION_OF_STEROID_HORMONE_SECRETION	0.01694 9
	GOBP_RESPONSE_TO_FOLLICLE_STIMULATING_HORMONE	0.01694 9

	GOBP_RETINA_VASCULATURE_DEVELOPMENT_IN_CAMERA_TYPE_EYE	0.016949
	GOBP_SPINAL_CORD_PATTERNING	0.016949
	GOBP_URETER_DEVELOPMENT	0.016949
	GOCC_FASCIA_ADHERENS	0.016949
	GOCC_PARANODE_REGION_OF_AXON	0.016949
	GOCC_SYMMETRIC_SYNAPSE	0.016949
	GOMF_ADENYLATE_CYCLASE_BINDING	0.016949
	GOMF_STRUCTURAL_CONSTITUENT_OF_POSTSYNAPSE	0.016949

	GOMF_TRANSFORMING_GROWTH_FACTOR_BETA_RECEPTOR_BINDING	0.016949
	GOBP_CELL_JUNCTION_ASSEMBLY	0.02
	GOBP_REGULATION_OF_CELLULAR_RESPONSE_TO_GROWTH_FACTOR_STIMULUS	0.02
	GOBP_REGULATION_OF_TRANS_SYNAPTIC_SIGNALING	0.02
	GOBP_SKELETAL_SYSTEM_DEVELOPMENT	0.02
	GOBP_CARDIAC_CONDUCTION	0.021277
	GOBP_EMBRYONIC_SKELETAL_SYSTEM_MORPHOGENESIS	0.021277
	GOBP_KIDNEY_MORPHOGENESIS	0.021277
	GOBP_POSITIVE_REGULATION_OF_ENDOCYTOSIS	0.021277
	GOBP_REGULATION_OF_ACTIN_FILAMENT_BUNDLE_ASSEMBLY	0.021277
	GOBP_REGULATION_OF_CELL_MORPHOGENESIS_INVOLVED_IN_DIFFERENTIATION	0.021277

	GOBP_REGULATION_OF_NEUROTRANSMITTER_TRANSPORT	0.021277
	GOBP_REGULATION_OF_SYNAPSE_ASSEMBLY	0.021277
	GOBP_RESPONSE_TO_CATECHOLAMINE	0.021277
	GOBP_SYNAPTIC_TRANSMISSION_GLUTAMATERGIC	0.021277
	GOCC_EXTRINSIC_COMPONENT_OF_CYTOPLASMIC_SIDE_OF_PLASMA_MEMBRANE	0.021277
	GOCC_FILOPODIUM	0.021277
	GOBP_POSITIVE_REGULATION_OF_CANONICAL_WNT_SIGNALING_PATHWAY	0.021505
	GOBP_RENAL_TUBULE_DEVELOPMENT	0.021505

	GOBP_SEGMENTATION	0.02<br>150<br>5
	GOBP_AXONAL_TRANSPORT	0.02<br>222<br>2
	GOBP_POSITIVE_REGULATION_OF_LEUKOCYTE_CHEMOTAXIS	0.02<br>222<br>2
	GOBP_ASSOCIATIVE_LEARNING	0.02<br>247<br>2
	GOBP_AXO_DENDRITIC_TRANSPORT	0.02<br>247<br>2
	GOBP_ENDODERM_DEVELOPMENT	0.02<br>247<br>2
	GOBP_EXTRACELLULAR_MATRIX_DISASSEMBLY	0.02<br>247<br>2
	GOBP_LAMELLIPODIUM_ORGANIZATION	0.02<br>247<br>2

	GOBP_NEGATIVE_REGULATION_OF_PROTEIN_CONTAINING_COMPLEX_DISASSEMBLY	0.022472
	GOBP_POSITIVE_REGULATION_OF_RESPONSE_TO_WOUNDING	0.022472
	GOBP_REGULATION_OF_INTRACELLULAR_STEROID_HORMONE_RECEPTOR_SIGNALING_PATHWAY	0.022472
	GOBP_REGULATION_OF_PROTEIN_DEPOLYMERIZATION	0.022472
	GOBP_REGULATION_OF_SODIUM_ION_TRANSPORT	0.022472
	GOMF_ALCOHOL_BINDING	0.022472
	GOBP_AMINOGLYCAN_BIOSYNTHETIC_PROCESS	0.022727
	GOBP_ASTROCYTE_DIFFERENTIATION	0.022727

	GOBP_FIBROBLAST_PROLIFERATION	0.02 272 7
	GOBP_NEGATIVE_REGULATION_OF_MYELOID_CELL_DIFFERENTIATION	0.02 272 7
	GOBP_ORGANELLE_TRANSPORT_ALONG_MICROTUBULE	0.02 272 7
	GOBP_POSITIVE_REGULATION_OF_CELL_DIVISION	0.02 272 7
	GOBP_REGULATION_OF_CALCIUM_MEDIATED_SIGNALING	0.02 272 7
	GOBP_REGULATION_OF_CARDIAC_MUSCLE_CONTRACTION	0.02 272 7
	GOBP_REGULATION_OF_SODIUM_ION_TRANSMEMBRANE_TRANSPORT	0.02 272 7
	GOBP_REGULATION_OF_STRIATED_MUSCLE_CONTRACTION	0.02 272 7

	GOCC_PRESYNAPTIC_ACTIVE_ZONE	0.022727
	GOMF_MYOSIN_BINDING	0.022727
	GOMF_PHOSPHATIDYLINOSITOL_BISPHOSPHATE_BINDING	0.022727
	GOMF_SCAFFOLD_PROTEIN_BINDING	0.022727
	GOBP_DENDRITIC_SPINE_MORPHOGENESIS	0.022989
	GOBP_EMBRYONIC_DIGIT_MORPHOGENESIS	0.022989
	GOBP_EMBRYONIC_HEART_TUBE_MORPHOGENESIS	0.022989
	GOBP_NEGATIVE_REGULATION_OF_SYNAPTIC_TRANSMISSION	0.022989

	GOBP_POSITIVE_REGULATION_OF_REPRODUCTIVE_PROCESS	0.022989
	GOBP_REGULATION_OF_NITRIC_OXIDE_METABOLIC_PROCESS	0.022989
	GOBP_ACTIVATION_OF_NF_KAPPAB_INDUCING_KINASE_ACTIVITY	0.023256
	GOBP_CELLULAR_RESPONSE_TO_ALCOHOL	0.023256
	GOBP_CLEAVAGE_INVOLVED_IN_RRNA_PROCESSING	0.023256
	GOBP_COLLAGEN_METABOLIC_PROCESS	0.023256
	GOBP_DENDRITIC_SPINE_DEVELOPMENT	0.023256
	GOBP_DOUBLE_STRAND_BREAK_REPAIR_VIA_BREAK_INDUCED_REPLICATION	0.023256

	GOBP_HISTONE_EXCHANGE	0.02<br>325<br>6
	GOBP_MANGANESE_ION_TRANSMEMBRANE_TRANSPORT	0.02<br>325<br>6
	GOBP_MEIOTIC_CELL_CYCLE_PHASE_TRANSITION	0.02<br>325<br>6
	GOBP_MITOCHONDRIAL_TRANSCRIPTION	0.02<br>325<br>6
	GOBP_NEGATIVE_REGULATION_OF_DOUBLE_STRAND_BREAK_REP<br>AIR_VIA_HOMOLOGOUS_RECOMBINATION	0.02<br>325<br>6
	GOBP_NEGATIVE_REGULATION_OF_UBIQUITIN_PROTEIN_TRANSFE<br>RASE_ACTIVITY	0.02<br>325<br>6
	GOBP_NEURON_PROJECTION_ORGANIZATION	0.02<br>325<br>6
	GOBP_NUCLEAR_MEMBRANE_REASSEMBLY	0.02<br>325<br>6

	GOBP_NUCLEAR_TRANSCRIBED_MRNA_CATABOLIC_PROCESS_EXONUCLEOLYTIC_3_5	0.023256
	GOBP_PENTOSE_METABOLIC_PROCESS	0.023256
	GOBP_POSITIVE_REGULATION_OF_MITOCHONDRIAL_TRANSLATION	0.023256
	GOBP_POSITIVE_REGULATION_OF_REGULATORY_T_CELL_DIFFERENTIATION	0.023256
	GOBP_POSITIVE_REGULATION_OF_TELOMERASE_RNA_LOCALIZATION_TO_CAJAL_BODY	0.023256
	GOBP_POSITIVE_REGULATION_OF_UBIQUITIN_PROTEIN_LIGASE_ACTIVITY	0.023256
	GOBP_PROTEIN_LOCALIZATION_TO_CHROMOSOME_CENTROMERIC_REGION	0.023256
	GOBP_PROTEIN_POLY_ADP_RIBOSYLATION	0.023256

	GOBP_PURINE_NUCLEOBASE_BIOSYNTHETIC_PROCESS	0.023256
	GOBP_RECEPTOR_LOCALIZATION_TO_SYNAPSE	0.023256
	GOBP_REGULATION_OF_CELL_PROJECTION_SIZE	0.023256
	GOBP_REGULATION_OF_MICROVILLUS_ORGANIZATION	0.023256
	GOBP_REGULATION_OF_MITOCHONDRIAL_GENE_EXPRESSION	0.023256
	GOBP_REGULATION_OF_MITOTIC_SPINDLE_ASSEMBLY	0.023256
	GOBP_REGULATION_OF_NK_T_CELL_ACTIVATION	0.023256
	GOBP_REGULATION_OF_NUCLEAR_CELL_CYCLE_DNA_REPLICATION	0.023256

	GOBP_REGULATION_OF_NUCLEASE_ACTIVITY	0.02<br>325<br>6
	GOBP_REGULATION_OF_RESPONSE_TO_TUMOR_CELL	0.02<br>325<br>6
	GOBP_REGULATION_OF_TRANSCRIPTION_OF_NUCLEOLAR_LARGE<br>_RRNA_BY_RNA_POLYMERASE_I	0.02<br>325<br>6
	GOBP_REGULATION_OF_VIRAL_INDUCED_CYTOPLASMIC_PATTER<br>N_RECOGNITION_RECEPTOR_SIGNALING_PATHWAY	0.02<br>325<br>6
	GOBP_RESPIRATORY_CHAIN_COMPLEX_IV_ASSEMBLY	0.02<br>325<br>6
	GOBP_RIBOSOMAL_LARGE_SUBUNIT_ASSEMBLY	0.02<br>325<br>6
	GOBP_RNA_INTERFERENCE	0.02<br>325<br>6
	GOBP_RRNA_CATABOLIC_PROCESS	0.02<br>325<br>6

	GOBP_SINGLE_STRAND_BREAK_REPAIR	0.023256
	GOBP_SNO_S_RNA_PROCESSING	0.023256
	GOBP_SPINDLE_MIDZONE_ASSEMBLY	0.023256
	GOBP_UMP_BIOSYNTHETIC_PROCESS	0.023256
	GOBP_UREA_CYCLE	0.023256
	GOCC_ANAPHASE_PROMOTING_COMPLEX	0.023256
	GOCC_DNA_REPLICATION_PREINITIATION_COMPLEX	0.023256
	GOCC_PRONUCLEUS	0.023256

	GOCC_PROTEASOME_CORE_COMPLEX	0.023256
	GOCC_PROTEASOME_CORE_COMPLEX_BETA_SUBUNIT_COMPLEX	0.023256
	GOMF_BETA_CATENIN_BINDING	0.023256
	GOMF_CATALYTIC_ACTIVITY_ACTING_ON_A_RRNA	0.023256
	GOMF_CYSTEINE_TYPE_ENDOPEPTIDASE_ACTIVITY_INVOLVED_IN_APOPTOTIC_SIGNALING_PATHWAY	0.023256
	GOMF_ENDODEOXYRIBONUCLEASE_ACTIVITY_PRODUCING_5_PHOSPHOMONOESTERS	0.023256
	GOMF_G_QUADRUPLEX_DNA_BINDING	0.023256
	GOMF_MANGANESE_ION_TRANSMEMBRANE_TRANSPORTER_ACTIVITY	0.023256

	GOBP_REGULATION_OF_MACROPHAGE_ACTIVATION	0.023529
	GOBP_REGULATION_OF_PHOSPHATIDYLINOSITOL_3_KINASE_ACTIVITY	0.023529
	GOBP_ANTIGEN_PROCESSING_AND_PRESENTATION_OF_ENDOGENOUS_PEPTIDE_ANTIGEN	0.02381
	GOBP_CELLULAR_RESPONSE_TO_REACTIVE_NITROGEN_SPECIES	0.02381
	GOBP_DOSAGE_COMPENSATION	0.02381
	GOBP_HISTONE_MRNA_METABOLIC_PROCESS	0.02381
	GOBP_IRE1_MEDIATED_UNFOLDED_PROTEIN_RESPONSE	0.02381
	GOBP_MATURATION_OF_5_8S_RRNA_FROM_TRICISTRONIC_RRNA_TRANSCRIPT_SSU_RRNA_5_8S_RRNA_LSU_RRNA	0.02381
	GOBP_MITOCHONDRIAL_RNA_PROCESSING	0.02381
	GOBP_MITOTIC_CHROMOSOME_CONDENSATION	0.02381
	GOBP_MITOTIC_SISTER_CHROMATID_COHESION	0.02381
	GOBP_MRNA_CLEAVAGE	0.02381

	GOBP_MRNA_MODIFICATION	0.02381
	GOBP_NEGATIVE_REGULATION_OF_MRNA_PROCESSING	0.02381
	GOBP_NUCLEAR_TRANSCRIBED_MRNA_CATABOLIC_PROCESS_EXONUCLEOLYTIC	0.02381
	GOBP_POSITIVE_REGULATION_OF_CHROMOSOME_SEPARATION	0.02381
	GOBP_REGULATION_OF_CD8_POSITIVE_ALPHA_BETA_T_CELL_ACTIVATION	0.02381
	GOBP_REGULATION_OF_EXOSOMAL_SECRETION	0.02381
	GOBP_RNA_5_END_PROCESSING	0.02381
	GOBP_RRNA_MODIFICATION	0.02381
	GOBP_SERINE_FAMILY_AMINO_ACID_BIOSYNTHETIC_PROCESS	0.02381
	GOBP_TETRAHYDROFOLATE_METABOLIC_PROCESS	0.02381
	GOCC_DNA_POLYMERASE_COMPLEX	0.02381
	GOMF_CADHERIN_BINDING_INVOLVED_IN_CELL_CELL_ADHESION	0.02381
	GOMF_EXODEOXYRIBONUCLEASE_ACTIVITY	0.02381

	GOMF_PROTEIN_ADP_RIBOSYLASE_ACTIVITY	0.02381
	GOMF_RIBOSOMAL_SMALL_SUBUNIT_BINDING	0.02381
	GOMF_SOLUTE_PROTON_ANTIPORTER_ACTIVITY	0.02381
	GOBP_CELL_DIFFERENTIATION_IN_SPINAL_CORD	0.02439
	GOBP_COENZYME_A_METABOLIC_PROCESS	0.02439
	GOBP_DNA_DAMAGE_RESPONSE_SIGNAL_TRANSDUCTION_BY_P53_CLASS_MEDIATOR_RESULTING_IN_CELL_CYCLE_ARREST	0.02439
	GOBP_EMBRYONIC_CRANIAL_SKELETON_MORPHOGENESIS	0.02439
	GOBP_FOREBRAIN_GENERATION_OF_NEURONS	0.02439
	GOBP_KINETOCHORE_ORGANIZATION	0.02439
	GOBP_MATURATION_OF_LSU_RRNA	0.02439
	GOBP_MITOTIC_INTRA_S_DNA_DAMAGE_CHECKPOINT_SIGNALING	0.02439
	GOBP_NEGATIVE_REGULATION_OF_TELOMERE_MAINTENANCE_VIA_TELOMERASE	0.02439
	GOBP_NUCLEOBASE_BIOSYNTHETIC_PROCESS	0.02439

	GOBP_NUCLEOSOME_MOBILIZATION	0.02439
	GOBP_OLIGOSACCHARIDE_LIPID_INTERMEDIATE_BIOSYNTHETIC_PROCESS	0.02439
	GOBP_POSITIVE_REGULATION_OF_CALCIUM_ION_TRANSPORT_INTO_CYTOSOL	0.02439
	GOBP_POSITIVE_REGULATION_OF_PROTEIN_TYROSINE_KINASE_ACTIVITY	0.02439
	GOBP_POSITIVE_REGULATION_OF_TELOMERE_CAPPING	0.02439
	GOBP_PURINE_NUCLEOBASE_METABOLIC_PROCESS	0.02439
	GOBP_PYROPTOSIS	0.02439
	GOBP_REGULATION_OF_CENTRIOLE_REPLICATION	0.02439
	GOBP_REGULATION_OF_CYSTEINE_TYPE_ENDOPEPTIDASE_ACTIVITY_INVOLVED_IN_APOPTOTIC_SIGNALING_PATHWAY	0.02439
	GOBP_REGULATION_OF_DENDRITIC_SPINE_DEVELOPMENT	0.02439
	GOBP_REGULATION_OF_MEMBRANE_DEPOLARIZATION	0.02439
	GOBP_REGULATION_OF_MITOTIC_CELL_CYCLE_SPINDLE_ASSEMBLY_CHECKPOINT	0.02439
	GOBP_REGULATION_OF_PLATELET_ACTIVATION	0.02439

	GOBP_REGULATION_OF_RIG_I_SIGNALING_PATHWAY	0.02439
	GOBP_REGULATION_OF_SISTER_CHROMATID_COHESION	0.02439
	GOBP_VASODILATION	0.02439
	GOCC_CUL4_RING_E3_UBIQUITIN_LIGASE_COMPLEX	0.02439
	GOCC_INFLAMMASOME_COMPLEX	0.02439
	GOCC_KINETOCHORE_MICROTUBULE	0.02439
	GOCC_OUTER_MITOCHONDRIAL_MEMBRANE_PROTEIN_COMPLEX	0.02439
	GOCC_PERICENTRIC_HETEROCHROMATIN	0.02439
	GOCC_PRERIBOSOME_LARGE_SUBUNIT_PRECURSOR	0.02439
	GOCC_TELOMERASE_HOLOENZYME_COMPLEX	0.02439
	GOMF_ARACHIDONIC_ACID_EPOXYGENASE_ACTIVITY	0.02439
	GOMF_C_ACYLTRANSFERASE_ACTIVITY	0.02439
	GOMF_CXCR_CHEMOKINE_RECEPTOR_BINDING	0.02439

	GOMF_DNA_POLYMERASE_BINDING	0.02<br>439
	GOMF_PLUS_END_DIRECTED_MICROTUBULE_MOTOR_ACTIVITY	0.02<br>439
	GOMF_PROTEIN_KINASE_C_BINDING	0.02<br>439
	GOMF_TELOMERASE_RNA_BINDING	0.02<br>439
	GOBP_NEGATIVE_REGULATION_OF_MYELOID_LEUKOCYTE_DIFFE<br>RENTIATION	0.02<br>469<br>1
	GOBP_POSITIVE_REGULATION_OF_FIBROBLAST_PROLIFERATION	0.02<br>469<br>1
	GOBP_POSITIVE_REGULATION_OF_POTASSIUM_ION_TRANSPORT	0.02<br>469<br>1
	GOMF_FATTY_ACID_BINDING	0.02<br>469<br>1
	GOBP_ACTIN_FILAMENT_BASED_TRANSPORT	0.02<br>5
	GOBP_ALDITOL_METABOLIC_PROCESS	0.02<br>5
	GOBP_AMP_METABOLIC_PROCESS	0.02<br>5

	GOBP_BRANCHED_CHAIN_AMINO_ACID_CATABOLIC_PROCESS	0.025
	GOBP_CALCINEURIN_MEDIATED_SIGNALING	0.025
	GOBP_CARBOHYDRATE_PHOSPHORYLATION	0.025
	GOBP_CENTROMERE_COMPLEX_ASSEMBLY	0.025
	GOBP_DIOL_METABOLIC_PROCESS	0.025
	GOBP_GLUCOSE_6_PHOSPHATE_METABOLIC_PROCESS	0.025
	GOBP_IRON_SULFUR_CLUSTER_ASSEMBLY	0.025
	GOBP_MEIOTIC_CHROMOSOME_SEPARATION	0.025
	GOBP_NEGATIVE_REGULATION_OF_NATURAL_KILLER_CELL_MEDIATED_IMMUNITY	0.025
	GOBP_POSITIVE_REGULATION_OF_MEIOTIC_CELL_CYCLE	0.025
	GOBP_POSITIVE_REGULATION_OF_TRANSCRIPTION_BY_RNA_POLYMERASE_I	0.025
	GOBP_PROTEIN_LOCALIZATION_TO_CHROMATIN	0.025
	GOBP_PURINE_NUCLEOSIDE_METABOLIC_PROCESS	0.025

	GOBP_PURINE_NUCLEOSIDE_MONOPHOSPHATE_BIOSYNTHETIC_PROCESS	0.025
	GOBP_PYRIMIDINE_DEOXYRIBONUCLEOTIDE_METABOLIC_PROCESS	0.025
	GOBP_PYRIMIDINE_NUCLEOSIDE_MONOPHOSPHATE_METABOLIC_PROCESS	0.025
	GOBP_REGULATION_OF_TRANSLATION_IN_RESPONSE_TO_STRESS	0.025
	GOBP_REGULATION_OF_UBIQUITIN_PROTEIN_LIGASE_ACTIVITY	0.025
	GOBP_REGULATION_OF_WATER_LOSS_VIA_SKIN	0.025
	GOBP_TRANSLESION_SYNTHESIS	0.025
	GOCC_EXORIBONUCLEASE_COMPLEX	0.025
	GOCC_INO80_TYPE_COMPLEX	0.025
	GOCC_PROTEASOME_REGULATORY_PARTICLE	0.025
	GOMF_ADENYLYLTRANSFERASE_ACTIVITY	0.025
	GOMF_ARACHIDONIC_ACID_MONOOXYGENASE_ACTIVITY	0.025
	GOMF_CARBOHYDRATE_KINASE_ACTIVITY	0.025

	GOMF_CATALYTIC_ACTIVITY_ACTING_ON_A_GLYCOPROTEIN	0.025
	GOMF_FATTY_ACID_LIGASE_ACTIVITY	0.025
	GOMF_PHOSPHOTRANSFERASE_ACTIVITY_FOR_OTHER_SUBSTITUTED_PHOSPHATE_GROUPS	0.025
	GOMF_TAU_PROTEIN_BINDING	0.025
	GOMF_TRNA_METHYLTRANSFERASE_ACTIVITY	0.025
	GOBP_CYTOPLASMIC_TRANSLATIONAL_INITIATION	0.025641
	GOBP_DEFENSE_RESPONSE_TO_FUNGUS	0.025641
	GOBP_DEOXYRIBONUCLEOSIDE_MONOPHOSPHATE_METABOLIC_PROCESS	0.025641
	GOBP_DNA_UNWINDING_INVOLVED_IN_DNA_REPLICATION	0.025641
	GOBP_ENDOPLASMIC_RETICULUM_TO_CYTOSOL_TRANSPORT	0.025641

	GOBP_ESTABLISHMENT_OF_PROTEIN_LOCALIZATION_TO_MITOCHONDRIAL_MEMBRANE	0.025641
	GOBP_IMMUNE_RESPONSE_TO_TUMOR_CELL	0.025641
	GOBP_INTERSTRAND_CROSS_LINK_REPAIR	0.025641
	GOBP_NEGATIVE_REGULATION_OF_TELOMERE_MAINTENANCE_VIA_TELOMERE_LENGTHENING	0.025641
	GOBP_NUCLEOSIDE_CATABOLIC_PROCESS	0.025641
	GOBP_POSITIVE_REGULATION_OF_INTERFERON_ALPHA_PRODUCTION	0.025641
	GOBP_PROTEIN_AUTOPROCESSING	0.025641
	GOBP_REGULATION_OF_PROTEIN_EXIT_FROM_ENDOPLASMIC_RETICULUM	0.025641

	GOBP_RESPONSE_TO_PROTOZOAN	0.02 564 1
	GOBP_SNRNA_PROCESSING	0.02 564 1
	GOBP_SODIUM_ION_IMPORT_ACROSS_PLASMA_MEMBRANE	0.02 564 1
	GOCC_90S_PRERIBOSOME	0.02 564 1
	GOCC_CORE_MEDIATOR_COMPLEX	0.02 564 1
	GOCC_INTRINSIC_COMPONENT_OF_SYNAPTIC_VESICLE_MEMBRA NE	0.02 564 1
	GOCC_MHC_PROTEIN_COMPLEX	0.02 564 1
	GOCC_PROTEASOME_ACCESSORY_COMPLEX	0.02 564 1

	GOCC_U12_TYPE_SPLICEOSOMAL_COMPLEX	0.025641
	GOMF_ACYL_COA_BINDING	0.025641
	GOMF_ACYLGLYCEROL_O_ACYLTRANSFERASE_ACTIVITY	0.025641
	GOMF_DNA_DIRECTED_DNA_POLYMERASE_ACTIVITY	0.025641
	GOMF_LIGASE_ACTIVITY_FORMING_CARBON_OXYGEN_BONDS	0.025641
	GOMF_PEPTIDE_LYSINE_N_ACETYLTRANSFERASE_ACTIVITY	0.025641
	GOMF_PEPTIDE_N_ACETYLTRANSFERASE_ACTIVITY	0.025641
	GOMF_SINGLE_STRANDED_DNA_HELICASE_ACTIVITY	0.025641

	GOMF_SNORNA_BINDING	0.025641
	GOMF_STRUCTURAL_CONSTITUENT_OF_NUCLEAR_PORE	0.025641
	GOBP_CELLULAR_RESPONSE_TO_STEROL_DEPLETION	0.026316
	GOBP_DNA_DOUBLE_STRAND_BREAK_PROCESSING	0.026316
	GOBP_DNA_LIGATION	0.026316
	GOBP_DNA_REPLICATION_INDEPENDENT_CHROMATIN_ORGANIZATION	0.026316
	GOBP_DNA_STRAND_ELONGATION_INVOLVED_IN_DNA_REPLICATION	0.026316
	GOBP_FUCOSE_METABOLIC_PROCESS	0.026316

	GOBP_G1_TO_G0_TRANSITION	0.026316
	GOBP_GLUTAMINE_FAMILY_AMINO_ACID_BIOSYNTHETIC_PROCESS	0.026316
	GOBP_HEPATOCYTE_DIFFERENTIATION	0.026316
	GOBP_POSITIVE_REGULATION_OF_CHROMOSOME_SEGREGATION	0.026316
	GOBP_PYRIMIDINE_NUCLEOSIDE_MONOPHOSPHATE_BIOSYNTHETIC_PROCESS	0.026316
	GOBP_REGULATION_OF_RESPONSE_TO_INTERFERON_GAMMA	0.026316
	GOBP_RNA_SURVEILLANCE	0.026316
	GOCC_REPLISOME	0.026316

	GOCC_SMN_SM_PROTEIN_COMPLEX	0.026316
	GOCC_SNO_S_RNA_CONTAINING_RIBONUCLEOPROTEIN_COMPLEX	0.026316
	GOBP_ATTACHMENT_OF_MITOTIC_SPINDLE_MICROTUBULES_TO_KINETOCHORE	0.027027
	GOBP_CENTROSOME_SEPARATION	0.027027
	GOBP_COENZYME_A_BIOSYNTHETIC_PROCESS	0.027027
	GOBP_DEOXYRIBONUCLEOSIDE_TRIPHOSPHATE_METABOLIC_PROCESS	0.027027
	GOBP_DNA_REPLICATION_CHECKPOINT_SIGNALING	0.027027
	GOBP_ENDONUCLEOLYTIC_CLEAVAGE_INVOLVED_IN_RRNA_PROCESSING	0.027027

	GOBP_FATTY_ACID_DERIVATIVE_CATABOLIC_PROCESS	0.027027
	GOBP_FRUCTOSE_METABOLIC_PROCESS	0.027027
	GOBP_GMP_BIOSYNTHETIC_PROCESS	0.027027
	GOBP_IMP_BIOSYNTHETIC_PROCESS	0.027027
	GOBP_INNER_CELL_MASS_CELL_PROLIFERATION	0.027027
	GOBP_INTRACELLULAR_TRANSPORT_OF_VIRUS	0.027027
	GOBP_KINETOCHORE_ASSEMBLY	0.027027
	GOBP_MEIOTIC_SPINDLE_ORGANIZATION	0.027027

	GOBP_MITOCHONDRIAL_DNA_METABOLIC_PROCESS	0.027027
	GOBP_MITOCHONDRIAL_DNA_REPLICATION	0.027027
	GOBP_NEGATIVE_REGULATION_OF_DNA_DEPENDENT_DNA_REPLICATION	0.027027
	GOBP_NEGATIVE_REGULATION_OF_MUSCLE_CELL_APOPTOTIC_PROCESS	0.027027
	GOBP_NUCLEOLAR_LARGE_RRNA_TRANSCRIPTION_BY_RNA_POLYMERASE_I	0.027027
	GOBP_NUCLEOSIDE_DIPHOSPHATE_BIOSYNTHETIC_PROCESS	0.027027
	GOBP_OLFACTORY_LOBE_DEVELOPMENT	0.027027
	GOBP_PENTOSE_PHOSPHATE_SHUNT	0.027027

	GOBP_POSITIVE_REGULATION_OF_BLOOD_PRESSURE	0.027027
	GOBP_POSITIVE_REGULATION_OF_PROTEIN_POLYUBIQUITINATION	0.027027
	GOBP_POSITIVE_REGULATION_OF_T_CELL_APOPTOTIC_PROCESS	0.027027
	GOBP_PROTEIN_IMPORT_INTO_MITOCHONDRIAL_MATRIX	0.027027
	GOBP_PROTEIN_IMPORT_INTO_PEROXISOME_MATRIX	0.027027
	GOBP_PROTEIN_LOCALIZATION_TO_CONDENSED_CHROMOSOME	0.027027
	GOBP_PROTEIN_LOCALIZATION_TO_POSTSYNAPSE	0.027027
	GOBP_PSEUDOURIDINE_SYNTHESIS	0.027027

	GOBP_REGULATION_OF_ENDOTHELIAL_CELL_DIFFERENTIATION	0.02 702 7
	GOBP_REGULATION_OF_RRNA_PROCESSING	0.02 702 7
	GOBP_REGULATION_OF_SPROUTING_ANGIOGENESIS	0.02 702 7
	GOBP_REGULATION_OF_TRANSLATIONAL_FIDELITY	0.02 702 7
	GOBP_RESPONSE_TO_UV_C	0.02 702 7
	GOBP_RIBOSOMAL_SUBUNIT_EXPORT_FROM_NUCLEUS	0.02 702 7
	GOBP_RRNA_METHYLATION	0.02 702 7
	GOBP_SNO_S_RNA_METABOLIC_PROCESS	0.02 702 7

	GOBP_TELOMERASE_RNA_LOCALIZATION	0.027027
	GOBP_TRNA_CATABOLIC_PROCESS	0.027027
	GOBP_TRNA_WOBBLE_BASE_MODIFICATION	0.027027
	GOBP_URONIC_ACID_METABOLIC_PROCESS	0.027027
	GOCC_CYTOLYTIC_GRANULE	0.027027
	GOCC_CYTOPLASMIC_EXOSOME_RNASE_COMPLEX	0.027027
	GOCC_EUKARYOTIC_TRANSLATION_INITIATION_FACTOR_3_COMPLEX	0.027027
	GOCC_LATERAL_ELEMENT	0.027027

	GOCC_PRERIBOSOME_SMALL_SUBUNIT_PRECURSOR	0.027027
	GOCC_PROTEIN_LIPID_COMPLEX	0.027027
	GOCC_ZYMOGEN_GRANULE_MEMBRANE	0.027027
	GOMF_3_5_DNA_HELICASE_ACTIVITY	0.027027
	GOMF_FUCOSYLTRANSFERASE_ACTIVITY	0.027027
	GOMF_OXIDIZED_DNA_BINDING	0.027027
	GOBP_NEGATIVE_REGULATION_OF_ORGAN_GROWTH	0.027397
	GOBP_POTASSIUM_ION_HOMEOSTASIS	0.027397

	GOBP_AMP_BIOSYNTHETIC_PROCESS	0.027778
	GOBP_ANAPHASE_PROMOTING_COMPLEX_DEPENDENT_CATABOLIC_PROCESS	0.027778
	GOBP_BASE_EXCISION_REPAIR_GAP_FILLING	0.027778
	GOBP_COBALAMIN_METABOLIC_PROCESS	0.027778
	GOBP_CRISTAE_FORMATION	0.027778
	GOBP_CYTOCHROME_COMPLEX_ASSEMBLY	0.027778
	GOBP_DEOXYRIBONUCLEOTIDE_BIOSYNTHETIC_PROCESS	0.027778
	GOBP_DNA_DAMAGE_RESPONSE_SIGNAL_TRANSDUCTION_RESULTING_IN_TRANSCRIPTION	0.027778

	GOBP_EXONUCLEOLYTIC_CATABOLISM_OF_DEADENYLATED_MRNA	0.027778
	GOBP_FORMATION_OF_EXTRACHROMOSOMAL_CIRCULAR_DNA	0.027778
	GOBP_FUCOSYLATION	0.027778
	GOBP_IMP_METABOLIC_PROCESS	0.027778
	GOBP_MHC_CLASS_II_BIOSYNTHETIC_PROCESS	0.027778
	GOBP_MIDBODY_ABSCISSION	0.027778
	GOBP_MITOTIC_DNA_REPLICATION	0.027778
	GOBP_MRNA_CLEAVAGE_INVOLVED_IN_MRNA_PROCESSING	0.027778

	GOBP_NEGATIVE_REGULATION_OF_CELL_DIVISION	0.027778
	GOBP_NEGATIVE_REGULATION_OF_TYPE_I_INTERFERON_MEDIATED_SIGNALING_PATHWAY	0.027778
	GOBP_NK_T_CELL_ACTIVATION	0.027778
	GOBP_NUCLEAR_PORE_ORGANIZATION	0.027778
	GOBP_POSITIVE_REGULATION_OF_DNA_DEPENDENT_DNA_REPLICATION	0.027778
	GOBP_POSITIVE_REGULATION_OF_EXOSOMAL_SECRETION	0.027778
	GOBP_POSITIVE_REGULATION_OF_KERATINOCYTE_DIFFERENTIATION	0.027778
	GOBP_POSITIVE_REGULATION_OF_METAPHASE_ANAPHASE_TRANSITION_OF_CELL_CYCLE	0.027778

	GOBP_POSITIVE_REGULATION_OF_RESPONSE_TO_TUMOR_CELL	0.027778
	GOBP_PROTEIN_LOCALIZATION_TO_NUCLEOLUS	0.027778
	GOBP_PTERIDINE_CONTAINING_COMPOUND_BIOSYNTHETIC_PROCESS	0.027778
	GOBP_REGULATION_OF_ATTACHMENT_OF_SPINDLE_MICROTUBULES_TO_KINETOCHORE	0.027778
	GOBP_REGULATION_OF_DNA_DIRECTED_DNA_POLYMERASE_ACTIVITY	0.027778
	GOBP_REGULATION_OF_IRE1_MEDIATED_UNFOLDED_PROTEIN_RESPONSE	0.027778
	GOBP_RESPONSE_TO_STEROL_DEPLETION	0.027778
	GOBP_TRNA_5_END_PROCESSING	0.027778

	GOBP_UBIQUINONE_METABOLIC_PROCESS	0.02777 8
	GOBP_UREA_METABOLIC_PROCESS	0.02777 8
	GOCC_CCR4_NOT_COMPLEX	0.02777 8
	GOCC_CHROMOCENTER	0.02777 8
	GOCC_INTRINSIC_COMPONENT_OF_PEROXISOMAL_MEMBRANE	0.02777 8
	GOCC_METHYLOSOME	0.02777 8
	GOCC_MRNA_CLEAVAGE_AND_POLYADENYLATION_SPECIFICITY_ FACTOR_COMPLEX	0.02777 8
	GOCC_MRNA_CLEAVAGE_FACTOR_COMPLEX	0.02777 8

	GOCC_NUCLEAR_EXOSOME_RNASE_COMPLEX	0.027778
	GOCC_RNA_POLYMERASE_III_COMPLEX	0.027778
	GOMF_5_3_EXONUCLEASE_ACTIVITY	0.027778
	GOMF_CYSTEINE_TYPE_ENDOPEPTIDASE_ACTIVITY_INVOLVED_IN_APOPTOTIC_PROCESS	0.027778
	GOMF_FOUR_WAY_JUNCTION_DNA_BINDING	0.027778
	GOMF_MISMATCHED_DNA_BINDING	0.027778
	GOMF_PRENYLTRANSFERASE_ACTIVITY	0.027778
	GOMF_PSEUDOURIDINE_SYNTHASE_ACTIVITY	0.027778

	GOMF_U6_SNRNA_BINDING	0.027778
	GOBP_MATURE_B_CELL_DIFFERENTIATION	0.028169
	GOBP_POSITIVE_REGULATION_OF_RELEASE_OF_SEQUESTERED_CALCIUM_ION_INTO_CYTOSOL	0.028169
	GOBP_POSITIVE_REGULATION_OF_VASOCONSTRICTION	0.028169
	GOBP_PROTEIN_CONTAINING_COMPLEX_REMODELING	0.028169
	GOBP_PROXIMAL_DISTAL_PATTERN_FORMATION	0.028169
	GOBP_REGULATION_OF_COLLAGEN_METABOLIC_PROCESS	0.028169
	GOBP_REGULATION_OF_HOMOTYPIC_CELL_CELL_ADHESION	0.028169

	GOBP_VENTRICULAR_CARDIAC_MUSCLE_CELL_ACTION_POTENTIAL	0.028169
	GOCC_INTEGRAL_COMPONENT_OF_SYNAPTIC_VESICLE_MEMBRANE	0.028169
	GOBP_AMINO_ACID_ACTIVATION	0.028571
	GOBP_ANTIGEN_PROCESSING_AND_PRESENTATION_OF_PEPTIDE_ANTIGEN_VIA_MHC_CLASS_I	0.028571
	GOBP_BONE_CELL_DEVELOPMENT	0.028571
	GOBP_CELL_DIFFERENTIATION_INVOLVED_IN_EMBRYONIC_PLACENTA_DEVELOPMENT	0.028571
	GOBP_DENDRITE_EXTENSION	0.028571
	GOBP_DNA_REPLICATION_INITIATION	0.028571

	GOBP_EXTRACELLULAR_VESICLE_BIOGENESIS	0.02 857 1
	GOBP_FEMALE_MEIOTIC_NUCLEAR_DIVISION	0.02 857 1
	GOBP_INTERFERON_GAMMA_MEDIATED_SIGNALING_PATHWAY	0.02 857 1
	GOBP_LONG_TERM_MEMORY	0.02 857 1
	GOBP_MATURATION_OF_5_8S_RRNA	0.02 857 1
	GOBP_MICROTUBULE_ORGANIZING_CENTER_LOCALIZATION	0.02 857 1
	GOBP_MITOTIC_G1_S_TRANSITION_CHECKPOINT_SIGNALING	0.02 857 1
	GOBP_MITOTIC_G2_DNA_DAMAGE_CHECKPOINT_SIGNALING	0.02 857 1

	GOBP_MITOTIC_RECOMBINATION	0.02<br>857<br>1
	GOBP_NAD_METABOLIC_PROCESS	0.02<br>857<br>1
	GOBP_NEGATIVE_REGULATION_OF_DNA_REPAIR	0.02<br>857<br>1
	GOBP_POLYOL_CATABOLIC_PROCESS	0.02<br>857<br>1
	GOBP_POSITIVE_REGULATION_OF_NATURAL_KILLER_CELL_MEDIATED_CYTOTOXICITY	0.02<br>857<br>1
	GOBP_POSITIVE_REGULATION_OF_VASCULAR_ASSOCIATED_SMOOTH_MUSCLE_CELL_PROLIFERATION	0.02<br>857<br>1
	GOBP_PROTEIN_LOCALIZATION_TO_CHROMOSOME_TELOMERIC_REGION	0.02<br>857<br>1
	GOBP_REGULATION_OF_INTRINSIC_APOPTOTIC_SIGNALING_PATHWAY_BY_P53_CLASS_MEDIATOR	0.02<br>857<br>1

	GOBP_RIG_I_SIGNALING_PATHWAY	0.028571
	GOBP_RRNA_TRANSCRIPTION	0.028571
	GOBP_SKELETAL_MUSCLE_TISSUE_REGENERATION	0.028571
	GOCC_DESMOSOME	0.028571
	GOCC_ENDORIBONUCLEASE_COMPLEX	0.028571
	GOCC_NUCLEAR_REPLICATION_FORK	0.028571
	GOCC_SEX_CHROMOSOME	0.028571
	GOCC_SMALL_SUBUNIT_PROCESSOME	0.028571

	GOMF_DELAYED_RECTIFIER_POTASSIUM_CHANNEL_ACTIVITY	0.02857 1
	GOMF_ENDORIBONUCLEASE_ACTIVITY_PRODUCING_5_PHOSPHOMONOESTERS	0.02857 1
	GOMF_MHC_PROTEIN_BINDING	0.02857 1
	GOMF_NUCLEOSOMAL_DNA_BINDING	0.02857 1
	GOMF_OXIDOREDUCTASE_ACTIVITY_ACTING_ON_THE_CH_NH_GROUP_OF_DONORS	0.02857 1
	GOMF_PEPTIDE_ANTIGEN_BINDING	0.02857 1
	GOBP_DETECTION_OF_MECHANICAL_STIMULUS_INVOLVED_IN_SENSORY_PERCEPTION	0.02898 6
	GOBP_NEGATIVE_REGULATION_OF_LYASE_ACTIVITY	0.02898 6

	GOBP_REGULATION_OF_CAMP_MEDIATED_SIGNALING	0.028986
	GOBP_SYNAPTIC_VESICLE_MEMBRANE_ORGANIZATION	0.028986
	GOBP_THYROID_GLAND_DEVELOPMENT	0.028986
	GOBP_VASCULAR_ASSOCIATED_SMOOTH_MUSCLE_CELL_DIFFERENTIATION	0.028986
	GOCC_PODOSOME	0.028986
	GOMF_CHEMOATTRACTANT_ACTIVITY	0.028986
	GOBP_MATURATION_OF_SSU_RRNA_FROM_TRICISTRONIC_RRNA_TRANSCRIPT_SSU_RRNA_5_8S_RRNA_LSU_RRNA	0.029412
	GOBP_NEGATIVE_REGULATION_OF_DNA_REPLICATION	0.029412

	GOBP_PROTEIN_TRANSMEMBRANE_IMPORT_INTO_INTRACELLULAR_ORGANELLE	0.029412
	GOMF_CARBOXY_LYASE_ACTIVITY	0.029412
	GOMF_DNA_POLYMERASE_ACTIVITY	0.029412
	GOBP_AMYLOID_FIBRIL_FORMATION	0.029851
	GOBP_EXCITATORY_SYNAPSE_ASSEMBLY	0.029851
	GOBP_EXPLORATION_BEHAVIOR	0.029851
	GOBP_NEGATIVE_REGULATION_OF_EXTRINSIC_APOPTOTIC_SIGNALING_PATHWAY_VIA_DEATH_DOMAIN_RECEPTORS	0.029851
	GOBP_NEGATIVE_REGULATION_OF_MUSCLE_HYPERTROPHY	0.029851

	GOBP_POSITIVE_REGULATION_OF_GRANULOCYTE_CHEMOTAXIS	0.02<br>985<br>1
	GOBP_POSITIVE_REGULATION_OF_NEUROBLAST_PROLIFERATION	0.02<br>985<br>1
	GOBP_REGULATION_OF_CELL_FATE_COMMITMENT	0.02<br>985<br>1
	GOBP_REPLACEMENT_OSSIFICATION	0.02<br>985<br>1
	GOBP_RESPONSE_TO_INCREASED_OXYGEN_LEVELS	0.02<br>985<br>1
	GOBP_RESPONSE_TO_THYROID_HORMONE	0.02<br>985<br>1
	GOBP_SPINAL_CORD_MOTOR_NEURON_DIFFERENTIATION	0.02<br>985<br>1
	GOCC_FILAMENTOUS_ACTIN	0.02<br>985<br>1
	GOBP_POSITIVE_REGULATION_OF_LOCOMOTION	0.03

	GOBP_TRANSMEMBRANE_RECEPTOR_PROTEIN_SERINE_THREONINE_KINASE_SIGNALING_PATHWAY	0.03
	GOCC_CELL_BODY	0.03
	GOCC_ENDOPLASMIC_RETICULUM_LUMEN	0.03
	GOBP_ADHERENS_JUNCTION_ASSEMBLY	0.030303
	GOBP_ANGIOTENSIN_ACTIVATED_SIGNALING_PATHWAY	0.030303
	GOBP_ANTIFUNGAL_INNATE_IMMUNE_RESPONSE	0.030303
	GOBP_AV_NODE_CELL_TO_BUNDLE_OF_HIS_CELL_COMMUNICATION	0.030303
	GOBP_BRANCHED_CHAIN_AMINO_ACID_METABOLIC_PROCESS	0.030303
	GOBP_CELLULAR_COMPONENT_DISASSEMBLY_INVOLVED_IN_EXECUTION_PHASE_OF_APOPTOSIS	0.030303
	GOBP_COMPLEMENT_DEPENDENT_CYTOTOXICITY	0.030303

	GOBP_DENTATE_GYRUS_DEVELOPMENT	0.03 030 3
	GOBP_DEOXYRIBOSE_PHOSPHATE_CATABOLIC_PROCESS	0.03 030 3
	GOBP_EPITHELIAL_CELL_CELL_ADHESION	0.03 030 3
	GOBP_ESTROUS_CYCLE	0.03 030 3
	GOBP_GONADOTROPIN_SECRETION	0.03 030 3
	GOBP_INTERFERON_ALPHA_PRODUCTION	0.03 030 3
	GOBP_MECHANOSENSORY_BEHAVIOR	0.03 030 3
	GOBP_MESENCHYMAL_TO_EPITHELIAL_TRANSITION_INVOLVED_IN_METANEPHROS_MORPHOGENESIS	0.03 030 3

	GOBP_METANEPHRIC_MESENCHYME_DEVELOPMENT	0.03030 3
	GOBP_MICROVILLUS_ORGANIZATION	0.03030 3
	GOBP_MISMATCH_REPAIR	0.03030 3
	GOBP_MYOBLAST_PROLIFERATION	0.03030 3
	GOBP_NEGATIVE_REGULATION_OF_CARDIAC_MUSCLE_CELL_PROLIFERATION	0.03030 3
	GOBP_NEGATIVE_REGULATION_OF_GLUCONEOGENESIS	0.03030 3
	GOBP_POSITIVE_REGULATION_OF_RENAL_SODIUM_EXCRETION	0.03030 3
	GOBP_POTASSIUM_ION_EXPORT_ACROSS_PLASMA_MEMBRANE	0.03030 3

	GOBP_REGULATION_OF_ACUTE_INFLAMMATORY_RESPONSE_TO_ANTIGENIC_STIMULUS	0.030303
	GOBP_REGULATION_OF_EXTRACELLULAR_MATRIX_DISASSEMBLY	0.030303
	GOBP_REGULATION_OF_RELAXATION_OF_MUSCLE	0.030303
	GOBP_REGULATION_OF_SPINDLE_ASSEMBLY	0.030303
	GOBP_REGULATION_OF_TELOMERE_CAPPING	0.030303
	GOBP_REGULATION_OF_VASCULOGENESIS	0.030303
	GOBP_STEROID_ESTERIFICATION	0.030303
	GOCC_MITOCHONDRIAL_SMALL_RIBOSOMAL_SUBUNIT	0.030303

	GOMF_INOSITOL_1_4_5_TRISPHOSPHATE_BINDING	0.030303
	GOMF_OXIDOREDUCTASE_ACTIVITY_ACTING_ON_THE_CH_CH_GROUP_OF_DONORS_NAD_OR_NADP_AS_ACCEPTOR	0.030303
	GOMF_RETINOL_BINDING	0.030303
	GOMF_STRUCTURAL_CONSTITUENT_OF_SYNAPSE	0.030303
	GOBP_TISSUE_REMODELING	0.030612
	GOBP_CELL_ADHESION_MOLECULE_PRODUCTION	0.030769
	GOBP_CELLULAR_RESPONSE_TO_POTASSIUM_ION	0.030769
	GOBP_CHOLESTEROL_CATABOLIC_PROCESS	0.030769

	GOBP_DETECTION_OF_TEMPERATURE_STIMULUS_INVOLVED_IN_SENSORY_PERCEPTION_OF_PAIN	0.030769
	GOBP_LEUKOCYTE_AGGREGATION	0.030769
	GOBP_MAST_CELL_MIGRATION	0.030769
	GOBP_NEGATIVE_REGULATION_OF_ACTIVIN_RECEPTOR_SIGNALING_PATHWAY	0.030769
	GOBP_NEGATIVE_REGULATION_OF_NEUROTRANSMITTER_TRANSPORT	0.030769
	GOBP_NOREPINEPHRINE_SECRETION	0.030769
	GOBP_POSITIVE_REGULATION_OF_ENDOTHELIAL_CELL_DIFFERENTIATION	0.030769
	GOBP_POSITIVE_REGULATION_OF_GLUTAMATE_SECRETION	0.030769

	GOBP_POSITIVE_REGULATION_OF_OSTEOBLAST_PROLIFERATION	0.03 076 9
	GOBP_POSITIVE_REGULATION_OF_URINE_VOLUME	0.03 076 9
	GOBP_POSITIVE_REGULATION_OF_VASCULAR_ENDOTHELIAL_GROWTH_FACTOR_RECEPTOR_SIGNALING_PATHWAY	0.03 076 9
	GOBP_POSITIVE_REGULATION_OF_VASCULOGENESIS	0.03 076 9
	GOBP_POSTSYNAPTIC_MEMBRANE_ASSEMBLY	0.03 076 9
	GOBP_REGULATION_OF_CHOLESTEROL_ESTERIFICATION	0.03 076 9
	GOBP_REGULATION_OF_LONG_TERM_SYNAPTIC_DEPRESSION	0.03 076 9
	GOBP_REGULATION_OF_POSTSYNAPTIC_DENSITY_ORGANIZATION	0.03 076 9

	GOBP_REGULATION_OF_POSTSYNAPTIC_SPECIALIZATION_ASSEMBLY	0.030769
	GOBP_SECONDARY_HEART_FIELD_SPECIFICATION	0.030769
	GOBP_SECRETORY_GRANULE_LOCALIZATION	0.030769
	GOBP_SYNAPSE_PRUNING	0.030769
	GOBP_TRANS_SYNAPTIC_SIGNALING_MODULATING_SYNAPTIC_TRANSMISSION	0.030769
	GOCC_SPERM_HEAD	0.030769
	GOMF_MYOSIN_HEAVY_CHAIN_BINDING	0.030769
	GOMF_PLATELET_DERIVED_GROWTH_FACTOR_RECEPTOR_BINDING	0.030769

	GOBP_CYTOSOLIC_CALCIUM_ION_TRANSPORT	0.030928
	GOBP_DENDRITE_MORPHOGENESIS	0.030928
	GOBP_MULTICELLULAR_ORGANISMAL_SIGNALING	0.030928
	GOBP_NEGATIVE_REGULATION_OF_WNT_SIGNALING_PATHWAY	0.030928
	GOBP_ORGAN_GROWTH	0.030928
	GOBP_POSITIVE_REGULATION_OF_DEVELOPMENTAL_GROWTH	0.030928
	GOBP_REGULATION_OF_MUSCLE_CONTRACTION	0.030928
	GOBP_REGULATION_OF_REPRODUCTIVE_PROCESS	0.030928

	GOBP_RESPONSE_TO_CORTICOSTEROID	0.03092 8
	GOCC_ADHERENS_JUNCTION	0.03092 8
	GOCC_ANCHORED_COMPONENT_OF_MEMBRANE	0.03092 8
	GOCC_INTRINSIC_COMPONENT_OF_SYNAPTIC_MEMBRANE	0.03092 8
	GOCC_NEURON_SPINE	0.03092 8
	GOCC_SARCOLEMMA	0.03092 8
	GOCC_SITE_OF_POLARIZED_GROWTH	0.03092 8
	GOCC_VACUOLAR_LUMEN	0.03092 8

	GOMF_CHANNEL_REGULATOR_ACTIVITY	0.030928
	GOBP_ACTIN_FILAMENT_BASED_MOVEMENT	0.03125
	GOBP_AMINE_METABOLIC_PROCESS	0.03125
	GOBP_BIOMINERALIZATION	0.03125
	GOBP_BLOOD_VESSEL_ENDOTHELIAL_CELL_MIGRATION	0.03125
	GOBP_BRANCHING_MORPHOGENESIS_OF_AN_EPITHELIAL_TUBE	0.03125
	GOBP_CARDIAC_CHAMBER_MORPHOGENESIS	0.03125
	GOBP_CARDIAC_MUSCLE_CONTRACTION	0.03125
	GOBP_CARDIOCYTE_DIFFERENTIATION	0.03125
	GOBP_CHROMOSOME_CONDENSATION	0.03125
	GOBP_DIGESTIVE_SYSTEM_DEVELOPMENT	0.03125
	GOBP_EMBRYONIC_APPENDAGE_MORPHOGENESIS	0.03125

	GOBP_EMBRYONIC_SKELETAL_SYSTEM_DEVELOPMENT	0.03125
	GOBP_ENDOTHELIUM_DEVELOPMENT	0.03125
	GOBP_ENSHEATHMENT_OF_NEURONS	0.03125
	GOBP_EPITHELIAL_TO_MESENCHYMAL_TRANSITION	0.03125
	GOBP_EYE_MORPHOGENESIS	0.03125
	GOBP_FEMALE_SEX_DIFFERENTIATION	0.03125
	GOBP_FORMATION_OF_PRIMARY_GERM_LAYER	0.03125
	GOBP_MEMORY	0.03125
	GOBP_NEGATIVE_REGULATION_OF_CANONICAL_WNT_SIGNALING_PATHWAY	0.03125
	GOBP_NEGATIVE_REGULATION_OF_ION_TRANSPORT	0.03125
	GOBP_NEGATIVE_REGULATION_OF_NEURON_PROJECTION_DEVELOPMENT	0.03125
	GOBP_NEGATIVE_REGULATION_OF_PROTEIN_CONTAINING_COMPLEX_ASSEMBLY	0.03125
	GOBP_NEGATIVE_REGULATION_OF_TRANSMEMBRANE_RECEPTOR_PROTEIN_SERINE_THREONINE_KINASE_SIGNALING_PATHWAY	0.03125

	GOBP_NEPHRON_EPITHELIUM_DEVELOPMENT	0.03125
	GOBP_ODONTOGENESIS	0.03125
	GOBP_PLASMA_MEMBRANE_ORGANIZATION	0.03125
	GOBP_POSITIVE_REGULATION_OF_WNT_SIGNALING_PATHWAY	0.03125
	GOBP_RECEPTOR_METABOLIC_PROCESS	0.03125
	GOBP_REGULATION_OF_AXONOGENESIS	0.03125
	GOBP_REGULATION_OF_CALCIUM_ION_TRANSMEMBRANE_TRANSPORT	0.03125
	GOBP_REGULATION_OF_CELL_COMMUNICATION_BY_ELECTRICAL_COUPLING	0.03125
	GOBP_REGULATION_OF_CELL_MATRIX_ADHESION	0.03125
	GOBP_REGULATION_OF_MUSCLE_CELL_DIFFERENTIATION	0.03125
	GOBP_REGULATION_OF_NERVOUS_SYSTEM_PROCESS	0.03125
	GOBP_REGULATION_OF_OSTEOBLAST_DIFFERENTIATION	0.03125
	GOBP_REGULATION_OF_POSTSYNAPTIC_MEMBRANE_POTENTIAL	0.03125

	GOBP_REGULATION_OF_POSTSYNAPTIC_NEUROTRANSMITTER_RECEPTOR_ACTIVITY	0.03125
	GOBP_RESPONSE_TO_NUTRIENT	0.03125
	GOBP_SKELETAL_MUSCLE_ORGAN_DEVELOPMENT	0.03125
	GOBP_SPECIFICATION_OF_SYMMETRY	0.03125
	GOBP_TUBE_FORMATION	0.03125
	GOCC_INTRINSIC_COMPONENT_OF_POSTSYNAPTIC_MEMBRANE	0.03125
	GOCC_PROTEIN_PHOSPHATASE_TYPE_1_COMPLEX	0.03125
	GOCC_SPINDLE_MIDZONE	0.03125
	GOCC_TRANSCRIPTION_FACTOR_TFIID_COMPLEX	0.03125
	GOMF_CALCIUM_ION_TRANSMEMBRANE_TRANSPORTER_ACTIVITY	0.03125
	GOMF_ICOSANOID_RECEPTOR_ACTIVITY	0.03125
	GOMF_INTEGRIN_BINDING	0.03125

	GOBP_ACTIN_FILAMENT_BUNDLE_ORGANIZATION	0.03<br>157<br>9
	GOBP_ACTIN_FILAMENT_POLYMERIZATION	0.03<br>157<br>9
	GOBP_ADENYLATE_CYCLASE_ACTIVATING_G_PROTEIN_COUPLED<br>_RECEPTOR_SIGNALING_PATHWAY	0.03<br>157<br>9
	GOBP_AMINOGLYCAN_METABOLIC_PROCESS	0.03<br>157<br>9
	GOBP_AXON_EXTENSION	0.03<br>157<br>9
	GOBP_CALCIUM_ION_TRANSPORT_INTO_CYTOSOL	0.03<br>157<br>9
	GOBP_CAMERA_TYPE_EYE_MORPHOGENESIS	0.03<br>157<br>9
	GOBP_CARDIAC_VENTRICLE_DEVELOPMENT	0.03<br>157<br>9

	GOBP_CELL_RECOGNITION	0.031579
	GOBP_DETECTION_OF_ABIOTIC_STIMULUS	0.031579
	GOBP_ENDOTHELIAL_CELL_PROLIFERATION	0.031579
	GOBP_GLAND_MORPHOGENESIS	0.031579
	GOBP_HINDBRAIN_DEVELOPMENT	0.031579
	GOBP_KIDNEY_EPITHELIUM_DEVELOPMENT	0.031579
	GOBP_MORPHOGENESIS_OF_EMBRYONIC_EPITHELIUM	0.031579
	GOBP_NEGATIVE_REGULATION_OF_EPITHELIAL_CELL_PROLIFERATION	0.031579

	GOBP_NEPHRON_DEVELOPMENT	0.03<br>157<br>9
	GOBP_NEURAL_TUBE_DEVELOPMENT	0.03<br>157<br>9
	GOBP_NEURON_MIGRATION	0.03<br>157<br>9
	GOBP_PLATELET_ACTIVATION	0.03<br>157<br>9
	GOBP_POSITIVE_REGULATION_OF_ION_TRANSMEMBRANE_TRANS<br>PORT	0.03<br>157<br>9
	GOBP_POSITIVE_REGULATION_OF_SYNAPTIC_TRANSMISSION	0.03<br>157<br>9
	GOBP_POSITIVE_REGULATION_OF_TRANSMEMBRANE_RECEPTOR_<br>PROTEIN_SERINE_THREONINE_KINASE_SIGNALING_PATHWAY	0.03<br>157<br>9
	GOBP_POSITIVE_REGULATION_OF_VASCULATURE_DEVELOPMENT	0.03<br>157<br>9

	GOBP_POSTSYNAPSE_ORGANIZATION	0.03<br>157<br>9
	GOBP_REGULATION_OF_CATION_CHANNEL_ACTIVITY	0.03<br>157<br>9
	GOBP_REGULATION_OF_CELLULAR_RESPONSE_TO_TRANSFORMING_GROWTH_FACTOR_BETA_STIMULUS	0.03<br>157<br>9
	GOBP_REGULATION_OF_G_PROTEIN_COUPLED_RECEPTOR_SIGNALING_PATHWAY	0.03<br>157<br>9
	GOBP_REGULATION_OF_LEUKOCYTE_CHEMOTAXIS	0.03<br>157<br>9
	GOBP_REGULATION_OF_RESPONSE_TO_WOUNDING	0.03<br>157<br>9
	GOBP_REGULATION_OF_WOUND_HEALING	0.03<br>157<br>9
	GOBP_RETINA_DEVELOPMENT_IN_CAMERA_TYPE_EYE	0.03<br>157<br>9

	GOBP_RHO_PROTEIN_SIGNAL_TRANSDUCTION	0.031579
	GOBP_SEQUESTERING_OF_CALCIUM_ION	0.031579
	GOBP_SMOOTHENED_SIGNALING_PATHWAY	0.031579
	GOBP_SPROUTING_ANGIOGENESIS	0.031579
	GOCC_NEURON_PROJECTION_TERMINUS	0.031579
	GOCC_PERIKARYON	0.031579
	GOCC_PRESYNAPTIC_MEMBRANE	0.031579
	GOMF_GROWTH_FACTOR_ACTIVITY	0.031579

	GOMF_HEPARIN_BINDING	0.03 157 9
	GOMF_POTASSIUM_CHANNEL_ACTIVITY	0.03 157 9
	GOMF_POTASSIUM_ION_TRANSMEMBRANE_TRANSPORTER_ACTIVITY	0.03 157 9
	GOMF_PROTEASE_BINDING	0.03 157 9
	GOMF_TRANSMEMBRANE_TRANSPORTER_BINDING	0.03 157 9
	GOBP_MEMBRANE_RAFT_ORGANIZATION	0.03 174 6
	GOBP_MESENCHYMAL_TO_EPITHELIAL_TRANSITION	0.03 174 6
	GOBP_NEUROEPITHELIAL_CELL_DIFFERENTIATION	0.03 174 6

	GOBP_PHASIC_SMOOTH_MUSCLE_CONTRACTION	0.031746
	GOBP_VESICLE_FUSION_TO_PLASMA_MEMBRANE	0.031746
	GOMF_IMMUNOGLOBULIN_BINDING	0.031746
	GOMF_LOW_DENSITY_LIPOPROTEIN_PARTICLE_RECEPTOR_BINDING	0.031746
	GOBP_ACTION_POTENTIAL	0.031915
	GOBP_ADULT_BEHAVIOR	0.031915
	GOBP_APPENDAGE_MORPHOGENESIS	0.031915
	GOBP_BONE_MINERALIZATION	0.031915

	GOBP_CELL_FATE_SPECIFICATION	0.03<br>191<br>5
	GOBP_ENDOCRINE_SYSTEM_DEVELOPMENT	0.03<br>191<br>5
	GOBP_EPITHELIAL_TUBE_FORMATION	0.03<br>191<br>5
	GOBP_HOMOPHILIC_CELL_ADHESION_VIA_PLASMA_MEMBRANE_<br>ADHESION_MOLECULES	0.03<br>191<br>5
	GOBP_LEARNING	0.03<br>191<br>5
	GOBP_NEGATIVE_REGULATION_OF_CYTOSKELETON_ORGANIZATI<br>ON	0.03<br>191<br>5
	GOBP_NEGATIVE_REGULATION_OF_DEVELOPMENTAL_GROWTH	0.03<br>191<br>5
	GOBP_NEGATIVE_REGULATION_OF_SUPRAMOLECULAR_FIBER_OR<br>GANIZATION	0.03<br>191<br>5

	GOBP_NEURAL_PRECURSOR_CELL_PROLIFERATION	0.03<br>191<br>5
	GOBP_NEUROMUSCULAR_PROCESS	0.03<br>191<br>5
	GOBP_NEUROTRANSMITTER_SECRETION	0.03<br>191<br>5
	GOBP_PHENOL_CONTAINING_COMPOUND_METABOLIC_PROCESS	0.03<br>191<br>5
	GOBP_PHOSPHATIDYLINOSITOL_3_KINASE_SIGNALING	0.03<br>191<br>5
	GOBP_POSITIVE_REGULATION_OF_CALCIUM_ION_TRANSPORT	0.03<br>191<br>5
	GOBP_POSITIVE_REGULATION_OF_CHEMOTAXIS	0.03<br>191<br>5
	GOBP_POSITIVE_REGULATION_OF_EPITHELIAL_CELL_MIGRATION	0.03<br>191<br>5

	GOBP_POSITIVE_REGULATION_OF_LIPID_METABOLIC_PROCESS	0.031915
	GOBP_POSITIVE_REGULATION_OF_NEURON_PROJECTION_DEVELOPMENT	0.031915
	GOBP_POSITIVE_REGULATION_OF_PEPTIDYL_SERINE_PHOSPHORYLATION	0.031915
	GOBP_REGULATION_OF_ANIMAL_ORGAN_MORPHOGENESIS	0.031915
	GOBP_REGULATION_OF_CELL_SHAPE	0.031915
	GOBP_REGULATION_OF_EXTENT_OF_CELL_GROWTH	0.031915
	GOBP_REGULATION_OF_RECEPTOR_MEDIATED_ENDOCYTOSIS	0.031915
	GOBP_REGULATION_OF_TUBE_SIZE	0.031915

	GOBP_RESPONSE_TO_CALCIUM_ION	0.031915
	GOBP_RESPONSE_TO_FIBROBLAST_GROWTH_FACTOR	0.031915
	GOBP_RESPONSE_TO_RETINOIC_ACID	0.031915
	GOBP_SMOOTH_MUSCLE_CELL_PROLIFERATION	0.031915
	GOCC_I_BAND	0.031915
	GOCC_PLASMA_MEMBRANE_RAFT	0.031915
	GOMF_CYTOKINE_BINDING	0.031915
	GOMF_GROWTH_FACTOR_BINDING	0.031915

	GOMF_HORMONE_ACTIVITY	0.03<br>191<br>5
	GOMF_PEPTIDE_RECEPTOR_ACTIVITY	0.03<br>191<br>5
	GOMF_VOLTAGE_GATED_CATION_CHANNEL_ACTIVITY	0.03<br>191<br>5
	GOBP_ATTACHMENT_OF_SPINDLE_MICROTUBULES_TO_KINETOCH<br>ORE	0.03<br>225<br>8
	GOBP_CARDIAC_MUSCLE_CELL_DIFFERENTIATION	0.03<br>225<br>8
	GOBP_CELLULAR_COMPONENT_ASSEMBLY_INVOLVED_IN_MORPH<br>OGENESIS	0.03<br>225<br>8
	GOBP_CHEMICAL_SYNAPTIC_TRANSMISSION_POSTSYNAPTIC	0.03<br>225<br>8
	GOBP_CYCLIC_NUCLEOTIDE_BIOSYNTHETIC_PROCESS	0.03<br>225<br>8

	GOBP_DETECTION_OF_TEMPERATURE_STIMULUS	0.032258
	GOBP_DEVELOPMENT_OF_PRIMARY_FEMALE_SEXUAL_CHARACTERISTICS	0.032258
	GOBP_DNA_STRAND_ELONGATION	0.032258
	GOBP_DNA_SYNTHESIS_INVOLVED_IN_DNA_REPAIR	0.032258
	GOBP_ESTABLISHMENT_OF_MITOTIC_SPINDLE_LOCALIZATION	0.032258
	GOBP_GLYCOSYL_COMPOUND_CATABOLIC_PROCESS	0.032258
	GOBP_INNER_MITOCHONDRIAL_MEMBRANE_ORGANIZATION	0.032258
	GOBP_INTEGRIN_MEDIATED_SIGNALING_PATHWAY	0.032258

	GOBP_KERATINIZATION	0.032258
	GOBP_NEGATIVE_REGULATION_OF_BLOOD_CIRCULATION	0.032258
	GOBP_NEGATIVE_REGULATION_OF_CELLULAR_RESPONSE_TO_GROWTH_FACTOR_STIMULUS	0.032258
	GOBP_NEUROPEPTIDE_SIGNALING_PATHWAY	0.032258
	GOBP_NEUTRAL_LIPID_BIOSYNTHETIC_PROCESS	0.032258
	GOBP_PHOSPHOLIPASE_C_ACTIVATING_G_PROTEIN_COUPLED_RECEPTOR_SIGNALING_PATHWAY	0.032258
	GOBP_POSITIVE_REGULATION_OF_CELL_JUNCTION_ASSEMBLY	0.032258
	GOBP_POSITIVE_REGULATION_OF_CELL_SUBSTRATE_ADHESION	0.032258

	GOBP_POSITIVE_REGULATION_OF_MONOOXYGENASE_ACTIVITY	0.032258
	GOBP_POSITIVE_REGULATION_OF_PROTEIN_KINASE_B_SIGNALING	0.032258
	GOBP_POSTREPLICATION_REPAIR	0.032258
	GOBP_RECEPTOR_INTERNALIZATION	0.032258
	GOBP_REGULATION_OF_CELL_CYCLE_CHECKPOINT	0.032258
	GOBP_REGULATION_OF_FAT_CELL_DIFFERENTIATION	0.032258
	GOBP_REGULATION_OF_OSSIFICATION	0.032258
	GOBP_REGULATION_OF_PROTEIN_EXPORT_FROM_NUCLEUS	0.032258

	GOBP_REGULATION_OF_TRANSCRIPTION_BY_RNA_POLYMERASE_I	0.032258
	GOBP_REGULATION_OF_TRANSCRIPTION_ELONGATION_FROM_RNA_POLYMERASE_II_PROMOTER	0.032258
	GOBP_REGULATORY_T_CELL_DIFFERENTIATION	0.032258
	GOBP_REPLICATION_FORK_PROCESSING	0.032258
	GOBP_RESPONSE_TO_ACID_CHEMICAL	0.032258
	GOBP_RIBONUCLEOSIDE_MONOPHOSPHATE_BIOSYNTHETIC_PROCESS	0.032258
	GOBP_SIALYLATION	0.032258
	GOBP_SPLICEOSOMAL_SNRNP_ASSEMBLY	0.032258

	GOBP_TETRAPYRROLE_BIOSYNTHETIC_PROCESS	0.032258
	GOBP_TRICARBOXYLIC_ACID_CYCLE	0.032258
	GOBP_TRNA_METHYLATION	0.032258
	GOBP_ZINC_ION_TRANSPORT	0.032258
	GOCC_ACTIN_FILAMENT	0.032258
	GOCC_ANCHORED_COMPONENT_OF_EXTERNAL_SIDE_OF_PLASMA_MEMBRANE	0.032258
	GOCC_AXON_INITIAL_SEGMENT	0.032258
	GOCC_DNA_REPAIR_COMPLEX	0.032258

	GOCC_MEDIATOR_COMPLEX	0.03<br>225<br>8
	GOCC_POSTSYNAPTIC_SPECIALIZATION_MEMBRANE	0.03<br>225<br>8
	GOCC_SPLICEOSOMAL_TRI_SNRNP_COMPLEX	0.03<br>225<br>8
	GOCC_U2_TYPE_CATALYTIC_STEP_2_SPLICEOSOME	0.03<br>225<br>8
	GOMF_DNA_SECONDARY_STRUCTURE_BINDING	0.03<br>225<br>8
	GOMF_ENDONUCLEASE_ACTIVITY_ACTIVE_WITH_EITHER_RIBO_OR_DEOXYRIBONUCLEIC_ACIDS_AND_PRODUCING_5_PHOSPHOMONOESTERS	0.03<br>225<br>8
	GOMF_EXORIBONUCLEASE_ACTIVITY_PRODUCING_5_PHOSPHOMONOESTERS	0.03<br>225<br>8
	GOMF_NOTCH_BINDING	0.03<br>225<br>8

	GOMF_NUCLEOTIDE_RECEPTOR_ACTIVITY	0.032258
	GOMF_PEPTIDE_HORMONE_RECEPTOR_BINDING	0.032258
	GOMF_POLY_A_BINDING	0.032258
	GOMF_RNA_POLYMERASE_ACTIVITY	0.032258
	GOMF_THIOLESTER_HYDROLASE_ACTIVITY	0.032258
	GOMF_TUMOR_NECROSIS_FACTOR_RECEPTOR_BINDING	0.032258
	GOBP_ACTIN_MEDIATED_CELL_CONTRACTION	0.032609
	GOBP_AMINE_TRANSPORT	0.032609

	GOBP_ARTERY_DEVELOPMENT	0.03<br>260<br>9
	GOBP_CARDIAC_SEPTUM_DEVELOPMENT	0.03<br>260<br>9
	GOBP_CHONDROCYTE_DIFFERENTIATION	0.03<br>260<br>9
	GOBP_GLIAL_CELL_DEVELOPMENT	0.03<br>260<br>9
	GOBP_MESONEPHROS_DEVELOPMENT	0.03<br>260<br>9
	GOBP_MUSCLE_ADAPTATION	0.03<br>260<br>9
	GOBP_MYOTUBE_DIFFERENTIATION	0.03<br>260<br>9
	GOBP_NEGATIVE_REGULATION_OF_TRANSMEMBRANE_TRANSPOR<br>T	0.03<br>260<br>9

	GOBP_NEURAL_CREST_CELL_DIFFERENTIATION	0.032609
	GOBP_POSITIVE_REGULATION_OF_ENDOTHELIAL_CELL_MIGRATION	0.032609
	GOBP_POSITIVE_REGULATION_OF_TRANSPORTER_ACTIVITY	0.032609
	GOBP_REGULATION_OF_BMP_SIGNALING_PATHWAY	0.032609
	GOBP_REGULATION_OF_HEART_RATE	0.032609
	GOBP_REGULATION_OF_PHOSPHATIDYLINOSITOL_3_KINASE_SIGNALING	0.032609
	GOBP_REGULATION_OF_POTASSIUM_ION_TRANSPORT	0.032609
	GOBP_RENAL_SYSTEM_PROCESS	0.032609

	GOBP_SKIN_EPIDERMIS_DEVELOPMENT	0.03<br>260<br>9
	GOBP_SMOOTH_MUSCLE_CONTRACTION	0.03<br>260<br>9
	GOBP_SUBSTRATE_ADHESION_DEPENDENT_CELL_SPREADING	0.03<br>260<br>9
	GOBP_AXONAL_TRANSPORT_OF_MITOCHONDRION	0.03<br>278<br>7
	GOBP_COMPLEMENT_ACTIVATION_ALTERNATIVE_PATHWAY	0.03<br>278<br>7
	GOBP_CORTICOSTEROID_HORMONE_SECRETION	0.03<br>278<br>7
	GOBP_DENDRITE_SELF_AVOIDANCE	0.03<br>278<br>7
	GOBP_DETECTION_OF_TEMPERATURE_STIMULUS_INVOLVED_IN_SENSORY_PERCEPTION	0.03<br>278<br>7

	GOBP_FUSION_OF_SPERM_TO_EGG_PLASMA_MEMBRANE_INVOLVED_IN_SINGLE_FERTILIZATION	0.032787
	GOBP_GLOMERULAR_MESANGIUM_DEVELOPMENT	0.032787
	GOBP_INHIBITORY_SYNAPSE_ASSEMBLY	0.032787
	GOBP_MACROPHAGE_CYTOKINE_PRODUCTION	0.032787
	GOBP_METANEPHRIC_GLOMERULUS_DEVELOPMENT	0.032787
	GOBP_REVERSE_CHOLESTEROL_TRANSPORT	0.032787
	GOBP_TUBULIN_DEACETYLATION	0.032787
	GOBP_VASCULAR_WOUND_HEALING	0.032787

	GOMF_ADRENERGIC_RECEPTOR_BINDING	0.032787
	GOMF_CALCIUM_RELEASE_CHANNEL_ACTIVITY	0.032787
	GOMF_CYCLASE_ACTIVITY	0.032787
	GOMF_PHOSPHATIDYLINOSITOL_5_PHOSPHATE_BINDING	0.032787
	GOBP_BONE_MORPHOGENESIS	0.032967
	GOBP_STRESS_FIBER_ASSEMBLY	0.032967
	GOCC_BLOOD_MICROPARTICLE	0.032967
	GOCC_CORTICAL_CYTOSKELETON	0.032967

	GOBP_CARDIOBLAST_DIFFERENTIATION	0.03<br>333<br>3
	GOBP_HEART_FIELD_SPECIFICATION	0.03<br>333<br>3
	GOBP_HEMATOPOIETIC_STEM_CELL_HOMEOSTASIS	0.03<br>333<br>3
	GOBP_NEGATIVE_REGULATION_OF_PLATELET_ACTIVATION	0.03<br>333<br>3
	GOBP_REGULATION_OF_LIPID_KINASE_ACTIVITY	0.03<br>333<br>3
	GOBP_RESPONSE_TO_ACTIVITY	0.03<br>333<br>3
	GOBP_RESPONSE_TO_MUSCLE_ACTIVITY	0.03<br>333<br>3
	GOCC_SYNAPTIC_CLEFT	0.03<br>333<br>3

	GOMF_RECEPTOR_ANTAGONIST_ACTIVITY	0.033333
	GOBP_CELLULAR_COMPONENT_MAINTENANCE	0.037708
	GOBP_DEFENSE_RESPONSE_TO_GRAM_NEGATIVE_BACTERIUM	0.037708
	GOBP_POSITIVE_REGULATION_OF_CELL_MATRIX_ADHESION	0.037708
	GOBP_SOMITOGENESIS	0.037708
	GOBP_VESICLE_DOCKING	0.037708
	GOBP_BONE_TRABECULA_FORMATION	0.038898
	GOBP_CELL_FATE_COMMITMENT_INVOLVED_IN_PATTERN_SPECIFICATION	0.038898

	GOBP_GENITALIA_MORPHOGENESIS	0.03<br>389<br>8
	GOBP_KIDNEY_MESENCHYME_DEVELOPMENT	0.03<br>389<br>8
	GOBP_LATERAL_SPROUTING_FROM_AN_EPITHELIUM	0.03<br>389<br>8
	GOBP_MAMMARY_GLAND_EPITHELIAL_CELL_DIFFERENTIATION	0.03<br>389<br>8
	GOBP_NEGATIVE_REGULATION_OF_CALCIUM_MEDIATED_SIGNALI<br>NG	0.03<br>389<br>8
	GOBP_NEGATIVE_REGULATION_OF_CELL_ADHESION_MEDIATED_<br>BY_INTEGRIN	0.03<br>389<br>8
	GOBP_NEGATIVE_REGULATION_OF_LYMPHOCYTE_MIGRATION	0.03<br>389<br>8
	GOBP_OVULATION_FROM_OVARIAN_FOLLICLE	0.03<br>389<br>8

	GOBP_POSITIVE_REGULATION_OF_NEURON_PROJECTION_REGENERATION	0.033898
	GOBP_POSITIVE_REGULATION_OF_SPROUTING_ANGIOGENESIS	0.033898
	GOBP_REGULATION_OF_CHEMOKINE_MEDIATED_SIGNALING_PATHWAY	0.033898
	GOBP_RESPONSE_TO_HYPEROXIA	0.033898
	GOBP_SYNAPTIC_VESICLE_PRIMING	0.033898
	GOBP_TRACHEA_DEVELOPMENT	0.033898
	GOCC_FILOPODIUM_MEMBRANE	0.033898
	GOCC_JUXTAPARANODE_REGION_OF_AXON	0.033898

	GOMF_HEPARAN_SULFATE_PROTEOGLYCAN_BINDING	0.033898
	GOMF_LIPOPEPTIDE_BINDING	0.033898
	GOBP_MULTICELLULAR_ORGANISMAL_RESPONSE_TO_STRESS	0.034091
	GOMF_CELL_ADHESION_MEDIATOR_ACTIVITY	0.034091
	GOMF_GDP_BINDING	0.034091
	GOMF_MODIFIED_AMINO_ACID_BINDING	0.034091
	GOBP_CELL_CYCLE_DNA_REPLICATION	0.034483
	GOBP_CELLULAR_RESPONSE_TO_RETINOIC_ACID	0.034483

	GOBP_CYTOPLASMIC_PATTERN_RECOGNITION_RECEPTOR_SIGNALING_PATHWAY_IN_RESPONSE_TO_VIRUS	0.034483
	GOBP_HISTONE_PHOSPHORYLATION	0.034483
	GOBP_MATURATION_OF_SSU_RRNA	0.034483
	GOBP_MITOCHONDRIAL_RNA_METABOLIC_PROCESS	0.034483
	GOBP_MONOSACCHARIDE_CATABOLIC_PROCESS	0.034483
	GOBP_NADH_METABOLIC_PROCESS	0.034483
	GOBP_NCRNA_3_END_PROCESSING	0.034483
	GOBP_NEGATIVE_REGULATION_OF_TUMOR_NECROSIS_FACTOR_SUPERFAMILY_CYTOKINE_PRODUCTION	0.034483

	GOBP_NUCLEOSIDE_MONOPHOSPHATE_BIOSYNTHETIC_PROCESS	0.03 448 3
	GOBP_ONE_CARBON_METABOLIC_PROCESS	0.03 448 3
	GOBP_PEROXISOME_ORGANIZATION	0.03 448 3
	GOBP_POSITIVE_REGULATION_OF_INTERFERON_BETA_PRODUCTI ON	0.03 448 3
	GOBP_PYRIMIDINE_CONTAINING_COMPOUND_BIOSYNTHETIC_PRO CESS	0.03 448 3
	GOBP_REGULATION_OF_PROTEIN_IMPORT	0.03 448 3
	GOBP_REGULATION_OF_PROTEIN_MATURATION	0.03 448 3
	GOBP_REGULATION_OF_SPINDLE_ORGANIZATION	0.03 448 3

	GOBP_ZINC_ION_HOMEOSTASIS	0.03<br>448<br>3
	GOCC_CILIARY_MEMBRANE	0.03<br>448<br>3
	GOCC_CORTICAL_ACTIN_CYTOSKELETON	0.03<br>448<br>3
	GOCC_CYCLIN_DEPENDENT_PROTEIN_KINASE_HOLOENZYME_CO<br>MPLEX	0.03<br>448<br>3
	GOCC_FLEMMING_BODY	0.03<br>448<br>3
	GOCC_INTRINSIC_COMPONENT_OF_MITOCHONDRIAL_INNER_MEM<br>BRANE	0.03<br>448<br>3
	GOCC_NUCLEOID	0.03<br>448<br>3
	GOMF_4_IRON_4_SULFUR_CLUSTER_BINDING	0.03<br>448<br>3

	GOMF_ENDODEOXYRIBONUCLEASE_ACTIVITY	0.034483
	GOMF_EXORIBONUCLEASE_ACTIVITY	0.034483
	GOMF_N_ACETYLTRANSFERASE_ACTIVITY	0.034483
	GOMF_RNA_POLYMERASE_CORE_ENZYME_BINDING	0.034483
	GOMF_SIGNAL_SEQUENCE_BINDING	0.034483
	GOMF_TRANSITION_METAL_ION_TRANSMEMBRANE_TRANSPORTER_ACTIVITY	0.034483
	GOBP_POSITIVE_REGULATION_OF_MACROAUTOPHAGY	0.034884
	GOCC_CLATHRIN_COATED_ENDOCYTIC_VESICLE_MEMBRANE	0.034884

	GOBP_CENTRIOLE_ASSEMBLY	0.03<br>571<br>4
	GOBP_CYTOKINETIC_PROCESS	0.03<br>571<br>4
	GOBP_DEOXYRIBOSE_PHOSPHATE_METABOLIC_PROCESS	0.03<br>571<br>4
	GOBP_DNA_DEPENDENT_DNA_REPLICATION_MAINTENANCE_OF_F<br>IDELITY	0.03<br>571<br>4
	GOBP_HOMOLOGOUS_CHROMOSOME_SEGREGATION	0.03<br>571<br>4
	GOBP_INTESTINAL_ABSORPTION	0.03<br>571<br>4
	GOBP_MITOTIC_G2_M_TRANSITION_CHECKPOINT	0.03<br>571<br>4
	GOBP_MRNA_TRANSCRIPTION	0.03<br>571<br>4

	GOBP_NADH_DEHYDROGENASE_COMPLEX_ASSEMBLY	0.035714
	GOBP_NCRNA_CATABOLIC_PROCESS	0.035714
	GOBP_NEGATIVE_REGULATION_OF_PROTEASOMAL_PROTEIN_CATABOLIC_PROCESS	0.035714
	GOBP_NUCLEAR_MEMBRANE_ORGANIZATION	0.035714
	GOBP_POSITIVE_REGULATION_OF_CYTOKINESIS	0.035714
	GOBP_POSITIVE_REGULATION_OF_DNA_REPLICATION	0.035714
	GOBP_POSITIVE_REGULATION_OF_TELOMERE_MAINTENANCE_VIA_TELOMERE_LENGTHENING	0.035714
	GOBP_PROTEIN_ADP_RIBOSYLATION	0.035714

	GOBP_PTERIDINE_CONTAINING_COMPOUND_METABOLIC_PROCESS	0.035714
	GOBP_PURINE_NUCLEOSIDE_MONOPHOSPHATE_METABOLIC_PROCESS	0.035714
	GOBP_QUINONE_METABOLIC_PROCESS	0.035714
	GOBP_REGULATION_OF_DEFENSE_RESPONSE_TO_VIRUS_BY_HOST	0.035714
	GOBP_REGULATION_OF_TYPE_I_INTERFERON_MEDIATED_SIGNALING_PATHWAY	0.035714
	GOBP_RNA_PHOSPHODIESTER_BOND_HYDROLYSIS_EXONUCLEOLYTIC	0.035714
	GOBP_RNA_POLYADENYLATION	0.035714
	GOBP_SERINE_FAMILY_AMINO_ACID_METABOLIC_PROCESS	0.035714

	GOBP_TELOMERE_CAPPING	0.035714
	GOBP_TRANSCRIPTION_PREINITIATION_COMPLEX_ASSEMBLY	0.035714
	GOCC_DNA_PACKAGING_COMPLEX	0.035714
	GOCC_NADH_DEHYDROGENASE_COMPLEX	0.035714
	GOCC_NUCLEAR_UBIQUITIN_LIGASE_COMPLEX	0.035714
	GOMF_LIGASE_ACTIVITY_FORMING_CARBON_NITROGEN_BONDS	0.035714
	GOMF_LIGASE_ACTIVITY_FORMING_CARBON_SULFUR_BONDS	0.035714
	GOMF_RNA_METHYLTRANSFERASE_ACTIVITY	0.035714

	GOMF_SNRNA_BINDING	0.035714
	GOBP_NEGATIVE_REGULATION_OF_LEUKOCYTE_APOPTOTIC_PROCESS	0.036145
	GOBP_POSITIVE_REGULATION_OF_WOUND_HEALING	0.036145
	GOBP_PROSTATE_GLAND_DEVELOPMENT	0.036145
	GOBP_COCHLEA_DEVELOPMENT	0.037037
	GOBP_POSITIVE_REGULATION_OF_CELLULAR_CARBOHYDRATE_METABOLIC_PROCESS	0.037037
	GOBP_RESPONSE_TO_NICOTINE	0.037037
	GOBP_NEGATIVE_REGULATION_OF_BLOOD_PRESSURE	0.038462

	GOBP_RESPONSE_TO_NERVE_GROWTH_FACTOR	0.038462
	GOMF_CARBOXYPEPTIDASE_ACTIVITY	0.038462
	GOMF_NEUROPEPTIDE_RECEPTOR_ACTIVITY	0.038462
	GOBP_CELL_SUBSTRATE_ADHESION	0.04
	GOBP_MORPHOGENESIS_OF_AN_EPITHELIUM	0.04
	GOBP_NEGATIVE_REGULATION_OF_LOCOMOTION	0.04
	GOBP_REGULATION_OF_WNT_SIGNALING_PATHWAY	0.04
	GOBP_ADENYLATE_CYCLASE_MODULATING_G_PROTEIN_COUPLED_RECEPTOR_SIGNALING_PATHWAY	0.040404
	GOBP_ANTERIOR_POSTERIOR_PATTERN_SPECIFICATION	0.040404
	GOBP_BONE_DEVELOPMENT	0.040404
	GOBP_CALCIUM_MEDIATED_SIGNALING	0.040404

	GOBP_CARDIAC_CHAMBER_DEVELOPMENT	0.040404
	GOBP_CARDIAC_MUSCLE_TISSUE_DEVELOPMENT	0.040404
	GOBP_CELL_CELL_JUNCTION_ORGANIZATION	0.040404
	GOBP_CELL_MATRIX_ADHESION	0.040404
	GOBP_CENTRAL_NERVOUS_SYSTEM_NEURON_DIFFERENTIATION	0.040404
	GOBP_DENDRITE_DEVELOPMENT	0.040404
	GOBP_DEVELOPMENT_OF_PRIMARY_SEXUAL_CHARACTERISTICS	0.040404
	GOBP_DEVELOPMENTAL_CELL_GROWTH	0.040404

	GOBP_EAR_DEVELOPMENT	0.040404
	GOBP_ENDOTHELIAL_CELL_MIGRATION	0.040404
	GOBP_FAT_CELL_DIFFERENTIATION	0.040404
	GOBP_GLIAL_CELL_DIFFERENTIATION	0.040404
	GOBP_HEMOSTASIS	0.040404
	GOBP_INOSITOL_LIPID_MEDIATED_SIGNALING	0.040404
	GOBP_MAINTENANCE_OF_LOCATION_IN_CELL	0.040404
	GOBP_MORPHOGENESIS_OF_A_BRANCHING_STRUCTURE	0.040404

	GOBP_MULTI_MULTICELLULAR_ORGANISM_PROCESS	0.040404
	GOBP_MUSCLE_CELL_PROLIFERATION	0.040404
	GOBP_MYELOID_LEUKOCYTE_MIGRATION	0.040404
	GOBP_NEGATIVE_REGULATION_OF_CELL_DEVELOPMENT	0.040404
	GOBP_NEUROTRANSMITTER_TRANSPORT	0.040404
	GOBP_NOTCH_SIGNALING_PATHWAY	0.040404
	GOBP_OSTEOBLAST_DIFFERENTIATION	0.040404
	GOBP_POSITIVE_REGULATION_OF_NEUROGENESIS	0.040404

	GOBP_POSITIVE_REGULATION_OF_SUPRAMOLECULAR_FIBER_ORGANIZATION	0.040404
	GOBP_PROTEIN_PROCESSING	0.040404
	GOBP_REGULATION_OF_CALCIUM_ION_TRANSPORT	0.040404
	GOBP_REGULATION_OF_CELL_JUNCTION_ASSEMBLY	0.040404
	GOBP_REGULATION_OF_CELL_SUBSTRATE_ADHESION	0.040404
	GOBP_REGULATION_OF_CHEMOTAXIS	0.040404
	GOBP_REGULATION_OF_ENDOCYTOSIS	0.040404
	GOBP_REGULATION_OF_ENDOTHELIAL_CELL_MIGRATION	0.040404

	GOBP_REGULATION_OF_EPITHELIAL_CELL_MIGRATION	0.04<br>040<br>4
	GOBP_REGULATION_OF_HEART_CONTRACTION	0.04<br>040<br>4
	GOBP_REGULATION_OF_LEUKOCYTE_MIGRATION	0.04<br>040<br>4
	GOBP_REGULATION_OF_MUSCLE_SYSTEM_PROCESS	0.04<br>040<br>4
	GOBP_REGULATION_OF_NEUROTRANSMITTER_LEVELS	0.04<br>040<br>4
	GOBP_REGULATION_OF_PROTEIN_BINDING	0.04<br>040<br>4
	GOBP_REGULATION_OF_SYNAPSE_STRUCTURE_OR_ACTIVITY	0.04<br>040<br>4
	GOBP_RESPIRATORY_SYSTEM_DEVELOPMENT	0.04<br>040<br>4

	GOBP_RESPONSE_TO_BMP	0.04<br>040<br>4
	GOBP_RESPONSE_TO_MECHANICAL_STIMULUS	0.04<br>040<br>4
	GOBP_SKELETAL_SYSTEM_MORPHOGENESIS	0.04<br>040<br>4
	GOBP_STEM_CELL_DIFFERENTIATION	0.04<br>040<br>4
	GOBP_STRIATED_MUSCLE_CONTRACTION	0.04<br>040<br>4
	GOBP_TRANSFORMING_GROWTH_FACTOR_BETA_RECEPTOR_SIGN<br>ALING_PATHWAY	0.04<br>040<br>4
	GOBP_VESICLE_MEDIATED_TRANSPORT_IN_SYNAPSE	0.04<br>040<br>4
	GOCC_CATION_CHANNEL_COMPLEX	0.04<br>040<br>4

	GOCC_EXOCYTIC_VESICLE	0.040404
	GOCC_EXTRINSIC_COMPONENT_OF_PLASMA_MEMBRANE	0.040404
	GOCC_LAMELLIPODIUM	0.040404
	GOCC_LEADING_EDGE_MEMBRANE	0.040404
	GOMF_CALMODULIN_BINDING	0.040404
	GOMF_GLYCOSAMINOGLYCAN_BINDING	0.040404
	GOMF_PEPTIDASE_REGULATOR_ACTIVITY	0.040404
	GOBP_FIBROBLAST_MIGRATION	0.040541

	GOBP_FOREBRAIN_NEURON_DIFFERENTIATION	0.040541
	GOMF_ALPHA_TUBULIN_BINDING	0.040541
	GOBP_ACTOMYOSIN_STRUCTURE_ORGANIZATION	0.040816
	GOBP_APPENDAGE_DEVELOPMENT	0.040816
	GOBP_CARTILAGE_DEVELOPMENT	0.040816
	GOBP_LOCOMOTORY_BEHAVIOR	0.040816
	GOBP_MUSCLE_CELL_DEVELOPMENT	0.040816
	GOBP_NEURON_PROJECTION_EXTENSION	0.040816

	GOBP_POSITIVE_REGULATION_OF_CYTOSKELETON_ORGANIZATION	0.040816
	GOBP_POSITIVE_REGULATION_OF_EPITHELIAL_CELL_PROLIFERATION	0.040816
	GOBP_POSITIVE_REGULATION_OF_ERK1_AND_ERK2_CASCADE	0.040816
	GOBP_POSITIVE_REGULATION_OF_PEPTIDYL_TYROSINE_PHOSPHORYLATION	0.040816
	GOBP_POSITIVE_REGULATION_OF_TRANSMEMBRANE_TRANSPORT	0.040816
	GOBP_PROTEIN_KINASE_B_SIGNALING	0.040816
	GOBP_REGULATION_OF_MAP_KINASE_ACTIVITY	0.040816
	GOBP_REGULATION_OF_SYNAPTIC_PLASTICITY	0.040816

	GOBP_RESPONSE_TO_KETONE	0.04 081 6
	GOBP_SYNAPSE_ASSEMBLY	0.04 081 6
	GOCC_CYTOPLASMIC_SIDE_OF_MEMBRANE	0.04 081 6
	GOMF_ENDOPEPTIDASE_REGULATOR_ACTIVITY	0.04 081 6
	GOMF_VOLTAGE_GATED_CHANNEL_ACTIVITY	0.04 081 6
	GOBP_CARDIAC_CONDUCTION_SYSTEM_DEVELOPMENT	0.04 109 6
	GOBP_NEGATIVE_REGULATION_OF_INTRACELLULAR_PROTEIN_TR ANSPORT	0.04 109 6
	GOBP_NEGATIVE_REGULATION_OF_PLASMA_MEMBRANE_BOUND ED_CELL_PROJECTION_ASSEMBLY	0.04 109 6

	GOBP_POSITIVE_REGULATION_OF_NOTCH_SIGNALING_PATHWAY	0.04<br>109<br>6
	GOBP_REGULATION_OF_NEUROBLAST_PROLIFERATION	0.04<br>109<br>6
	GOMF_G_PROTEIN_ACTIVITY	0.04<br>109<br>6
	GOMF_ISOPRENOID_BINDING	0.04<br>109<br>6
	GOMF_PROTEIN_LIPID_COMPLEX_BINDING	0.04<br>109<br>6
	GOBP_ACTIN_POLYMERIZATION_OR_DEPOLYMERIZATION	0.04<br>123<br>7
	GOBP_GASTRULATION	0.04<br>123<br>7
	GOBP_NEGATIVE_REGULATION_OF_CELL_GROWTH	0.04<br>123<br>7

	GOBP_NEGATIVE_REGULATION_OF_CELL_PROJECTION_ORGANIZATION	0.041237
	GOBP_REGENERATION	0.041237
	GOBP_REGULATION_OF_BLOOD_PRESSURE	0.041237
	GOBP_REGULATION_OF_CELL_SIZE	0.041237
	GOBP_REGULATION_OF_NEURON_DIFFERENTIATION	0.041237
	GOBP_REGULATION_OF_PROTEIN_POLYMERIZATION	0.041237
	GOBP_BASE_EXCISION_REPAIR	0.041667
	GOBP_EAR_MORPHOGENESIS	0.041667

	GOBP_ER_NUCLEUS_SIGNALING_PATHWAY	0.04<br>166<br>7
	GOBP_NEGATIVE_REGULATION_OF_METAPHASE_ANAPHASE_TRA<br>NSITION_OF_CELL_CYCLE	0.04<br>166<br>7
	GOBP_POSITIVE_REGULATION_OF_MITOTIC_NUCLEAR_DIVISION	0.04<br>166<br>7
	GOBP_PROGRAMMED_NECROTIC_CELL_DEATH	0.04<br>166<br>7
	GOBP_PROTEIN_EXIT_FROM_ENDOPLASMIC_RETICULUM	0.04<br>166<br>7
	GOBP_REGULATION_OF_CENTROSOME_CYCLE	0.04<br>166<br>7
	GOBP_RESPONSE_TO_ANTIBIOTIC	0.04<br>166<br>7
	GOBP_SISTER_CHROMATID_COHESION	0.04<br>166<br>7

	GOMF_3_5_EXONUCLEASE_ACTIVITY	0.04<br>166<br>7
	GOMF_CYCLIN_DEPENDENT_PROTEIN_SERINE_THREONINE_KINASE_REGULATOR_ACTIVITY	0.04<br>166<br>7
	GOMF_GENERAL_TRANSCRIPTION_INITIATION_FACTOR_ACTIVITY	0.04<br>166<br>7
	GOMF_OXIDOREDUCTASE_ACTIVITY_ACTING_ON_NAD_P_H_QUINONE_OR_SIMILAR_COMPOUND_AS_ACCEPTOR	0.04<br>166<br>7
	GOMF_PENTOSYLTRANSFERASE_ACTIVITY	0.04<br>166<br>7
	GOBP_REGULATION_OF_EXTRINSIC_APOPTOTIC_SIGNALING_PATHWAY	0.04<br>210<br>5
	GOBP_REGULATION_OF_SIGNALING_RECEPTOR_ACTIVITY	0.04<br>210<br>5
	GOBP_ADRENERGIC_RECEPTOR_SIGNALING_PATHWAY	0.04<br>225<br>4

	GOMF_INTRACELLULAR_LIGAND_GATED_ION_CHANNEL_ACTIVITY	0.042254
	GOMF_METALLOCARBOXYPEPTIDASE_ACTIVITY	0.042254
	GOBP_NEGATIVE_REGULATION_OF_NERVOUS_SYSTEM_DEVELOPMENT	0.042553
	GOBP_POSITIVE_REGULATION_OF_CELL_GROWTH	0.042553
	GOBP_POSITIVE_REGULATION_OF_LEUKOCYTE_MIGRATION	0.042553
	GOBP_REGULATION_OF_ACTIN_FILAMENT_LENGTH	0.042553
	GOBP_REGULATION_OF_DENDRITE_DEVELOPMENT	0.042553
	GOBP_REGULATION_OF_PEPTIDYL_SERINE_PHOSPHORYLATION	0.042553

	GOBP_REGULATION_OF_SYSTEMIC_ARTERIAL_BLOOD_PRESSURE	0.042553
	GOBP_SYNAPTIC_VESICLE_EXOCYTOSIS	0.042553
	GOMF_NEUROPEPTIDE_RECEPTOR_BINDING	0.042857
	GOBP_ACTIN_CYTOSKELETON_REORGANIZATION	0.043011
	GOBP_NEGATIVE_REGULATION_OF_EXTRINSIC_APOPTOTIC_SIGNALING_PATHWAY	0.043011
	GOBP_EPITHELIAL_CELL_APOPTOTIC_PROCESS	0.043478
	GOBP_FEEDING_BEHAVIOR	0.043478
	GOBP_NEURAL_TUBE_FORMATION	0.043478

	GOBP_NEUROTRANSMITTER_METABOLIC_PROCESS	0.04 347 8
	GOCC_SYNAPTIC_VESICLE_MEMBRANE	0.04 395 6
	GOBP_POSITIVE_REGULATION_OF_SMOOTH_MUSCLE_CONTRACTI ON	0.04 411 8
	GOBP_PROSTATE_GLAND_MORPHOGENESIS	0.04 411 8
	GOMF_CHEMOREPELLENT_ACTIVITY	0.04 411 8
	GOBP_NEURON_FATE_COMMITMENT	0.04 444 4
	GOBP_ODONTOGENESIS_OF_DENTIN_CONTAINING_TOOTH	0.04 444 4
	GOBP_INTEGRIN_ACTIVATION	0.04 477 6

	GOBP_MEMBRANE_REPOLARIZATION_DURING_ACTION_POTENTIAL	0.044776
	GOBP_ORGAN_INDUCTION	0.044776
	GOBP_REGULATION_OF_POSITIVE_CHEMOTAXIS	0.044776
	GOBP_REGULATION_OF_SYSTEMIC_ARTERIAL_BLOOD_PRESSURE_BY_RENIN_ANGIOTENSIN	0.044776
	GOBP_RESPONSE_TO_AUDITORY_STIMULUS	0.044776
	GOBP_SYNAPTIC_TRANSMISSION_CHOLINERGIC	0.044776
	GOMF_LAMININ_BINDING	0.044776
	GOBP_EXPORT_ACROSS_PLASMA_MEMBRANE	0.044944

	GOBP_EXTRINSIC_APOPTOTIC_SIGNALING_PATHWAY_VIA_DEATH_DOMAIN_RECEPTORS	0.044944
	GOBP_POSITIVE_REGULATION_OF_PLASMA_MEMBRANE_BOUNDED_CELL_PROJECTION_ASSEMBLY	0.044944
	GOBP_REGULATION_OF_OSTEOCLAST_DIFFERENTIATION	0.044944
	GOBP_ANATOMICAL_STRUCTURE_REGRESSION	0.045455
	GOBP_GLUCOSE_IMPORT	0.045455
	GOBP_NEGATIVE_REGULATION_OF_MYOTUBE_DIFFERENTIATION	0.045455
	GOBP_NEGATIVE_REGULATION_OF_OLIGODENDROCYTE_DIFFERENTIATION	0.045455
	GOBP_REACTIVE_NITROGEN_SPECIES_METABOLIC_PROCESS	0.045455

	GOBP_REGULATION_OF_NATURAL_KILLER_CELL_MEDIATED_IMMUNITY	0.045455
	GOBP_REGULATION_OF_TELOMERASE_ACTIVITY	0.045455
	GOCC_AXOLEMMA	0.045455
	GOMF_CARBON_CARBON_LYASE_ACTIVITY	0.045455
	GOMF_MONOCARBOXYLIC_ACID_BINDING	0.045455
	GOMF_NITRIC_OXIDE_SYNTHASE_BINDING	0.045455
	GOMF_RNA_STEM_LOOP_BINDING	0.045455
	GOBP_HETEROTYPIC_CELL_CELL_ADHESION	0.045977

	GOBP_LAMELLIPODIUM_ASSEMBLY	0.04<br>597<br>7
	GOBP_MORPHOGENESIS_OF_AN_EPITHELIAL_SHEET	0.04<br>597<br>7
	GOBP_ATRIAL_SEPTUM_MORPHOGENESIS	0.04<br>615<br>4
	GOBP_BONE_TRABECULA_MORPHOGENESIS	0.04<br>615<br>4
	GOBP_FIBROBLAST_ACTIVATION	0.04<br>615<br>4
	GOBP_MESODERMAL_CELL_FATE_SPECIFICATION	0.04<br>615<br>4
	GOBP_MOTOR_BEHAVIOR	0.04<br>615<br>4
	GOBP_NEGATIVE_REGULATION_OF_INTRACELLULAR_ESTROGEN_<br>RECEPTOR_SIGNALING_PATHWAY	0.04<br>615<br>4

	GOBP_NEGATIVE_REGULATION_OF_SMOOTH_MUSCLE_CONTRACTION	0.046154
	GOBP_POSITIVE_REGULATION_OF_CARDIOCYTE_DIFFERENTIATION	0.046154
	GOBP_REGULATION_OF_ACROSOME_REACTION	0.046154
	GOBP_REGULATION_OF_FIBRINOLYSIS	0.046154
	GOCC_APICAL_DENDRITE	0.046154
	GOBP_ARGININE_METABOLIC_PROCESS	0.046512
	GOBP_EPOXYGENASE_P450_PATHWAY	0.046512
	GOBP_MRNA_CIS_SPLICING_VIA_SPLICEOSOME	0.046512

	GOBP_P_BODY_ASSEMBLY	0.04651 2
	GOBP_PEPTIDYL_LYSINE_DIMETHYLATION	0.04651 2
	GOBP_PROTEASOME_ASSEMBLY	0.04651 2
	GOBP_REGULATION_OF_DEOXYRIBONUCLEASE_ACTIVITY	0.04651 2
	GOBP_REGULATION_OF_FC_RECEPTOR_MEDIATED_STIMULATORY_SIGNALING_PATHWAY	0.04651 2
	GOBP_TRNA_5_LEADER_REMOVAL	0.04651 2
	GOCC_ENDOLYSOSOME_MEMBRANE	0.04651 2
	GOCC_NUCLEOCYTOPLASMIC_TRANSPORT_COMPLEX	0.04651 2

	GOMF_METAL_ION_PROTON_ANTIPORTER_ACTIVITY	0.04 651 2
	GOMF_UBIQUITIN_PROTEIN_TRANSFERASE_REGULATOR_ACTIVITY	0.04 651 2
	GOBP_LOCOMOTORY_EXPLORATION_BEHAVIOR	0.04 687 5
	GOBP_MEGAKARYOCYTE_DEVELOPMENT	0.04 687 5
	GOBP_METANEPHRIC_RENAL_VESICLE_MORPHOGENESIS	0.04 687 5
	GOBP_NOSE_DEVELOPMENT	0.04 687 5
	GOBP_POSITIVE_REGULATION_OF_MYELINATION	0.04 687 5
	GOBP_REGULATION_OF_VASCULAR_ASSOCIATED_SMOOTH_MUSCLE_CELL_DIFFERENTIATION	0.04 687 5

	GOBP_RESPONSE_TO_POTASSIUM_ION	0.04<br>687<br>5
	GOMF_CGMP_BINDING	0.04<br>687<br>5
	GOBP_REGULATION_OF_SYSTEMIC_ARTERIAL_BLOOD_PRESSURE_<br>MEDIATED_BY_A_CHEMICAL_SIGNAL	0.04<br>705<br>9
	GOBP_COCHLEA_MORPHOGENESIS	0.04<br>761<br>9
	GOBP_HOMOLOGOUS_RECOMBINATION	0.04<br>761<br>9
	GOBP_MITOTIC_METAPHASE_PLATE_CONGRESSION	0.04<br>761<br>9
	GOBP_NCRNA_TRANSCRIPTION	0.04<br>761<br>9
	GOBP_NEGATIVE_REGULATION_OF_CELL_CYCLE_G2_M_PHASE_TR<br>ANSITION	0.04<br>761<br>9

	GOBP_NEGATIVE_REGULATION_OF_PROTEIN_MATURATION	0.047619
	GOBP_POSITIVE_REGULATION_OF_DOUBLE_STRAND_BREAK_REPAIR	0.047619
	GOBP_REGULATION_OF_MEIOTIC_CELL_CYCLE	0.047619
	GOBP_REGULATION_OF_SKELETAL_MUSCLE_TISSUE_DEVELOPMENT	0.047619
	GOBP_SYNAPSE_MATURATION	0.047619
	GOBP_TRANSCRIPTION_BY_RNA_POLYMERASE_I	0.047619
	GOCC_EUCHROMATIN	0.047619
	GOCC_KINESIN_COMPLEX	0.047619

	GOMF_CYTOSKELETAL_ANCHOR_ACTIVITY	0.047619
	GOMF_DEOXYRIBONUCLEASE_ACTIVITY	0.047619
	GOMF_RNA_POLYMERASE_BINDING	0.047619
	GOMF_TRNA_BINDING	0.047619
	GOBP_EOSINOPHIL_MIGRATION	0.048387
	GOBP_PEROXISOME_PROLIFERATOR_ACTIVATED_RECEPTOR_SIGNALING_PATHWAY	0.048387
	GOMF_G_PROTEIN_BETA_SUBUNIT_BINDING	0.048387
	GOBP_ANTIVIRAL_INNATE_IMMUNE_RESPONSE	0.04878
	GOBP_NADPH_REGENERATION	0.04878

	GOMF_C4_DICARBOXYLATE_TRANSMEMBRANE_TRANSPORTER_ACTIVITY	0.04878
	GOMF_RNA_POLYMERASE_II_CORE_PROMOTER_SEQUENCE_SPECIFIC_DNA_BINDING	0.04878
	GOBP_TERPENOID_BIOSYNTHETIC_PROCESS	0.04918
